# Weak annihilation and new physics in charmless $\varvec{B\rightarrow M M}$ decays

**DOI:** 10.1140/epjc/s10052-015-3535-1

**Published:** 2015-07-22

**Authors:** Christoph Bobeth, Martin Gorbahn, Stefan Vickers

**Affiliations:** Technische Universität München, Institute for Advanced Study, Lichtenbergstraße 2a, 85748 Garching, Germany; Department of Mathematical Sciences, University of Liverpool, Liverpool, L69 3BX UK; Technische Universität München, Excellence Cluster Universe, Boltzmannstraße 2, 85748 Garching, Germany

## Abstract

We use currently available data of nonleptonic charmless 2-body $B\rightarrow MM$ decays ($MM \!=\! PP, PV, VV$) that are mediated by $b\rightarrow (d, s)$ QCD- and QED-penguin operators to study weak annihilation and new-physics effects in the framework of QCD factorization. In particular we introduce one weak-annihilation parameter for decays related by $(u\leftrightarrow d)$ quark interchange and test this universality assumption. Within the standard model, the data supports this assumption with the only exceptions in the $B\rightarrow K \pi$ system, which exhibits the well-known “${\varDelta } {\mathcal{A}_\mathrm{CP}}$ puzzle”, and some tensions in $B \rightarrow K^* \phi$. Beyond the standard model, we simultaneously determine weak-annihilation and new-physics parameters from data, employing model-independent scenarios that address the “${\varDelta } {\mathcal{A}_\mathrm{CP}}$ puzzle”, such as QED-penguins and $b\rightarrow s\, \bar{u}u$ current-current operators. We discuss also possibilities that allow further tests of our assumption once improved measurements from LHCb and Belle II become available.

## Introduction

Nonleptonic charmless 2-body decays $B\rightarrow M M$, with final state mesons $MM = (PP,\, PV,\, VV)$, form a large class of decays that allow one to test in principle the underlying tree and penguin topologies at the parton level, as predicted by the standard model (SM). Further, the subclass of QCD- and QED-penguin-dominated decays are sensitive to new physics (NP) beyond the SM, as any other $b \rightarrow (d,s)$ flavor-changing neutral-current (FCNC) process, which makes them valuable probes of the according short-distance couplings.

The major obstacle to constraining the short-distance couplings with data is the evaluation of hadronic matrix elements in 2-body *B*-meson decays beyond naive factorization. In view of this, strategies have been developed to construct tests of the weak phases of the Cabibbo–Kobayashi–Maskawa (CKM) quark-mixing matrix of the SM where the hadronic matrix elements are determined from data, usually involving additional assumptions of *SU*(2) and/or *SU*(3) flavor symmetries. Although this allows one to test the consistency of weak phases extracted in tree- and loop-induced processes in the framework of the SM, no other detailed information can be obtained on particular short-distance couplings of the involved QCD- and QED-penguin operators.

In this respect, systematic expansions in the heavy bottom quark mass, $m_b$, yield at leading order in $1/m_b$ a simplified representation of hadronic 2-body matrix elements in terms of rather well-known heavy-to-light form factors and distribution amplitudes (DA) of the involved mesons. These approaches, QCD factorization (QCDF) [1–6], soft-collinear effective theory (SCET) [7–10] or perturbative QCD (pQCD) [11–14], provide predictions at leading order in $1/m_b$ that allow one in principle to test short-distance couplings with data.

Weak annihilation (WA) contributions are formally of subleading order in $1/m_b$, but an additional chiral enhancement makes them phenomenologically relevant for a consistent description of experimental data in the SM and scenarios beyond. In QCDF and SCET, they are plagued by nonfactorizable divergences, which are present in endpoint regions of convolutions of meson DAs. In QCDF, these divergences are frequently parameterized by a phenomenological complex parameter [3] and hence are model-dependent. In particular, the associated strong phase governs the size of CP asymmetries. In practice this leads to large theoretical uncertainties in the prediction of observables [5, 15]. Although branching fractions and CP asymmetries are sensitive to new-physics effects, the model-dependence and the arising uncertainties due to the involved strong phases raise the question how reliable information can be extracted on the short-distance couplings.

Here we determine the model-dependence in the framework of QCDF from data, admitting one phenomenological parameter for decays $$B \rightarrow M_a M_b$$ that are related by $$(u \leftrightarrow d$$) quark exchange. The theoretical uncertainties of all other input parameters (see Appendix A) are treated as uncorrelated and have been included into the likelihood as explained in Appendix B. We use data of mostly QCD-penguin dominated $$B_{u,d}$$ decays into $$PP = K\pi ,\, K\eta ^{(')},\, KK$$ or $$PV = K\rho ,\, K\phi ,\, K\omega ,\, K^*\pi ,\, K^*\eta ^{(')}$$ or $$VV = K^*\rho ,\, K^*\phi ,\, K^*\omega ,$$$$K^*K^*$$, and further $$B_s$$ decays into $$PP = \pi \pi ,\, KK,\, K\pi $$ or $$VV = \phi \phi ,\, K^*\phi ,\, K^* K^*$$ final states. We determine also the relative magnitude of subleading WA amplitudes compared to the relevant leading order amplitudes. The results within the context of the SM are presented in Sect. 4. Given the current data, a simultaneous fit of the WA parameters and the short-distance couplings is pursued in Sect. 5 for generic NP extensions of the SM in order to explore the constraining power of these decays. Before presenting our results of the fit, we review the observables and collect the experimental input of charmless 2-body decays in Sect. 2. The relevant details of QCDF and the definition of the phenomenological parameter are summarized in Sect. 3. Various appendices collect additional material on numerical input in Appendix A and the statistical treatment of experimental and theoretical uncertainties as well as determination of pull values and *p* values in Appendix B.

## $B \rightarrow MM$ observables and data

The 2-body decays of *B* mesons into final states $f = PP,\, PV,\, V_h V_h$ with light charmless pseudoscalar (*P*) and/or vector ($V_h$) mesons with polarization mode $h = L, \perp , \parallel$ provide various observables in time-integrated, time-dependent, and also angular analyses. These are reviewed in the first part of this section, whereas in the second part the according available experimental data is listed that has been used in the fits.

### Observables

The most important observables for decays of charged $B_u$ mesons into a final state *f* are the CP-averaged branching fraction and the (direct) CP asymmetry

2.1 $$\begin{aligned} \overline{\mathcal{B}} {\left[ B_u \rightarrow f \right] }&= \frac{\tau _{B_u}}{2} \left( {\varGamma }[\bar{B}_u \rightarrow \bar{f}] + {\varGamma }[B_u \rightarrow f] \right) , \nonumber \\ \mathcal{A}_\mathrm{CP} {\left[ B_u \rightarrow f \right] }&= \frac{{\varGamma }[\bar{B}_u \rightarrow \bar{f}] - {\varGamma }[B_u \rightarrow f]}{{\varGamma }[\bar{B}_u \rightarrow \bar{f}] + {\varGamma }[B_u \rightarrow f]} . \end{aligned}$$

Concerning decays of neutral $B_D$ mesons ($D = d, s$) into a common final state *f* for both flavor eigenstates $\bar{B}_D$ and $B_D$, the simplest measurements are untagged rates. The decay is governed by the decay rates $R_f^\mathrm{H,L} \equiv {\varGamma }[B_D^\mathrm{H,L} \rightarrow f]$ of the heavy and light mass eigenstates $B_D^\mathrm{H, L}$, which yield the averaged and time-integrated branching fraction

2.2 $$\begin{aligned} \overline{\mathcal{B}} {\left[ B_D \rightarrow f \right] }&= \frac{1}{2} \int \mathrm{d}t \left( R_f^\mathrm{L} \, e^{- {\varGamma }^\mathrm{L}_D t} + R_f^\mathrm{H} \, e^{- {\varGamma }^\mathrm{H}_D t} \right) \nonumber \\&= \frac{1}{2} \left( \frac{R_f^\mathrm{L}}{{\varGamma }^\mathrm{L}_D} + \frac{R_f^\mathrm{H}}{{\varGamma }^\mathrm{H}_D} \right) , \end{aligned}$$

with their respective lifetimes ${\varGamma }_D^\mathrm{H,L}$ in the two exponentials. The mass eigenstates are related to the flavor eigenstates $|B_D^\mathrm{L} \rangle = p |B_D \rangle + q |\bar{B}_D\rangle$ and $|B_D^\mathrm{H} \rangle = p |B_D \rangle - q |\bar{B}_D\rangle$ defining *q* and *p*. On the other hand, theoretical predictions are made for the flavor eigenstates, implying $t = 0$,

2.3 $$\begin{aligned} \mathcal{B}[B_D \rightarrow f]&= \frac{\frac{1}{2} \left( {\varGamma }[\bar{B}_D \rightarrow f] + {\varGamma }[B_D \rightarrow f]\right) }{\frac{1}{2} \left( {\varGamma }^\mathrm{L}_D + {\varGamma }^\mathrm{H}_D \right) } \end{aligned}$$

with the average lifetime $\tau _{B_D} = ({\varGamma }_D)^{-1} \equiv 2/({\varGamma }^\mathrm{L}_D + {\varGamma }^\mathrm{H}_D)$. Both branching fractions are related via [16]

2.4 $$\begin{aligned} \overline{\mathcal{B}} {\left[ B_D \rightarrow f \right] }&= \mathcal{B}[B_D \rightarrow f] \, \frac{1 + y_D \, H_f}{1 - y_D^2} \end{aligned}$$

where $y_D$ is proportional to the width difference ${\varDelta } {\varGamma }_D$

2.5 $$\begin{aligned} y_D&= \frac{{\varDelta }{\varGamma }_D}{2 {\varGamma }_D} \equiv \frac{{\varGamma }^\mathrm{L}_D - {\varGamma }^\mathrm{H}_D}{{\varGamma }^\mathrm{L}_D + {\varGamma }^\mathrm{H}_D} . \end{aligned}$$

The CP asymmetry due to nonvanishing width difference,

2.6 $$\begin{aligned} H_f&= \frac{R_f^\mathrm{H} - R_f^\mathrm{L}}{R_f^\mathrm{H} + R_f^\mathrm{L}}, \end{aligned}$$

is an independent observable and provides complementary tests of physics beyond the SM. It can be obtained from measurements of effective lifetimes in untagged, but time-dependent rate measurements [17] or together with the mixing-induced CP asymmetry $S_f$ and the direct CP asymmetry $C_f = - {\mathcal{A}_\mathrm{CP}}$ of a time-dependent analysis

2.7 $$\begin{aligned} \mathcal{A}_\mathrm{CP} {\left[ B_D\rightarrow f \right] }(t)&= \frac{S_f \sin ({\varDelta } m_D\, t) - C_f \cos ({\varDelta } m_D\, t)}{\cosh \left( \frac{{\varDelta } {\varGamma }_D}{2} t\right) - H_f \sinh \left( \frac{{\varDelta } {\varGamma }_D}{2} t\right) }, \end{aligned}$$

where the mass difference of the heavy and light mass eigenstates is denoted as ${\varDelta } m_D = m_D^\mathrm{H} - m_D^\mathrm{L} > 0$. The three CP asymmetries are not independent of each other, $|S_f|^2 +|C_f|^2+|H_f|^2 = 1$, and they are given in terms of one complex quantity

2.8 $$\begin{aligned} \lambda _f= & {} \frac{q}{p} \frac{\overline{A}_f}{A_f}, \end{aligned}$$

as follows:

2.9 $$\begin{aligned} S_f= & {} \frac{2\, \text{ Im }(\lambda _f)}{1+\left| \lambda _f\right| ^2}, \quad H_f = \frac{2\, \text{ Re }(\lambda _f)}{1+\left| \lambda _f\right| ^2}, \nonumber \\ C_f= & {} \frac{1-\left| \lambda _f\right| ^2}{1+\left| \lambda _f\right| ^2}. \end{aligned}$$

In Eq. (2.8) $\overline{A}_f = A[\bar{B}_D\rightarrow f]$ and $A_f = A[B_D\rightarrow f]$ denote the decay amplitudes and in Sect. 3 we review their calculation in QCDF.

In the limit ${\varDelta } {\varGamma }_D \rightarrow 0$ one obtains $\overline{\mathcal{B}} {\left[ B_D \rightarrow f \right] } = \mathcal{B}[B_D \rightarrow f]$. This is the case for $D = d$ to a very good approximation in the SM where $y_d|_\mathrm{SM}= (0.21 \pm 0.04) \cdot 10^{-2} \ll 1$ [18]. Currently, the precision of experimental results does not yet allow one to test the SM prediction. Measurements are available from *B*-factories $|y_d| = (0.7 \pm 0.9) \cdot 10^{-2}$ [19] and a recent determination of LHCb from effective lifetimes $y_d = (-2.2 \pm 1.4)\cdot 10^{-2}$ [20], which assumes the SM result for $H_f$. Model-independent analysis of effects of NP in ${\varDelta } {\varGamma }_d$ show that there is still room for huge nonstandard contributions [21]. We use the approximation $y_d = 0$ in all our predictions, which is well justified in the SM and also the considered NP scenarios.

On the other hand, ${\varDelta } {\varGamma }_s$ is not negligible and the current world average from $B_s \rightarrow J/\psi \phi$ analyses alone is [19]

2.10 $$\begin{aligned} y_s&= (5.8\pm 1.0) \cdot 10^{-2} , \end{aligned}$$

which will be used in our analysis. In general $-1 \le H_f \le 1$, and therefore the correction factor on the r.h.s. of Eq. (2.4) can become of $\mathcal{O}(10~\%)$ for final states *f* that are CP eigenstates, as has been found for some cases [16]. Other averages take into account $B_s \rightarrow J/\psi \pi \pi$ angular analysis, the effective lifetime measurement of $B_s \rightarrow K^+K^-$ and flavor-specific $B_s$ lifetime averages, which involve additional assumptions in the potential presence of new physics. They yield a slightly larger value than in Eq. (2.10), $y_s = (6.2 \pm 0.9) \cdot 10^{-2}$ [19], being consistent within the uncertainties.

Besides branching fractions and CP asymmetries, 2-body decays $B \rightarrow VV$ with subsequent decays $V \rightarrow PP$ provide additional observables in the full angular analysis of the 4-body final state [22]. The decay can be described in terms of three amplitudes, which can be chosen to correspond to definite helicities of the final-state vector mesons $V_{a,h_a} V_{b,h_b}$ with $h_a = h_b = (L, +, -)$ or, as in the following, transversity amplitudes: $A_{\parallel , \perp } = (A_+ \pm A_-)/\sqrt{2}$. The three magnitudes and two relative phases of the $A_h$ can be measured in a threefold angular decay distribution, where we follow the definitions [23]. Hence, five CP-averaged and CP-asymmetric observables can be measured in the case where tagging of the initial *B*-flavor is possible. There are polarization fractions and relative phases for $\bar{B}$ decays

2.11 $$\begin{aligned} f_{h}^{\bar{B}}&= \frac{|\bar{A}_h|^2}{\sum _h |\bar{A}_h|^2}, \quad \phi ^{\bar{B}}_{\parallel , \perp } = \arg \frac{\bar{A}_{\parallel ,\perp }}{\bar{A}_L}. \end{aligned}$$

In view of the normalization condition $f_L^{\bar{B}} + f_\parallel ^{\bar{B}} + f_\perp ^{\bar{B}} = 1$ one uses the branching fraction and two of the polarization fractions. In combination with the same quantities from *B* decays, replacing $\bar{A}_h \rightarrow A_h$, one has three CP-averaged polarization fractions and three CP asymmetries

2.12 $$\begin{aligned} f_h = \frac{1}{2} \left( f_h^{\bar{B}} + f_h^B \right) , \quad {\mathcal{A}_\mathrm{CP}}_h = \frac{f_h^{\bar{B}} - f_h^B}{f_h^{\bar{B}} + f_h^B}. \end{aligned}$$

Concerning the phases, the following two CP-averaged and CP-violating observables can be constructed for $h = (\parallel , \perp )$:

2.13 $$\begin{aligned} \phi _h&= \frac{1}{2} \left( \phi ^{\bar{B}}_h + \phi ^B_h \right) \nonumber \\&\quad - \pi \, \text{ sgn }\left( \phi ^{\bar{B}}_h + \phi ^B_h \right) \theta \left( \phi ^{\bar{B}}_h - \phi ^B_h - \pi \right) , \nonumber \\ {\varDelta } \phi _h&= \frac{1}{2} \left( \phi ^{\bar{B}}_h - \phi ^B_h \right) - \pi \, \theta \left( \phi ^{\bar{B}}_h + \phi ^B_h \right) . \end{aligned}$$

This convention implies $\phi _h = {\varDelta } \phi _h = 0$ at leading order in QCDF, where all strong phases are zero [23] and might differ for the sign of $A_L$ relative to $A_{\parallel ,\perp }$ adopted by experimental collaborations.

In the case of $B_s \rightarrow VV$ decays, again a correction factor Eq. (2.4) due to $y_s \ne 0$ applies, however, now

2.14 $$\begin{aligned} \overline{\mathcal{B}} {\left[ B_D \rightarrow f \right] }&= \sum _{h = L, \parallel , \perp } \!\!\!\!\! \mathcal{B}[B_D \rightarrow f_h] \, \frac{1 + y_D \, H_{f_h}}{1 - y_D^2} , \nonumber \\ \bar{f}_h&= \frac{\mathcal{B}[B_D \rightarrow f_h]}{\displaystyle \sum _{h = L,\parallel , \perp } \!\!\!\!\! \mathcal{B}[B_D \rightarrow f_h]} \, \frac{1 + y_D \, H_{f_h}}{1 - y_D^2}. \end{aligned}$$

Here $H_{f_h}$ is defined as in Eq. (2.9) and the quantity $\lambda _f$ is evaluated with $A_f \rightarrow A_{f_h}$.

Besides these observables, further combinations are considered that involve different types of charged and neutral *B*, $M_a$, and $M_b$ mesons. They are either ratios of branching fractions or differences of direct CP asymmetries. The complete set of ratios [15, 31] is

2.15 $$\begin{aligned} R_c^B= & {} 2\, \frac{\overline{\mathcal{B}} {\left[ B^-\rightarrow M_a^- M_b^0 \right] }}{\overline{\mathcal{B}} {\left[ B^- \rightarrow M_a^0 M_b^- \right] }}, \quad R_c^{M_a} = 2\, \frac{\overline{\mathcal{B}} {\left[ B^-\rightarrow M_a^- M_b^0 \right] }}{\overline{\mathcal{B}} {\left[ \bar{B}^0 \rightarrow M_a^- M_b^+ \right] }}, \nonumber \\ R_c^{M_b}= & {} \frac{\overline{\mathcal{B}} {\left[ B^-\rightarrow M_a^0 M_b^- \right] }}{\overline{\mathcal{B}} {\left[ \bar{B}^0 \rightarrow M_a^- M_b^+ \right] }}, \nonumber \\ R_n^B= & {} \frac{1}{2}\, \frac{\overline{\mathcal{B}} {\left[ \bar{B}^0\rightarrow M_a^- M_b^+ \right] }}{\overline{\mathcal{B}} {\left[ \bar{B}^0 \rightarrow M_a^0 M_b^0 \right] }}, \quad R_n^{M_a} = \frac{1}{2}\, \frac{\overline{\mathcal{B}} {\left[ B^-\rightarrow M_a^0 M_b^- \right] }}{\overline{\mathcal{B}} {\left[ \bar{B}^0 \rightarrow M_a^0 M_b^0 \right] }}, \nonumber \\ R_n^{M_b}= & {} \frac{\overline{\mathcal{B}} {\left[ B^-\rightarrow M_a^- M_b^0 \right] }}{\overline{\mathcal{B}} {\left[ \bar{B}^0 \rightarrow M_a^0 M_b^0 \right] }}, \end{aligned}$$

where factors of $\tau _{B^0}/\tau _{B^-}$ are not included in the definition of $R_{c,n}^{M_a,M_b}$, contrary to [15]. It is anticipated that these ratios are measured directly in experimental analyses, such that common experimental systematic errors cancel. Further, the following two differences of direct CP asymmetries are frequently considered:

2.16 $$\begin{aligned} -{\varDelta } C = {\varDelta } {\mathcal{A}_\mathrm{CP}}= & {} \mathcal{A}_\mathrm{CP} {\left[ B^- \rightarrow M_a^- M_b^0 \right] } - \mathcal{A}_\mathrm{CP} {\left[ \bar{B}^0 \rightarrow M_a^- M_b^+ \right] } , \nonumber \\ {\varDelta } {\mathcal{A}^0_\mathrm{CP}}= & {} \mathcal{A}_\mathrm{CP} {\left[ B^- \rightarrow M_a^0 M_b^- \right] } - \mathcal{A}_\mathrm{CP} {\left[ \bar{B}^0 \rightarrow \, M_a^0 \,M_b^0 \right] },\nonumber \\ \end{aligned}$$

in which in QCDF a cancelation of uncertainties takes place [32].

In order to separate NP effects in decays from those in $B_D-\overline{B}_D$ mixing in $S_f$, we define the observables [33, 34]

2.17 $$\begin{aligned} {\varDelta } S_f&= - \eta _f S_f - \left\{ \begin{array}{ll} S(\bar{B}_d \rightarrow J/\psi \, \bar{K}_S) &{} D = d \\ S(\bar{B}_s \rightarrow J/\psi \, \phi ) &{} D = s \end{array} \right. \end{aligned}$$

with $\eta _f = \pm 1$ the CP eigenvalue of the final state *f*. The decays $\bar{B}_d \rightarrow J/\psi \, \bar{K}_S$ and $\bar{B}_s \rightarrow J/\psi \, \phi$ are dominated by contributions from charm tree-level operators and CP violation in the decay is both parametrically (CKM) and topologically (loop) suppressed. We expect that the CP-violating phase in $B_D-\overline{B}_D$ mixing, $\phi _{B_d}$ and $\phi _{B_s}$, can clearly be extract from those decays, even in the presence of most NP scenarios [35]

2.18 $$\begin{aligned} \lambda _{J/\psi \, \bar{K}_S}&\simeq e^{-i\phi _{B_d}}, \quad S(\bar{B}_d \rightarrow J/\psi \, \bar{K}_S) \simeq \sin 2\beta , \nonumber \\ \lambda _{J/\psi \, \phi }&\simeq e^{-i\phi _{B_s}}, \quad S(\bar{B}_s \rightarrow J/\psi \, \phi ) \simeq \sin 2\beta _s, \end{aligned}$$

in which the angles of the CKM unitarity triangle are defined as $\beta = \arg \left( \lambda _c^d/\lambda _t^d \right)$ and $\beta _s = \arg \left( \lambda _t^s/\lambda _c^s \right)$. This source of CP violation enters the mixing-induced CP asymmetry of most decays that are triggered by $b \rightarrow s$ transition in the same way and can be eliminated by the construction of ${\varDelta } S_f$, which therefore exclusively measures the interference of CP violation in the decay and in mixing.

### Data 

We investigate mainly $B\rightarrow MM$ decays mediated by $b\rightarrow s$ transitions but will consider also some $b\rightarrow d$ examples. The final 2-meson state *MM* consists either out of two pseudoscalars ($MM = PP$) or one pseudoscalar and one vector ($MM = PV$)^1^ or two vectors ($MM = VV$), which are listed in Tables 1, 2, and 3, respectively, together with the observables that have been measured. We use the most recent values of branching ratios $\mathcal{B}$ as well as direct and mixing-induced CP asymmetries ${\mathcal{A}_\mathrm{CP}}= - C$ and *S* from the Heavy Flavor Averaging Group (HFAG) 2012 compilation and updates from 2013/2014 on the website [19]. For decays into *VV* final states we include also the data of polarization fractions $f_{L, \perp }$, the relative phases $\phi _{\parallel ,\perp }$ and CP asymmetries $C_{L,\perp }$. Meanwhile, some observables had been updated or measured for the first time from individual experiments and not yet included in the HFAG averages. In these cases we do not make use of HFAG averages, but instead all measurements from individual experiments enter the likelihood function in Eq. (B3) as single measurements. The according references are given explicitly in the tables for such cases.

**Table 1:** Observables of $B\rightarrow PP$ decays mediated by $b\rightarrow s$ and $b\rightarrow d$ transitions that are used in the fit

$\varvec{b \rightarrow s}$					$\varvec{b \rightarrow d}$	
$B \rightarrow K \pi$	$B \rightarrow K \eta$	$B \rightarrow K \eta '$	$B_s \rightarrow KK$ [24]	$B_s \rightarrow \pi \pi$	$B \rightarrow K K$	$B_s \rightarrow K \pi$
$K^0 \pi ^0 : \mathcal{B},\, C,\, S$	$K^0 {\eta } : \mathcal{B}$	$K^0 {\eta '} : \mathcal{B},\, C,\, S$	$K^+ K^- : \mathcal{B},\, C,\, S$	$\pi ^+ \pi ^- : \mathcal{B}$	$K^0 \bar{K}^0 : \mathcal{B}$	$K^+ \pi ^- : \mathcal{B},\, C$
$K^+ \pi ^- : \mathcal{B},\, C$	$K^+ {\eta } : \mathcal{B},\, C$	$K^+ {\eta '} : \mathcal{B},\, C$			$K^+ K^- : \mathcal{B}$	
$K^+ \pi ^0 : \mathcal{B},\, C$					$K^+ K^0 : \mathcal{B},\, C$	
$$K^0 \pi ^+ : \mathcal{B},\, C$$

**Table 2:** Observables of $B\rightarrow PV$ decays mediated by $b\rightarrow s$ transitions that are used in the fit

$$\varvec{b \rightarrow s}$$					
$B \rightarrow K^* \pi$	$B \rightarrow K \rho$	$B \rightarrow K^* \eta$	$B \rightarrow K^* \eta '$	$B \rightarrow K \phi$ [25–28]	$B \rightarrow K \omega$ [29, 30]
$K^{*0} \pi ^0 : \mathcal{B},\, C$	$K^{0} \rho ^0 : \mathcal{B},\, C,\, S$	$K^{*0} {\eta } : \mathcal{B},\, C$	$K^{*0} {\eta '} : \mathcal{B}, \, C$	$K^{0} \phi : \mathcal{B},\, C,\, S$	$K^{0} \omega : \mathcal{B},\, C,\, S$
$K^{*+} \pi ^- : \mathcal{B},\, C$	$K^{+} \rho ^- : \mathcal{B},\, C$	$K^{*+} {\eta } : \mathcal{B},\, C$	$K^{*+} {\eta '} : \mathcal{B},\, C$	$K^{+} \phi : \mathcal{B},\, C$	$K^{+} \omega : \mathcal{B},\, C$
$K^{*+} \pi ^0 : \mathcal{B},\, C$	$K^{+} \rho ^0 : \mathcal{B},\, C$				
$K^{*0} \pi ^+ : \mathcal{B},\, C$	$K^{0} \rho ^+ : \mathcal{B},\, C$

**Table 3:** Observables of $B\rightarrow VV$ decays mediated by $b\rightarrow s$ and $b \rightarrow d$ transitions that are used in the fit

$\varvec{b \rightarrow s}$					$\varvec{b \rightarrow d}$	
$B \rightarrow K^* \rho$	$B \rightarrow K^* \phi$ [36–38]	$B \rightarrow K^* \omega$	$B_s \rightarrow \phi \phi$	$B_s \rightarrow K^* K^*$ [39]	$B_s \rightarrow K^* \phi$	$B \rightarrow K^* K^*$
$K^{*0} \rho ^0 : \mathcal{B},\, C,\, f_L$	$K^{*0} \phi : \mathcal{B},\, C,\, C_{L, \perp },$	$K^{*0} \omega : \mathcal{B},\, C,\, f_L$	$\phi \phi : \mathcal{B},\, f_L$	$K^{*0} K^{*0} : \mathcal{B},\, f_L$		$K^{*0} K^{*0} : \mathcal{B},\, f_L$
$K^{*+} \rho ^- : \mathcal{B},\, C,\, f_L$	$f_{L, \perp },\, \phi _{\parallel , \perp }$	$K^{*+} \omega : \mathcal{B},\, C,\, f_L$			$K^{*+} \phi : \mathcal{B},\, f_L$	$K^{*+} K^{*0} : \mathcal{B},\, f_L$
$K^{*+} \rho ^0 : \mathcal{B},\, C,\, f_L$	$K^{*+} \phi : \mathcal{B},\, C,\, C_{L, \perp },$					
$K^{*0} \rho ^+ : \mathcal{B},\, C,\, f_L$	$f_{L, \perp },\, \phi _{\parallel , \perp }$					

In addition we investigate the complementarity of composed observables, the ratios $R_{c,n}^{B, M_a, M_b}$ (2.15) of branching fractions and differences of CP asymmetries ${\varDelta } C$ (2.16). In the future, it is desirable to have direct experimental determinations of the uncertainties for these “composed” observables that already account for the cancelation of common experimental systematic uncertainties, which are only accessible to the experimental collaborations themselves. This is important, since usually outsiders are not in the position to account retroactively for cancelations of systematic errors and are restricted to the application of rules of error propagation to the uncertainties of the measurements of the involved components, which then might result in too conservative estimates. Of course such a procedure on the experimental side requires that the according decay modes with charged and neutral initial/final states can be analyzed simultaneously, which is the case for Babar, Belle, and also Belle II. In this context it should be noted that ratios of gaussian distributed quantities are not gaussian distributed, although the differences are small as long as the tail regions of the distribution do not contribute. The details of the treatment of Gaussian and ratio of Gaussian distributed experimental probability distributions of the measurements are given in Appendix B.

The Tables 1, 2, and 3 show that the decay systems $B\rightarrow K\pi ,\, K^*\pi ,\, K\rho ,\, K^*\rho$ (and $K^*\phi$) are the ones with the most measured observables, allowing to investigate the complementarity of the constraints imposed on the phenomenological parameter of WA by branching fractions versus CP asymmetries versus other observables in *VV* final states. In these cases we can also form the ratios of branching fractions Eq. (2.15) and differences of CP asymmetries Eq. (2.16). We will perform fits using two different sets of observables for these systems. In the first, called “Set I”, we will use four branching fractions and four direct CP asymmetries. In the lack of precise experimental data on the mixing-induced CP asymmetry *S*, we rather prefer to predict them from the results of the fit then including them in the fit; see Appendix B.4 for details on the procedure. Such predictions can be tested with measurements of *S* by Belle II and LHCb in the near future [40–42] and are given for the SM and some NP fits in Tables 7 and 10. The second “Set II” contains the fully independent observables of one branching fraction, three ratios $R_{c,n}^{B,M_a,M_b}$, three direct CP asymmetries *C* and the difference of CP asymmetries ${\varDelta } C$—see Table 5 for the explicit list of observables. In summary:

2.19 $$\begin{aligned} \text{ Set } \text{ I }&: (4 \times \mathcal{B}) + (4 \times C) \nonumber \\ \text{ Set } \text{ II }&: (1 \times \mathcal{B}) + (3 \times R_{c,n}) + (3 \times C) + {\varDelta } C . \end{aligned}$$

## $B \rightarrow MM$ in QCD factorization

Here we revisit the building blocks that arise in QCDF to calculate the final-state-dependent corrections needed for a suitable prediction of the decay amplitudes $\lambda _f$ Eq. (2.8) and details of their treatment in our analysis. Most importantly, the parametrization of the endpoint divergences arising in weak-annihilation (WA) and hard-scattering (HS) contributions are given, which will be determined from experimental data in Sects. 4 and 5 in the framework of the SM and scenarios of NP, respectively. Further, we describe in Sect. 3.2 the determination of the relative magnitude of WA amplitudes compared to the leading ones in the SM and NP scenarios, as they are formally of subleading order in $1/m_b$, but chirally enhanced.

In our analysis all decay modes are driven by the same flavor transition $b \rightarrow D$ ($D = d, s$), which is described by the effective Hamiltonian of electroweak interactions. In the SM [2, 3]

3.1 $$\begin{aligned} \mathcal{H}_\mathrm{eff}^D&= \frac{G_F}{\sqrt{2}} \sum _{p=u,c} \lambda _p^{(D)} \left( C_1 O_1^p + C_2 O_2^p \right. \nonumber \\&\quad +\left. \sum _{i=3}^{10} C_i O_i + C_{7\gamma } O_{7\gamma } + C_{8g} O_{8g} \right) + \text{ h.c. }, \end{aligned}$$

where $G_F$ denotes the Fermi constant and $\lambda _p^{(D)} \equiv V_{pb}^{} V_{pD}^*$ are products of elements of the CKM matrix. The flavor-changing operators are

3.2 $$\begin{aligned} O_1^p&= (\bar{D}p)_{V-A} (\bar{p}b)_{V-A}, \nonumber \\ O_2^p&= (\bar{D}_{\alpha }p_{\beta })_{V-A} (\bar{p}_{\beta }b_{\alpha })_{V-A}, \nonumber \\ O_{3(5)}&= (\bar{D}b)_{V-A}\sum _{q}(\bar{q}q)_{V \mp A}, \nonumber \\ O_{4(6)}&= (\bar{D}_\alpha b_{\beta })_{V-A}\sum _{q}(\bar{q}_{\beta }q_{\alpha })_{V \mp A}, \nonumber \\ O_{7(9)}&= \frac{3}{2}(\bar{D}b)_{V-A}\sum _{q}e_q(\bar{q}q)_{V\pm A}, \nonumber \\ O_{8(10)}&= \frac{3}{2}(\bar{D}_{\alpha }b_{\beta })_{V-A}\sum _{q}e_q(\bar{q}_{\beta }q_{\alpha })_{V \pm A}, \nonumber \\ O_{7\gamma }&= -\frac{e\, m_b}{8\pi ^2}\, (\bar{D}\,\sigma ^{\mu \nu }(1+\gamma _5)\, b) F_{\mu \nu } , \nonumber \\ O_{8g}&= -\frac{g_s m_b}{8\pi ^2} (\bar{D}_\alpha \sigma ^{\mu \nu }(1+\gamma _5) T^a_{\alpha \beta } b_\beta ) G_{\mu \nu }^a, \end{aligned}$$

where $(\bar{q}_1 q_2)_{V\pm A}=\bar{q}_1\gamma _\mu (1\pm \gamma _5)q_2$, the sum is over active quarks $q = (u,d,s,c,b)$, with $e_q$ denoting their electric charge in fractions of |*e*| and $\alpha ,\beta$ denoting color indices. The Wilson coefficients, $C_i$, are obtained by a matching calculation at a scale typically of the order $\mathcal {O}(M_W)$ of the *W*-boson mass and are evolved down to the low energy scale of $\mathcal {O}(m_b)$ of the bottom-quark mass by means of the renormalization group (RG) equation. Here, we will use the modified counting scheme, as described in [3], in which the dominant part of the electroweak penguin Wilson coefficients $C_i$ ($i = 7,\ldots ,10$) are treated as a leading order effect. The electroweak penguin operators belong to the isospin-violating part of $\mathcal{H}_\mathrm{eff}^D$ and affect in principle isospin-asymmetric observables, as for example Eqs. (2.15) and (2.16).

### Weak annihilation in QCDF

As was established by Beneke et al. [2, 3], the matrix elements of the involved operators can be treated systematically in a $1/m_b$ expansion that has become known as QCD factorization (QCDF). At leading order this yields

3.3 $$\begin{aligned}&\langle M_1 M_2 | O_i | B \rangle = \sum _j F_j^{B\rightarrow M_1}(m_2^2) \int _0^1 \mathrm{d}u \, T^\mathrm{I}_{ij}(u) {\varPhi }_{M_2}(u)\nonumber \\&\quad + (M_1 \leftrightarrow M_2 ) + \!\!\int _0^1 \mathrm{d}\xi \mathrm{d}v \mathrm{d}u \, T^\mathrm{II}_{i}(u,v,\xi ) {\varPhi }_{B}(\xi ) {\varPhi }_{M_1}(v) {\varPhi }_{M_2}(u) \nonumber \\&\quad + \mathcal {O}\left( \frac{{\varLambda }_\mathrm{QCD}}{m_b}\right) \end{aligned}$$

two terms with hard-scattering kernels $T^\mathrm{I,II}$, which are calculable in perturbation theory to higher orders in the QCD coupling $\alpha _s$. They are convoluted with light-cone distribution amplitudes (DA) of the light mesons, denoted as ${\varPhi }_{M_i}$, and are multiplied by the corresponding heavy-to-light form factors $F_j^{B\rightarrow M_i}$ in the case of $T^\mathrm{I}$ and involve an additional convolution with the *B*-meson distribution amplitude ${\varPhi }_B$ in the case of $T^\mathrm{II}$. In Eq. (3.3), the meson $M_1$ inherits the spectator quark of the decaying *B* meson, and depending on the final state, the decay amplitude might depend also on matrix elements with $M_1 \leftrightarrow M_2$; see [3, 5] for details.

At leading order in $1/m_b$, the perturbative kernels $T^\mathrm{I,II}$ have been calculated up to NLO in strong coupling $\alpha _s$ [2, 3] and throughout we will stay within this approximation. Contrary to previous works [3, 5, 15], we employ Wilson coefficients of the weak Hamiltonian evaluated at the scale $m_b$ even in WA and HS contributions, only the strong coupling $\alpha _s$ is evaluated at the semi-hard scale $\mu _h = \sqrt{{\varLambda }_\mathrm{QCD} m_b}$. In the SCET approach this is apparent as a subsequent matching step from QCD to SCET${}_\mathrm{I}$ taken at $\mu \sim m_b$ such that the Wilson coefficients of the weak Hamiltonian do not run below $m_b$, whereas $\alpha _s$ does. Equivalent arguments in the framework of QCDF can be found in [43].

The NNLO $\alpha _s$ corrections to $T^\mathrm{I,II}$ are work in progress and by now the only lacking part are corrections to $T^\mathrm{I}$ from QCD- and QED-penguin operators $i =3,\dots , 10$ as well as the dipole operators $i = 7\gamma , 8g$. These NNLO corrections are especially important for decays under consideration here because strong phases are generated in QCDF only at NLO and higher order corrections might be large, apart from the reduction of renormalization scheme dependences. In the case of the color-allowed and color-suppressed current-current contributions due to $O^p_{1,2}$, the NNLO contributions to $T^\mathrm{I}$ cancel in large parts for both, real and imaginary parts, [44–46] with the ones to $T^\mathrm{II}$ [47–49] in the corresponding amplitudes $\alpha _{1,2}(MM)$ [46, 50] for $MM = \pi \pi ,\, \rho \pi$, leaving them close to NLO predictions. This might not be the case for final states considered here.

As it is discussed in detail in the literature [3, 5], contributions from HS and WA topologies, which are subleading in $1/m_b$, elude so far from a systematic treatment in QCDF. However, they can be chirally enhanced and contribute sizable corrections in predictions. Due to the ignorance of the respective QCD mechanisms, additional phenomenological parameters were introduced

3.4 $$\begin{aligned} X_k&= \left( 1 + \rho _k\right) \ln \frac{m_b}{{\varLambda }_\mathrm{QCD}} ,\nonumber \\ \rho _k&\equiv |\rho _k| e^{i\phi _k} \end{aligned}$$

with the complex parameters $\rho _k$ for $k = A, H$.

In the HS they originate from terms involving twist-3 light-cone DAs ${\varPhi }_{m1}(y)$ with ${\varPhi }_{m1}(y) \ne 0$ for $y\rightarrow 1$ in convolutions

3.5 $$\begin{aligned} \int _0^1 \frac{{\varPhi }_{m1}(y) \text {d}y}{1 - y}&\equiv {\varPhi }_{m1}(1) X_H + \int _0^1 \frac{{\varPhi }_{m1}(y) \text {d}y}{[1 - y]_+} , \end{aligned}$$

which are regulated by the introduction of the phenomenological parameter $X_H$,^2^ representing a soft-gluon interaction with the spectator quark. As indicated above, it is expected that $X_H \sim \ln (m_b/{\varLambda }_\mathrm{QCD})$ because it arises in a perturbative calculation of these soft interactions that are regulated in principle latest by a physical scale of order ${\varLambda }_\mathrm{QCD}$. Neither the adequate degrees of freedom nor their interactions, which should be used in an effective theory below this scale are known. It is also conceivable that factorization might be achieved at some intermediate scale between $m_b$ and ${\varLambda }_\mathrm{QCD}$. The factor $(1 + \rho _H)$ summarizes the remainder of an unknown nonperturbative matrix element, including the possibility of a strong phase, which affects especially the predictions of CP asymmetries. The numerical size of the complex parameter $\rho _H$ is unknown, however, too large values will give rise to numerically enhanced subleading $1/m_b$ contributions compared to the formally leading terms putting to question the validity of the $1/m_b$ expansion of QCDF.

WA is entirely subleading in $1/m_b$ and consists in principle of six different building blocks $A_{k}^{i,f}$ ($k = 1,2,3$), which are characterized by gluon emission from the initial (*i*) and final (*f*) states and the three possible Dirac structures that are involved: $k = 1$ for $(V-A)\otimes (V-A)$, $k = 2$ for $(V-A)\otimes (V+A)$, and $k = 3$ for $(-2)(S-P)\otimes (S+P)$. They contribute to non-singlet annihilation amplitudes with specific combinations of Wilson coefficients of the 4-quark operators [3, 5]

3.6 $$\begin{aligned} b_1&= \frac{C_F}{N_c^2} C_1 A_1^i , \nonumber \\ b_2&= \frac{C_F}{N_c^2} C_2 A_1^i , \nonumber \\ b_3^p&= \frac{C_F}{N_c^2} \left[ C_3 A_1^i + C_5 (A_3^i + A_3^f) + N_c C_6 A_3^f \right] , \nonumber \\ b_4^p&= \frac{C_F}{N_c^2} \left[ C_4 A_1^i + C_6 A_2^i \right] , \nonumber \\ b_{3, \mathrm{EW}}^p&= \frac{C_F}{N_c^2} \left[ C_9 A_1^i + C_7 (A_3^i + A_3^f) + N_c C_8 A_3^f \right] , \nonumber \\ b_{4, \mathrm{EW}}^p&= \frac{C_F}{N_c^2} \left[ C_{10} A_1^i + C_8 A_2^i \right] , \end{aligned}$$

and depend on $M_1$ and $M_2$. Here, $N_c = 3$ denotes the number of colors and the color factor $C_F = 4/3$. In particular, they correspond to the amplitudes due to current-current ($b_1,\, b_2$), QCD-penguin ($b_3^p,\, b_4^p$) and electroweak penguin ($b_{3, \mathrm{EW}}^p,\, b_{4, \mathrm{EW}}^p$) annihilation. Below we will frequently refer to WA amplitudes with the normalization [5]

3.7 $$\begin{aligned} \beta _i^{(p)}&= \mathcal{N}_\beta \, \left\{ \begin{array}{cl} \displaystyle \frac{b_i^{(p)}}{m_{M_2}} &{} \quad \text{ for } M_1 M_2 = V^\pm V^\pm \\ \displaystyle \frac{b_i^{(p)}}{m_{B}} &{} \quad \text{ for } \text{ all } \text{ others } \end{array}\right. \end{aligned}$$

where the argument $M_1 M_2$ has been suppressed and $\mathcal{N}_\beta \equiv f_B f_{M_1}/(m_B F^{B\rightarrow M_1})$ is independent on $M_2$. As in the case of HS, the endpoint singularities in WA amplitudes are regulated in a model-dependent fashion. The results are expressed in terms of convolutions of hard-scattering kernels with DAs of twist-2 and chirally enhanced twist-3, involving phenomenological parameters $X_A$

3.8 $$\begin{aligned} \int _0^1 \frac{\text {d}y}{y}&\rightarrow X_A ,\nonumber \\ \int _0^1 \frac{\ln y \, \text {d}y }{y}&\rightarrow -\frac{1}{2} (X_A)^2 , \end{aligned}$$

which in principle are different for each meson and each building block $A_k^{i,f}$. Explicit expressions for $A_k^{i,f}$ in terms of $X_A$ are given for $MM = PP,\, PV,\, VP,\, VV$ in the literature [5, 23, 51, 52], but independently one has $A^f_{1,2} = 0$. As a further simplification, it is assumed in the literature that there is only one phenomenological parameter, independent of meson type and Dirac structure, such that $A^{i,f}_k(X_A)$ are functions of the same parameter. In this context we would like to note that in the most relevant WA amplitude $\beta _3^c$ the building block $A^f_3$ is parametrically enhanced by $N_c$ and in the SM a large Wilson coefficient. In consequence its contribution dominates over the ones of $A^i_{1,3}$. It should be noted that the WA amplitudes (3.6) in the light-cone sum rule (LCSR) approach exhibit the same dependence on the products of Wilson coefficients and building blocks [53], however, in this approach the calculation of $A^{i,f}_k$ does not suffer from endpoint singularities due to different assumptions and approximations. With the latter in mind, a more general approach would be to interpret the building blocks themselves as phenomenological parameters, or equivalently introduce one $X_A$ for each of them. When investigating new-physics effects, it is desirable to keep the explicit dependence on the Wilson coefficients in (3.6) since they depend on NP parameters, including new weak phases. In the case of non-negligible WA contributions, the CP asymmetries and branching fractions will be sensitive to the interference of the new-physics phases and the strong phases from $X_A$.

As already indicated, the phenomenological parameters $X_{A,H}$ are unknown and their size is conventionally adjusted within some range $|\rho _{A,H}| \lesssim 2$ to reproduce data whereas the phase $\phi _{A,H}$ is kept arbitrary and varied freely to estimate the uncertainty in theoretical predictions of observables within QCDF due to WA and HS. This procedure showed the phenomenological importance of WA and constitutes a major source of theoretical uncertainty in predictions within the SM [5] and searches beyond [15] and below we will refer to it as “conventional QCDF”.

In this work, we are going to fit $\rho _A$—and for $B\rightarrow K \pi$ also $\rho _H$—from data. As a consequence, no predictions will be possible for those observables that are used in the fit, while the fitted values of $\rho _A$ depend on the short-distance model under consideration. Yet, the consistency of the underlying short-distance model can be tested. We perform our fits in the framework of the SM, and further in new-physics scenarios simultaneously with the additional NP parameters. In the latter case, the determination of the NP parameters will take into account the uncertainty of the WA contribution when marginalizing over $\rho _A$.

This procedure is different from conventional QCDF in as much as it assumes one universal parameter $\rho _A$ for all observables in one specific decay mode. Indeed, in conventional QCDF the independent variation of $\rho _{A,H}$ for each observable in a specific decay corresponds to a different WA (and HS) parameter for each observable. However, since in QCDF the parameters $\rho _{A,H}$ are introduced at the level of decay amplitudes one would expect that they are the same for all observables of a specific decay mode. Consequently, conventional QCDF allows for situations where experimental measurements and theory predictions for two observables are in agreement, although for the first observable the agreement is reached for values of $\phi _{A,H}$ that might be very different from those where the agreement is reached for the second observable.

In the lack of precise data for most of the decays, we make the further assumption of a WA parameter that is even universal for decay modes that are related by the exchange of $(u \leftrightarrow d)$ quarks. As an example, this allows one to combine observables of the four decay channels $\bar{B}^0\rightarrow \bar{K}^0 \pi ^0,\, K^-\pi ^+$, and $B^-\rightarrow K^-\pi ^0,\, \bar{K}^0\pi ^-$, to which we refer as “decay system” $B\rightarrow K\pi$. All considered decay systems and the according observables have been listed in Tables 1, 2, and 3. This assumption is motivated by the circumstance that the dominant contributions to the amplitude in all considered decays come actually from the linear combination $\hat{\alpha }_4(M_1 M_2) = \alpha _4(M_1 M_2) + \beta _3(M_1 M_2)$, which is due to isospin-conserving QCD-penguin operators $O_{3,\ldots ,6}$ (3.2). The definition of all $\alpha _i$’s can be found in [5], whereas $\beta _i$’s are given in Eq. (3.7). Other assumptions have been tested in the literature as for example universal weak annihilation among $B_s$ and $B_d$ decays into final states containing kaons and pions [54].

The procedure reflects the general idea inherent to $1/m_b$ expansions, which aim at a factorization into short-distance and universal nonperturbative quantities, where the latter are determined from data in the lack of first-principle determinations. Presently, however, factorization theorems are not yet established at subleading order that would support the existence of such universal quantities. While steps have been made to show that weak annihilation is factorizable and real [55], doubts are cast on the reality condition [56]. In view of this, our study can affirm at most experimental evidence against the assumption of one universal parameter per decay system. Therefore a positive affirmation may not be over interpreted. Finally, it must also be noted that contributions of not included NNLO corrections could be sizeable and in our fits they are interpreted as part of the phenomenological WA parameter.

### Size of power-suppressed corrections

In this work, we determine the size of subleading WA (and HS) contributions from data in the framework of the SM and NP scenarios. Due to the chiral enhancement, WA contributions are not necessarily $1/m_b$ suppressed numerically with respect to the leading order amplitudes. Therefore, it is of interest to know the relative magnitude of WA to leading amplitudes for the best-fit regions of $\rho _{A(H)}$. For this purpose we introduce the quantities

3.9 $$\begin{aligned} \xi _i^A(\rho _A)&= \left| \frac{\beta _i(\rho _A)}{\alpha _{(i +{\varDelta }_{i3}), \mathrm I}} \right| , \end{aligned}$$

for WA and

3.10 $$\begin{aligned} \xi _i^H(\rho _H)&= \left| \frac{\alpha _{i, \mathrm II}^\mathrm{tw-3}(\rho _H)}{\alpha _{i,\mathrm I} + \alpha _{i,\mathrm II}^\mathrm{tw-2}} \right| , \end{aligned}$$

for HS amplitudes. For the latter, $\alpha _{i, \mathrm II}^\mathrm{tw-3}$ denotes the subleading, but chirally enhanced, twist-3 contribution, whereas $\alpha _{i,\mathrm II}^\mathrm{tw-2}$ is the leading HS contribution from twist-2 DAs, which is free of endpoint divergences. The leading amplitudes are splitted into $\alpha _i = \alpha _{i, \mathrm I} + \alpha _{i, \mathrm II}$, with the two contributions from kernel I and II introduced in Eq. (3.3). Note that $\xi ^A_3 = |\beta _3/\alpha _{4, \mathrm I}|$. This definition is generalized for the case $MM = VV$, where $\xi ^A$ is defined as the mean value of the corresponding ratios for the longitudinal and negative polarized amplitudes $\xi _{i,VV}^A \equiv (\xi _{i,L}^A + \xi _{i,-}^A)/2$.

The most important contribution from power-suppressed corrections are clearly obtained from HS in $\alpha _2$, which is enhanced by the large Wilson coefficient $C_1$ and from the WA correction $\beta _3$ in QCD-penguin-dominated decays. Therefore $\xi _2^H$ and $\xi _3^A$ will play an important role in the phenomenological part of this work.

In the SM, the $\xi ^A$-ratios depend exclusively on $\rho _A$ and contour lines of constant $\xi ^A$ can easily be obtained in the complex $\rho _A$-plane. Concerning fits in new-physics scenarios, the $\xi ^A$-ratios depend in addition on new-physics parameters $\varvec{x}^\mathrm{NP}$, where dim($\varvec{x}^\mathrm{NP}$) corresponds to their number. The dependence is both, explicit in the Wilson coefficients and implicit on data via the likelihood. In this case one would be interested in the minimal value of $\xi ^A_i(\varvec{x}^\mathrm{NP})$ in the 68 % credibility regions (CR) of all NP parameters, but marginalized over $\rho _A$. Since the determination of this CRs requires huge computational efforts when dim$(\varvec{x}^\mathrm{NP}) > 2$, we proceed differently. In the course of the fit, we histogram in all two-dimensional subspaces of NP parameters $(x_a^\mathrm{NP},\, x_b^\mathrm{NP})$, with $a \ne b$, those values $\xi ^A$ in each bin $(x_a^\mathrm{NP},\, x_b^\mathrm{NP})$ that belong to the largest likelihood value when sampling the complementary subspace of the remaining NP parameters $x_c^\mathrm{NP}$ with $c\ne a$ and $c\ne b$. As a result, in each of the 2D-marginalized planes, “labeled” by (*a*, *b*), the 68 % CR will contain a smallest and a largest $\xi ^A_i$ in one of the bins in $(x_a^\mathrm{NP},\, x_b^\mathrm{NP})$, which all belong to the minimal $\chi ^2$ in the subspace of $x_c$. As the final range we choose the minimum of the smallest values and the maximum of the largest values from all the pairs (*a*, *b*) in our NP analyses in Sect. 5.

## Weak annihilation in the standard model

In this section we present the results of the determination of the WA parameter $\rho _A$ from data of various QCD-penguin- and WA- dominated nonleptonic charmless $B\rightarrow MM$ decays in the framework of the SM. This includes characteristics of the best-fit regions, the *p* values at the best-fit point and pull values for observables, as well as the relative amount of the subleading WA contribution needed to explain the data, which we quantify by the ratio $\xi _3^A$ defined in Eq. (3.9).

We start with an extensive discussion of the $B \rightarrow K\pi$ system, which shows the largest deviations from SM predictions for the difference of CP asymmetries ${\varDelta } C(K\pi )$ (see Eq. (2.16)), commonly known in the literature as the “${\varDelta } {\mathcal{A}_\mathrm{CP}}$ puzzle” and to a lesser extent in the ratio $R_n^B(K\pi )$. We investigate the “${\varDelta } {\mathcal{A}_\mathrm{CP}}$ puzzle” further in a simultaneous fit of the parameter of WA, $\rho _A$, and HS, $\rho _H$, and discuss the implications on other CP asymmetries in $B_{d,s}\rightarrow K\pi$.

We turn then to the discussion of the decays $B\rightarrow K\rho ,\, K^*\rho ,\, K^*\pi$, which allow also for studies of different sets of observables in Set I and Set II due to the rather numerous and quite precise measurements. Subsequently, we discuss briefly the results for other decays listed in Tables 1, 2, and 3 with some special comments on $B\rightarrow K \omega$ and $B\rightarrow K^*\phi$. For each decay system we present separate constraints from branching fractions, CP asymmetries, polarization fractions and relative phases on the WA parameter $\rho _A$, besides the combined ones.

Apart from the above listed penguin-dominated decays, we also study decays mediated solely by weak annihilation, such as $B \rightarrow K^+ K^-$ and $B_s \rightarrow \pi ^+ \pi ^-$. Being independent of $\beta _3$ and hence $A_3^f$, these decay modes are sensitive to a WA contribution from $A_{1,2}^i$ and provide access to different building blocks.

Based on our previous fit results, we discuss finally the assumption of a universal WA for $B_d$ and $B_s$ decays into the same final state and investigate in particular consequences for CP asymmetries in $B_s \rightarrow K\pi$ in view of the “${\varDelta } {\mathcal{A}_\mathrm{CP}}$ puzzle” in $B\rightarrow K\pi$.

The statistical procedure used in all fits is described in Appendix B. In the SM, we deal mostly with the fit of one complex-valued parameter $\rho _A$ except for the $B\rightarrow K \pi$ system, where we also perform a simultaneous fit of $\rho _A$ and $\rho _H$. When fitted, for both parameters a uniform prior

4.1 $$\begin{aligned} 0 \le |\rho _{A,H}| \le 8, \quad 0 \le \phi _{A,H} \le 2\pi , \end{aligned}$$

is assumed and no restriction is imposed on the phases. In comparison, in conventional QCDF the magnitude $|\rho _{A,H}| \le 2$ is used for uncertainty estimates of theoretical predictions. In the case that $\rho _H$ is not fitted, but treated as a nuisance parameter instead, we use $|\rho _H| = 1$ and vary $0 \le \phi _H \le 2 \pi$.

Our findings for lower and upper bounds on $\rho _A$ in the 68 % CR are summarized in Table 4 for all considered decay systems. It can be seen that data requires nonzero values of $|\rho _A|$ to be in agreement with QCDF predictions in the SM. In some cases they are much larger compared to the conventionally adapted ranges, allowing thus in principle for a better agreement of theoretical predictions with data. Since we use Wilson coefficients at the scale $\mu \sim m_b$ in WA (and HS) contributions, contrary to [5, 15], our numerical values of $|\rho _A|$ are in general a bit larger compared to the ones known in the literature. Representing the size of a nonperturbative quantity, $|\rho _A|$ is expected naively to be of order one, whereas too large values would put in doubt the convergence of the $1/m_b$ expansion.

**Table 4:** Compilation of the power-suppressed ratio $\xi _3^A$ at the best-fit point (BFP) and in the 68 and $95~\%$ CRs, as well as lower and upper bounds on the fit parameter $|\rho _A|$ in the 68 % CR for all relevant decay systems. For $B \rightarrow (K\pi ,\, K^*\pi ,\, K\rho ,\, K^*\rho )$, values correspond to the fit with the observable Set II. The pure WA decay $B^0 \rightarrow K^+ K^-$ is not included in the decay system $B \rightarrow KK$ and $\rho _A$-bounds are given separately in parentheses

	$B \rightarrow K \pi$	$B \rightarrow K \eta$	$B \rightarrow K \eta '$	$B \rightarrow K K$	$B_s \rightarrow K K$	$B_s \rightarrow \pi \pi$	$B_s \rightarrow K \pi$
$$MM = PP$$							
$$\xi _3^A$$							
BFP	0.39	0.08	1.83	0.58	1.83	–	0.96
$68~\%$ CR	$[0.37;\, 0.54]$	$[0.00;\, -]$	$[0.18;\,3.25]$	$[0.00;\,2.07]$	$[0.02;\,2.09]$	–	$[0.56;\,1.54]$
$95~\%$ CR	$[0.34;\, 0.69]$	$[0.00;\, -]$	$[0.16;\, 3.34]$	$[0.00;\, 2.10]$	$[0.00;\, 2.13]$	–	$[0.44;\, 1.83]$
$$|\rho _A|$$							
Lower	$>$1.8	$>$0	$>$0.9	$>$0 (0.9)	$>$0	$>$3.4	$>$2.3
Upper	$<$3.9	–	$<$7.7	$<$6.1 (8.6)	$<$5.5	$<$10.9	$<$4.8

	$B \rightarrow K^* \pi$	$B \rightarrow K \rho$	$B \rightarrow K^* \eta$	$B \rightarrow K^* \eta '$	$B \rightarrow K \phi$	$B \rightarrow K \omega$	
$$MM = PV$$							
$$\xi _3^A$$							
BFP	0.89	0.78	2.74	0.48	0.50	2.7	
$68~\%$ CR	$[0.75;\, 1.40]$	$[0.39;\, 1.55]$	$[0.71;\, 3.77]$	$[0.02;\, 7.84]$	$[0.40;\, 2.41 ]$	$[0.63;\, 2.88]$	
$95~\%$ CR	$[0.69;\, 1.56]$	$[0.16;\, 2.18]$	$[0.64;\, 5.06]$	$[0.02;\, 8.41]$	$[0.32;\, 2.54]$	$[0.57;\, 2.88]$	
$$|\rho _A|$$							
Lower	$>$1.4	$>$0.8	$>$1.1	$>$0	$>$0.8	$>$1.3	
Upper	$<$3.4	$<$3.4	$<$4.4	$<$6.1	$<$3.6	$<$4.3	

	$B \rightarrow K^* \rho$	$B \rightarrow K^* \phi$	$B_s \rightarrow K^* \phi$	$B \rightarrow K^* K^*$	$B_s \rightarrow K^* K^*$	$B_s \rightarrow \phi \phi$	$B \rightarrow K^* \omega$
$$MM = VV$$							
$$\xi _3^A$$							
BFP	1.33	0.38	1.53	1.84	3.01	0.50	0.91
$68~\%$ CR	$[0.84;\, 1.94]$	$[0.31;\, 0.43]$	$[0.30;\, 2.05]$	$[0.85;\, 2.68]$	$[1.94;\, 3.79]$	$[0.49;\, 1.11]$	$[0.20;\, 1.39]$
$95~\%$ CR	$[0.56;\, 2.33]$	$[0.25;\, 0.50]$	$[0.10;\, 2.15]$	$[0.09;\, 2.90]$	$[0.96;\, 4.17]$	$[0.41;\, 1.38]$	$[0.09;\, 1.46]$
$$|\rho _A|$$							
Lower	$>$1.0	$>$0.6	$>$0.3	$>$1.2	$>$1.6	$>$0.7	$>$0.3
Upper	$<$2.9	$<$1.8	$<$3.2	$<$3.0	$<$3.6	$<$2.3	$<$2.4

Further we list the ratio $\xi _3^A$ of WA amplitudes to leading ones as a measure of the numerical relevance of these formally subleading but chirality enhanced contributions. While large subleading WA amplitudes do not necessarily imply a breakdown of the $1/m_b$ expansion, it is generally assumed that the factorization achieved at leading order proves useful only if subleading WA amplitudes are not be too large. Using the priors for our analysis which result in a wide range for $\xi ^A_3$, we can explicitly show that data does not require huge WA. At the best-fit point of $\rho _A$ the according value is indeed $\xi _3^A < 1$ for many decay systems. Although at the best-fit point $\xi _3^A$ might reach values up to 2 or even 3 for some decay systems, once considering the 68 % CR in $\rho _A$, it is possible to have again $\xi _3^A < 1$ (except for $B_s \rightarrow K^*K^*$) for the price of some tension among data and prediction. Bearing in mind the chirality enhancement, our fits of the data thus do not indicate anomalously huge WA contributions, which put the usefulness of the $1/m_b$ expansion into question in principle. By definition, there is no $\xi _3^A$ for the two pure WA modes $B_d \rightarrow K^+K^-$ and $B_s \rightarrow \pi ^+\pi ^-$.

### Results for $B \rightarrow K \pi$

In this section we discuss first fits of only $\rho _A$ from $B\rightarrow K\pi$ observables encountering thereby the well-known “${\varDelta } {\mathcal{A}_\mathrm{CP}}$ puzzle”. In the SM it can be explained only reasonably well in the presence of large color-suppressed current-current tree contributions that are strongly dependent on $\rho _H$. Therefore we perform in a second step a simultaneous fit of $\rho _A$ and $\rho _H$ to a modified set of $B\rightarrow K\pi$ observables, and discuss the possibility to discern large color-suppressed tree contributions in future measurements of CP asymmetries with $K^0\pi ^0$ final states. It should be emphasized that in general both, $\rho _A$ and $\rho _H$ should be considered in fits due to their different contributions to CP-averaged and asymmetric observables. Hence simultaneous fits of $\rho _A$ and $\rho _H$ are in principle of greater interest, but apart from $B\rightarrow K\pi$ data, the current precision of other decay system’s data does not yet permit this kind of analysis.

**Fig. 1:**
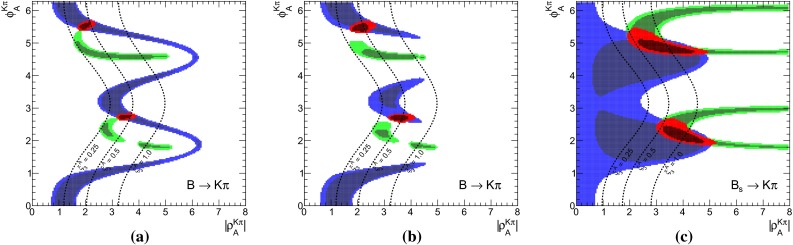
The 68 % (*dark*) and 95 % (*bright*) CRs of $\rho _A^{M_1 M_2}$ from a fit of observables in the ($B \rightarrow K \pi$)-system in **a** Set I and **b** Set II, and for comparison in the **c** ($B_s \rightarrow K \pi$)-system. Allowed regions are shown for $\mathcal{B}$ and $R_{n,c}$ (*blue*), *C*, and ${\varDelta } C$ (*green*) and their combination (*red*). The *dashed lines* correspond to constant $\xi ^A_3 = (0.25, 0.5, 1.0)$ from left to right

The $B\rightarrow K \pi$ system offers the most precise measured branching fractions and CP asymmetries (see Table 1) among the decays considered here. In consequence, we find stringent bounds on the WA parameter $\rho _A^{K\pi }$. This can be seen in Fig. 1a when using the observables in Set I and Fig. 1b for Set II. The allowed regions from both, $\mathcal{B}$ and/or $R_{c,n}^{B,M_a,M_b}$ (blue) as well as *C* and/or ${\varDelta } C$ (green) are very distinct leaving two tiny overlap regions (red) at 68 % probability around $\rho _A^{K\pi } \approx 2.1 \exp (i \, 5.5)$ and $\rho _A^{K\pi }\approx 3.4 \exp (i\, 2.7)$, which differ only slightly for both Sets I and II. The best-fit points listed in Table 5 fall into the solution with larger $|\rho _A^{K\pi }|\approx 3.4$, but it must be noted that the other solution provides almost equally good fits in terms of $\chi ^2$.

**Table 5:** Compilation of *p* values and pulls of the SM fit, evaluated at the best-fit point of $\rho _A^{M_a M_b}$ for the two different sets of observables Set I and Set II for the decays $B \rightarrow K\pi ,\, K^*\pi ,\, K\rho ,\, K^*\rho$

$M_a M_b$	$K \pi$		$K^* \pi$		$K \rho$		$K^* \rho$	
Set	SI	SII	SI	SII	SI	SII	SI	SII
*p* value	0.44	0.04	0.95	0.90	1	1	1	0.97
Best-fit point	3.39; 2.73	3.34; 2.71	1.79; 5.85	1.61; 5.84	2.57; 2.79	2.69; 2.68	2.31; 2.74	1.56; 5.66
$\mathcal{B}(\bar{B}^0 \rightarrow M_a^0 M_b^0)$	$+0.3 \, \sigma$	–	$-0.3 \, \sigma$	–	$0.0 \, \sigma$	–	$0.0 \, \sigma$	–
$\mathcal{B}(\bar{B}^0 \rightarrow M_a^- M_b^+)$	$0.0 \, \sigma$	–	$0.0 \, \sigma$	–	$0.0 \, \sigma$	–	$+0.3 \, \sigma$	$+0.1 \, \sigma$
$\mathcal{B}( B^- \rightarrow M_a^- M_b^0)$	$0.0 \, \sigma$	–	$+0.6 \, \sigma$	–	$0.0 \, \sigma$	–	$0.0 \, \sigma$	–
$\mathcal{B}( B^- \rightarrow M_a^0 M_b^-)$	$0.0 \, \sigma$	$+0.2 \, \sigma$	$0.0 \, \sigma$	$+0.1 \, \sigma$	$0.0 \, \sigma$	$+0.1 \,\sigma$	$0.0 \, \sigma$	–
$R^B_c$	–	–	–	–	–	–	–	$-0.5 \, \sigma$
$R^B_n$	–	$\mathbf {-1.9 \, \sigma }$	–	$+0.6 \, \sigma$	–	$0.0 \, \sigma$	–	$+0.6 \, \sigma$
$R^{M_a}_c$	–	$0.0 \, \sigma$	–	$+0.8 \, \sigma$	–	$+0.7 \, \sigma$	–	$-0.8 \, \sigma$
$R^{M_b}_c$	–	$+0.9 \, \sigma$	–	$0.0\, \sigma$	–	$-0.2 \, \sigma$	–	–
$C(\bar{B}^0 \rightarrow M_a^0 M_b^0)$	$0.0 \, \sigma$	$0.0 \, \sigma$	$+0.5 \, \sigma$	$+0.4 \, \sigma$	$0.0 \, \sigma$	$0.0 \, \sigma$	$0.0 \, \sigma$	$0.0 \, \sigma$
$C(\bar{B}^0 \rightarrow M_a^- M_b^+)$	$+0.7 \, \sigma$	$+0.1 \, \sigma$	$+0.1 \, \sigma$	$+0.1\, \sigma$	$0.0 \, \sigma$	$+0.1 \, \sigma$	$+0.5 \, \sigma$	$+0.6 \, \sigma$
$C( B^- \rightarrow M_a^- M_b^0)$	$\mathbf {-2.1 \, \sigma }$	–	$0.0 \, \sigma$	–	$0.0 \, \sigma$	–	$+0.3 \, \sigma$	–
$C( B^- \rightarrow M_a^0 M_b^-)$	$\mathbf {+1.0 \, \sigma }$	$\mathbf {+1.0 \, \sigma }$	$+0.9 \, \sigma$	$\mathbf {+1.0\,\sigma }$	$+0.7 \, \sigma$	$+0.7 \, \sigma$	$+0.1 \, \sigma$	$0.0 \, \sigma$
${\varDelta } C$	–	$\mathbf {-2.8 \, \sigma }$	–	$-0.1 \, \sigma$	–	$0.0 \, \sigma$	–	$0.0 \, \sigma$
$f_L(\bar{B}^0 \rightarrow M_a^0 M_b^0)$	–	–	–	–	–	–	$0.0 \, \sigma$	$0.0 \, \sigma$
$f_L(\bar{B}^0 \rightarrow M_a^- M_b^+)$	–	–	–	–	–	–	$-0.6 \, \sigma$	$-0.5 \, \sigma$
$f_L( B^- \rightarrow M_a^- M_b^0)$	–	–	–	–	–	–	$+0.7 \, \sigma$	$+0.9 \, \sigma$
$f_L( B^- \rightarrow M_a^0 M_b^-)$	–	–	–	–	–	–	$0.0 \, \sigma$	$0.0 \, \sigma$

Large pull values are in bold

As shown in Table 5, the *p* values at the best-fit points of the fits of Set I and Set II are very different: 0.44 versus 0.04, respectively. The reason are large pull values of composed observables in Set II at the best-fit point: $-2.8\sigma$ for ${\varDelta } C(K\pi )$ and $-1.9\sigma$ for $R_n^B(K\pi )$, compared to Set I: $-2.1\sigma$ for $C(B^- \rightarrow K^- \pi ^0)$, showing the importance and complementarity of composed observables. The large pull values in CP asymmetries arise from the higher statistical weight of the more precisely measured branching fractions in their combined fit, reflecting the “${\varDelta } {\mathcal{A}_\mathrm{CP}}$ puzzle” in the $B\rightarrow K \pi$ sytem. The individual pull values of $C(B^- \rightarrow K^- \pi ^0)$ and $C(\bar{B}^0 \rightarrow K^- \pi ^+)$ in Set I add up to the large pull of ${\varDelta } C(K\pi )$ in Set II.

In the following we will elaborate on the constraints posed by individual observables. For example, the general shape of the contour of branching fractions can easily be understood as follows: The leading contribution of the decay amplitude $\hat{\alpha }_4^c(K\pi ) = \alpha _4^c(K\pi ) + \beta _3^c(K\pi )$ is given as the sum of the QCD-penguin and the $\rho _A$-dependent WA amplitudes $\alpha _4^c$ and $\beta _3^c$, respectively. The experimental measurement of the branching fraction restricts $\hat{\alpha }_4^c$ to a circle in its imaginary plane

4.2 $$\begin{aligned} \sqrt{\mathcal{B}}&\simeq \lambda _c^{(s)} F_0^{B\rightarrow \pi } f_K \, \big |\hat{\alpha }^c_4\big | \, \big (1 + \mathcal{O}(r_i)\big ) \end{aligned}$$

where the $r_i = (r_\mathrm{T}^{},\, r_\mathrm{T}^\mathrm{C},\, r_\mathrm{EW}^{},\, r_\mathrm{EW}^\mathrm{C},\, r_\mathrm{EW}^\mathrm{A})$’s [15] are numerically small, mode-dependent corrections, normalized to $\hat{\alpha }_4^c$. Consequently, $\beta _3^c(\rho _A^2, \rho _A^{})$ can interfere constructively or destructively with $\alpha _4^c$ depending on the phase of $\rho _A^{K\pi }$. For $\phi _A^{K\pi } \sim 0,\, \pi$, the WA contribution is mainly real and contributes constructively to $\alpha _4^c$. However, the contributions to $\beta _3^c$ that are linear and quadratic in $\rho _A$ also interfere with each other either constructively ($\phi _A^{K\pi } \sim 0$) leading to small $|\rho _A^{K\pi }| \sim 2.0$ or destructively ($\phi _A^{K\pi } \sim \pi$), leading to larger $|\rho _A^{K\pi }| \sim 3.4$. On the other hand, large values $|\rho _A^{K\pi }| \sim 6.0$ are required for $\phi _A^{K\pi } \sim \pi /2,\, (3\pi /2)$ where $\beta _3^c$ becomes purely imaginary and interferes destructively with $\alpha _4^c$. In summary, the four branching-fraction measurements in Set I of the $B\rightarrow K \pi$ system can be described by a single universal $\rho _A$ and by themselves they do not exclude any value of the phase and allow up to $|\rho _A^{K\pi }|\lesssim 6$.

Whereas the branching fractions fix the modulus of

4.3 $$\begin{aligned} \hat{\alpha }_4^c&= |\hat{\alpha }_4^c|\,\exp (i\, \hat{\phi }_4^c), \end{aligned}$$

the ratios of branching fractions (see Eq. (2.15)) depend strongly on the real part of $\hat{\alpha }_4^c$, i.e., are sensitive to the phase $\hat{\phi }_4^c$

4.4 $$\begin{aligned} R_{c,n}^{B,K,\pi }&\simeq 1 + \cos \hat{\phi }_4^c \sum \limits _i c_i \text{ Re }(r_i) + \cdots \end{aligned}$$

Here the $$c_i$$ denote proportionality factors and terms proportional to $$\text{ Im }(r_i)$$ are denoted by dots. The latter become numerically important only in the vicinity of $$\hat{\phi }_4^c \sim \pi /2,$$$$ (3\pi /2)$$, and they are fully included in the fits. Hence these ratios are sensitive to flips of $$\hat{\phi }_4^c$$ by $$\pi $$. As can be seen in Fig. 1b, the data disfavors and excludes to a large extent the scenario of large WA when using observable Set II, i.e., purely imaginary $$\beta _3^c$$, which would interfere destructively with $$\alpha _4^c$$. There is no need for anomalously large WA contributions to describe $$B\rightarrow K\pi $$ data of branching fractions and their ratios in QCDF within the SM. Moreover, at 68 % probability the largest portion of allowed $$\rho _A^{K\pi }$$ parameter space is within $$\xi ^A_3(K\pi ) < 0.5$$.

We provide also separately the constraints from direct CP asymmetries. In QCDF the strong phase, necessary for CP violation, arises at $\mathcal{O}(\alpha _s)$, respectively $\mathcal{O}(1/m_b)$, and is thus included only to leading order in our numerical evaluations. Currently, CP asymmetries with neutral kaons in the final state are measured to be small with large errors, whereas the ones with charged kaons are observed to be large and with a relative opposite sign. For the latter decays, the leading terms to the CP asymmetries are from color-allowed, $r_\mathrm{T}$, and color-suppressed, $r_\mathrm{T}^\mathrm{C}$, penguin-to-tree ratios [15],

4.5 $$\begin{aligned} C(B^- \rightarrow K^- \pi ^0)\approx & {} 2\, \text{ Im } (r_\mathrm{T} + r_\mathrm{T}^\mathrm{C}) \sin \gamma \nonumber \\= & {} (-4.0 \pm 2.1) \% , \nonumber \\ C(\bar{B}^0 \rightarrow K^- \pi ^+)\approx & {} 2\, \text{ Im } (r_\mathrm{T}) \sin \gamma \nonumber \\= & {} (+8.2 \pm 0.6) \% , \end{aligned}$$

where the measured values are taken from [19], and $\gamma$ denotes the angle of the CKM unitarity triangle. Their difference is dominated by the color-suppressed tree amplitude

4.6 $$\begin{aligned} {\varDelta } C&\simeq 2\, \text{ Im } (r_\mathrm{T}^\mathrm{C}) \sin \gamma \simeq (-12.2 \pm 2.2) \% . \end{aligned}$$

In QCDF, one has

4.7 $$\begin{aligned} \text{ Im } (r_\mathrm{T})&\propto - \frac{\text{ Re }(\alpha _1)}{|\hat{\alpha }_4^c|} \sin \hat{\phi }_4^c + \frac{\text{ Im }(\alpha _1)}{|\hat{\alpha }_4^c|} \cos \hat{\phi }_4^c , \nonumber \\ \text{ Im } (r_\mathrm{T}^\mathrm{C})&\propto - \frac{\text{ Re }(\alpha _2)}{|\hat{\alpha }_4^c|} \sin \hat{\phi }_4^c + \frac{\text{ Im }(\alpha _2)}{|\hat{\alpha }_4^c|} \cos \hat{\phi }_4^c , \end{aligned}$$

where $\hat{\alpha }_4^c$ depends on $\rho _A$. The numerical values for

**Fig. 2:**
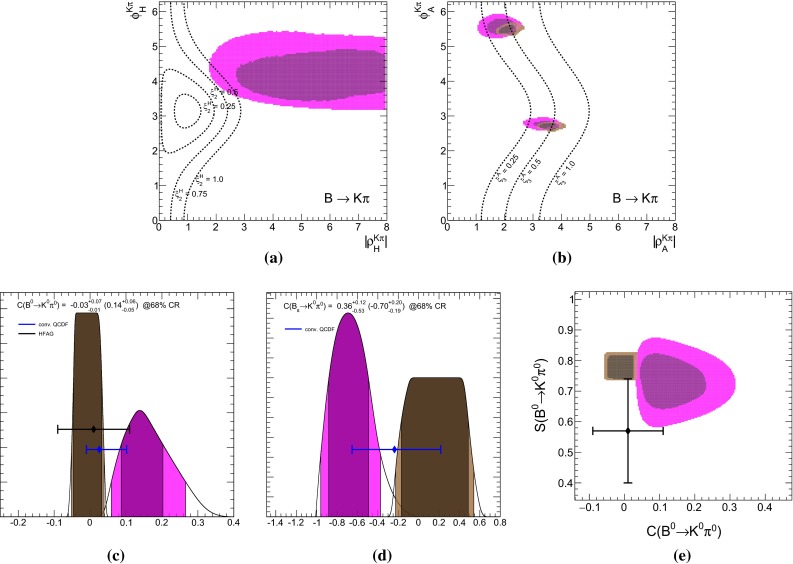
The 68 % (*dark*) and 95 % (*bright*) CRs of $\rho _H^{K\pi }$ (*upper left*) and $\rho _A^{K\pi }$ (*upper right*), obtained from a fit with partial (see text) observable Set II for $B\rightarrow K\pi$ decays, treating $\rho _H^{K\pi }$ either as a fit parameter (*purple*) or nuisance parameter as described in Appendix B.1 (*brown*). The *dashed lines* correspond to constant $\xi ^H_2(K\pi ) = (0.25, 0.5, 0.75, 1.0)$ and $\xi ^A_3(K\pi ) = (0.25, 0.5, 1.0)$, respectively. The *lower panels* show the predictions for $C(B_d\rightarrow K^0\pi ^0)$ (*left*) and $C(B_s \rightarrow K^0\pi ^0)$ (*middle*) and the correlation of $C(B_d\rightarrow K^0\pi ^0)$ with $S(B_d\rightarrow K^0\pi ^0)$ (*right*). The available experimental results are shown with $1\sigma$ errors and a prediction from QCDF with the conventional uncertainty estimate is labeled “conv. QCDF”. The 68 % credibility intervals for the predictions are given on the top of both panels for conventional $\rho _H$ (*brown*) and in brackets for fitted $\rho _H$ (*purple*)

4.8 $$\begin{aligned} 100 \cdot \frac{\alpha _1}{|\hat{\alpha }_4^c|}&\approx -17.4^{+1.0}_{-0.9} - i \, 0.4^{+0.6}_{-0.5},\nonumber \\ 100 \cdot \frac{\alpha _2}{|\hat{\alpha }_4^c|}&\approx -5.8^{+1.1}_{-3.4} + i \, 1.5^{+0.3}_{-0.5}\, - 2.1^{+0.3}_{-1.2}\, \rho _H^{K\pi }, \end{aligned}$$

hold for the central values as well as the variation of theory parameters and $\rho _A^{K\pi }$ at the best-fit point of Set II listed in Table 5. We kept explicitly the dependence of $r_\mathrm{T}^\mathrm{C}$ on the HS parameter $\rho _H^{K\pi }$, which is numerically irrelevant for $r_\mathrm{T}$. It can be seen that $r_\mathrm{T}^\mathrm{C}$ can be enhanced if $\text{ Re }(\rho _H^{K\pi }) > 0$ and $\text{ Im }(\rho _H^{K\pi }) < 0$—see later discussion concerning Fig. 2a.

The allowed regions obtained from a fit of only CP asymmetries in Fig. 1a, b (shown in green) are similar for Set I and Set II. The best-fit point is at $\rho _A^{K\pi } \approx 4.1 \exp (i\, 1.8)$, along the branch of $\phi _A^{K\pi } \sim \pi /2$ that gives rise to strong cancelations in destructive interference of $\alpha _4^c$ and $\beta _3^c$ and leads to large theoretical uncertainties, which in turn allows for good agreement with the data. Apart from the fact that branching fraction measurements would become incompatible at more than $30\sigma$, neglected higher order perturbative and power corrections would become important in these regions of parameter space putting into doubt the reliability of the prediction. However, there are substantial parts of the 68 % CRs with $\xi ^A(K\pi ) < 0.5$ and the size of WA contributions can be as low as 0.25, for which these comments do not apply.

The very same figures (Fig. 1a, b) show also that there is no or hardly any overlap at 95 % probability of the allowed regions from branching fractions (blue) and those from CP asymmetries (green). In our approach, the so-called “${\varDelta } {\mathcal{A}_\mathrm{CP}}$ puzzle” manifests itself only in the combined fit of branching fractions and CP asymmetries, where then large pull values arise for ${\varDelta } C$ (Set II), or equivalently also $C(B^- \rightarrow K^- \pi ^0)$ (Set I). These pull values are shown in Table 5 and caused by the higher statistical weight of the branching-fraction measurements. So one might wonder how previous QCDF analyses, for example [15, 57], arrived at a “${\varDelta } {\mathcal{A}_\mathrm{CP}}$ puzzle” based on the conventional approach, where $\rho _A$ is varied independently for each observable? The answer is rather simple: there the uncertainty of an observable is determined by the spread of values obtained in a scan of $\rho _A$ with $|\rho _A^{K\pi }| \approx 1$ and arbitrary phase $\phi _A^{K\pi }$. Moreover, the central value of the observable is usually assigned by definition to $\phi _A^{K\pi } = 0$. This corresponds in Fig. 1a, b to a line of constant $|\rho _A^{K\pi }| = 1$, which yields only consistent results for branching fractions, but never for the combination of CP asymmetries, i.e. those CP asymmetries which dominate statistically over the ones with large experimental errors. In the conventional approach there will be no “${\varDelta } {\mathcal{A}_\mathrm{CP}}$ puzzle” once larger values of $|\rho _A^{K\pi }| \lesssim 2$ and a fine stepsize for the variation of $\phi _A^{K\pi }$ are permitted in the scan, implying of course larger $\xi ^A(K\pi ) \lesssim 0.5$ and increased theoretical uncertainties. We note that $C(B^- \rightarrow K^0 \pi ^-)$ almost vanishes in QCDF and even in the presence of large power corrections it is difficult to increase the predictions beyond $1~\%$, such that the current pull of $1 \sigma$ (see Table 5) can hardly be reduced. We emphasize again the different assumptions underlying our approach, i.e., WA parameters are universal among decays related by $(u\leftrightarrow d)$ quark exchange, but need not be small and are determined from data, contrary to the conventional approach, i.e., WA parameters are scanned over a rather small range and the resulting errors correspond to non-universal parameters.

The results of the combined fit of branching fractions and CP asymmetries had been already discussed at the beginning of this section. Whereas CP asymmetries involved in the “${\varDelta } {\mathcal{A}_\mathrm{CP}}$ puzzle” exhibit larger pull values for both sets of observables Set I and Set II, predictions of branching fractions are in good agreement with the corresponding measurements, in part also due to large form factor uncertainties. The latter parametric dependence cancels to a large extent in the ratios of branching fractions and yields a large pull value of $-1.9\sigma$ for $R_n^B(K\pi )$ in Set II, which contributes also to the problematic *p* value of 0.04. In a fit of Set II without CP asymmetries we obtain pull values of $-1.2\sigma$ for $R_n^B(K\pi )$, $0.4\sigma$ for $R_c^K(K\pi )$ and $0.6\sigma$ for $R_c^\pi (K\pi )$, which can be compared to the pull in Table 5 when including CP asymmetries. This can also be seen in Fig. 1b, where the solution of the combined fit (red) at $\rho _A \sim 3.3 \exp (2.7\,i)$ does neither overlap with the 68 % CRs from $\mathcal{B}/R_{n,c}$ (blue) nor from $C/{\varDelta } C$ (green).

Finally, we explore in more detail the discrepancy in ${\varDelta } C$, departing from the conventional error estimate of power corrections of HS contributions that had been used until now; see Appendix A. As previously mentioned in Eq. (4.6), the color-suppressed tree amplitude $\alpha _2^u$ determines the magnitude of $r_\mathrm{T}^\mathrm{C} \propto |\lambda _u^{(s)}/\lambda _c^{(s)}| \, \alpha _2^u/\hat{\alpha }_4^c$. Possible large NNLO corrections might relax the tension in the case of destructive interference to the real and constructive interference to the imaginary part. For $\pi \pi$ final states, however, such NNLO vertex corrections are canceled by the NLO HS corrections [44–46, 50], which might not necessarily takes place to the same extent in $K\pi$ final states. Nevertheless, in the following we will assume no large perturbative higher order corrections and fit instead the phenomenological parameter $\rho _H^{K\pi }$ in addition to $\rho _A^{K\pi }$.

We point out that ${\varDelta } C$ depends on the sum $\alpha _{2,\mathrm {I}} + \alpha _{2,\mathrm {II}}^{\mathrm{tw}-2} + \alpha _{2,\mathrm {II}}^{\mathrm{tw}-3}(\rho _H) + \beta _2(\rho _A^i)$, where $\beta _2$ is dominated by building block $A_1^i$ and the corresponding WA parameter $\rho _A^i$—see Eq. (3.6). The contribution of $\beta _2$ is always much smaller than $\alpha _{2,\mathrm {II}}^{\mathrm{tw}-3}$ unless $\rho _H \ll \rho _A^i$. Hence we prefer to fit $\rho _H$ and assume $\rho _A^i = \rho _A^{}$, the common WA parameter of all building blocks $A_{1,2,3}^{i,f}$. Only for a rather large $\rho _A^i \gtrsim 4$ will the $\beta _2$ contribution be comparable to the theory uncertainties of the leading amplitudes in ${\varDelta } C$. Finally we note that as a consequence of our approximation, the effect of other neglected but potentially enhanced subleading corrections in the $1/m_b$ expansion will be contained in $\rho _H$ and $\rho _A$. For example large different subleading corrections from QCD penguins for the up and charm sectors, as found in [58], will be absorbed into $\beta _2(\rho _H)$ and $\beta _3(\rho _A)$ respectively. In view of the limited number of observables it is not possible to discern the various scenarios of enhanced subleading corrections with the $B\rightarrow K\pi$ system alone.

The results of a fit to the partial observable Set II for the parameters $\rho _H^{K\pi }$ and $\rho _A^{K\pi }$ is shown in purple in Fig. 2a, b, respectively. We have removed the CP asymmetry $C(B_d \rightarrow K^0\pi ^0)$ that is very sensitive to HS, but its current measurement does not provide any constraints, making it an ideal candidate for a prediction. In contrast, $R_n^B(K\pi )$ is not very sensitive to HS, but its large pull value would force $\rho _H^{K\pi }$ to large values, which might not be necessary to explain ${\varDelta } C$. For comparison we depict as brown contours also the ones from Fig. 1b and provide contour lines of constant $\xi _2^H(K\pi )$ and $\xi _3^A(K\pi )$.

We find that the prediction of ${\varDelta } C = -0.11^{+0.04}_{-0.02}$ at the best-fit point $\rho _H^{K\pi } = 3.3 \exp (3.7\, i)$ coincides, within experimental uncertainties, with the measurement Eq. (4.6). The preferred phase of $\phi _H^{K\pi } \sim (3\pi /2)$ implies that HS contributions to $\alpha _2^u(K\pi )$ are mainly imaginary and interfere constructively with the imaginary part of the vertex corrections. At the best-fit point, all observables have zero pull values except for a $0.8\sigma$ pull in $C(B^- \rightarrow \bar{K}^0 \pi ^-)$ and $R_n^B$, which had been discarded from the fit.

As can be seen in Fig. 2a, the contours of constant $\xi _2^H(K\pi )$ exhibit a different dependence on $\rho _H^{K\pi }$ as compared to $\xi _3^A$ for $\rho _A^{K\pi }$. Already in the conventional approach ($|\rho _H^{K\pi }| = 1.0$), $\xi _2^H(K\pi ) = 1$ is admitted in estimates of theoretical uncertainties, which is a remnant artifact of the parametrization $X_H \sim (1 + \rho _H^{K\pi })$. In the fit this contour line lies within the 95 % CR for $1.8 \lesssim |\rho _H^{K\pi }| \lesssim 2.8$ and for the smallest $|\rho _H^{K\pi }| = 1.8$ at $\phi _H^{K\pi } = 4.6$, the pull of ${\varDelta } C$ decreases, to $-1.0 \sigma$, compared to $2.8\sigma$ in the SM. Concerning the WA corrections shown in Fig. 2b, $|\rho _A^{K\pi }|$ is shifted toward lower values compared to Fig. 1b, which allows also for smaller $\xi _3^A(K\pi )$. The fit shows that these lower values of $|\rho _A^{K\pi }|$ are correlated with large values of $|\rho _H^{K\pi }|$.

Assuming that HS corrections are in fact responsible for the observed discrepancy in ${\varDelta } C$, similar effects should be observed for related decays, as for example in CP asymmetries $B_d \rightarrow K^0\pi ^0$ and analogously $B_s \rightarrow K^0\pi ^0$. In the latter decay, such effects should be enhanced due to a different hierarchy of CKM elements $|\lambda _u^{(s)} / \lambda _c^{(s)}| \ll |\lambda _u^{(d)} / \lambda _c^{(d)}|$. The predictions of both CP asymmetries are shown in Fig. 2c, d, respectively, with color coding as in Fig. 2a, b. Once measured, respectively measured with higher precision, both will allow one to test the assumption of large HS contributions to $B_{d,s}\rightarrow K\pi$ decays

4.9 $$\begin{aligned} C(B_d \rightarrow K^0\pi ^0)^{\mathrm{fit} \, \rho _H} &= + 0.14^{+0.06}_{-0.05} , \nonumber \\ C(B_d \rightarrow K^0\pi ^0)^{\mathrm{scan}\, \rho _H}&\in [-0.04,\, 0.04] , \end{aligned}$$

and

4.10 $$\begin{aligned} C(B_s \rightarrow K^0\pi ^0)^{\mathrm{fit}\, \rho _H} &= - 0.70^{+0.20}_{-0.19} , \nonumber \\ C(B_s \rightarrow K^0\pi ^0)^{\mathrm{scan}\, \rho _H}&\in [-0.17,\, 0.48] . \end{aligned}$$

The predictions labeled “fit $\rho _H$” and “scan $\rho _H$” are shown in purple and brown, respectively, whereas the QCDF prediction for the conventional approach (with scanned $\rho _A$) are labeled “QCDF” in Fig. 2c, d. At the current stage, the measurement of $C(B_d \rightarrow K^0\pi ^0)$ prefers smaller HS contributions although the uncertainty is still too large to draw a definite conclusion.

The correlation between $C(B_d\rightarrow K^0\pi ^0)$ and $S(B_d\rightarrow K^0\pi ^0)$ is shown in Fig. 2e. Whereas the conventional treatment of $\rho _H$ predicts rather small uncertainties for both observables, uncertainties are much larger when fitting $\rho _H$. Still, both predictions are clearly distinct since the large HS scenario yields large $C(B_d\rightarrow K^0\pi ^0)$ compared to the conventional treatment. These findings are in agreement with previous studies [58, 59].

A similar analysis of enhanced HS contributions [60] has found a best-fit point at $\rho _H^{K\pi } = 4.9 \exp (4.9\, i)$. Bearing in mind that different numerical input, e.g. $\lambda _B = 0.35$ GeV, has been used, their result lies in the ballpark of our 68 % CR. The very recent work [61] also deals with fits of WA and HS parameters $\rho _{A,H}$ in $B\rightarrow PP$ decays $(PP = \pi \pi ,\, K\pi ,\, KK)$ in the SM in the framework of QCDF. In our study one $\rho _A^{M_1 M_2}$ is considered for each of the three decay systems separately. Instead, in [61] one $\rho _A$ for building block $A_3^f$ (see Eq. (3.6)), $\rho _A^f$, and one for building blocks $A_1^i \approx A_2^i$, $\rho _A^i$, are used simultaneously, neglecting thus *SU*(3)-breaking corrections for all three $b\rightarrow d$ and $b\rightarrow s$ decay systems. Also in this case one finds that $\rho _A^f$ is rather strongly constrained with two solutions similar to the ones shown in Fig. 1. Concerning $\rho _H$, similar regions are found as in our Fig. 2a in scenario III of [61].

### Results for $B \rightarrow K \rho ,\, K^*\pi ,\, K^*\rho$

In this section we discuss decay systems obtained from the replacement of a pseudoscalar in $B\rightarrow K\pi$ by its vector meson equivalent $\pi \leftrightarrow \rho$ and $K\leftrightarrow K^*$. Indeed, QCDF implies some qualitative differences when changing the spin of the final-state particles, but since the parametrization of the decay amplitudes of all four decay systems is equal, one might expect the discussed features of the $B\rightarrow K\pi$ system to appear also in $B\rightarrow K^*\pi$ (*PV*), $B\rightarrow K \rho$ (*VP*)^3^ and $B\rightarrow K^*\rho$ (*VV*). Currently the experimental measurements are not as precise as for $B\rightarrow K\pi$, and no striking tensions are found as can be seen from the *p* values^4^ and pulls of observables in Table 5.

**Fig. 3:**
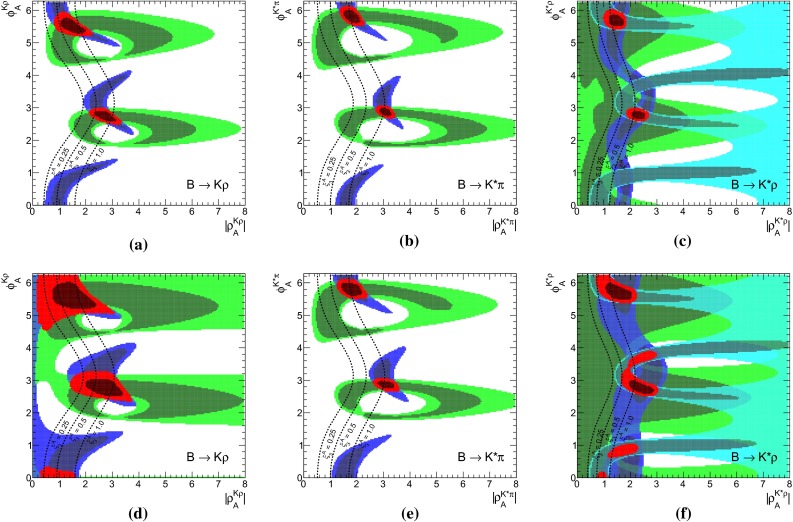
The 68 % (*dark*) and 95 % (*bright*) CRs of $\rho _A^{M_1 M_2}$ from a fit of observables in Set I (*upper*) and Set II (*lower*) of $B \rightarrow K \rho$ (*left*), $B \rightarrow K^* \pi$ (*middle*), and $B \rightarrow K^* \rho$ (*right*). Allowed regions are shown for $\mathcal{B}$ and $R_{n,c}$ (*blue*), *C* and ${\varDelta } C$ (*green*), $f_L$ (*cyan*) and their combination (*red*). The *dashed lines* correspond to constant $\xi ^A_3 = (0.25, 0.5,1.0)$ from *left* to *right*

The allowed regions of $\rho _A^{M_1 M_2}$ are shown in Fig. 3 for the observable Set I (upper panels) and Set II (lower panels). As before, the 68 and 95 % CRs allowed by fits from only $\mathcal{B}/R_{c,n}$ or only $C/{\varDelta } C$ and in addition for $M_1 M_2 = VV$ also only $f_L$ are color coded as blue, green and cyan, whereas the combined regions are depicted in red. As in the case of $B\rightarrow K\pi$, the combined constraints on $\rho _A^{M_1 M_2}$ from Set I and Set II observables are compatible with each other, but more stringent from Set I, especially for $B\rightarrow K\rho$ and $B\rightarrow K^* \rho$. Remarkably, the data of all four decay systems $M_1M_2 = K\pi ,\, K^*\pi ,\, K\rho ,\, K^*\rho$ prefers the same regions of $\phi _A^{M_1 M_2} \sim \pi ,\, 2\pi$, excluding large destructive interference of $\alpha _4^c(M_1 M_2)$ and $\beta _3^c(M_1 M_2)$. There is overlap at the 68 % probability level for all three systems for the solution $\phi _A \sim 2\pi$ and at 95 % probability for $\phi _A \sim \pi$. This is also supported by the data of $f_L$ in $B\rightarrow K^*\rho$, where the measurements of CP asymmetries are not very precise yet and otherwise no stringent constrains on $\rho _A^{K^*\rho }$ could have been obtained from branching-fraction measurements alone.

The relative amount of power corrections to the leading contribution for *PV*, *VP*, and *VV* final states is collected in Table 4 and indicated in Fig. 3 by contour lines of constant $\xi _3^A(M_1 M_2) = 0.25,\, 0.5,\, 1.0$. It is typically larger by a factor of $2-3$ compared to the *PP* final state in $B\rightarrow K\pi$, which is a qualitative feature of QCDF. The leading QCD-penguin flavor amplitude is a linear combination of the vector amplitude, $a_4$, and the chirally enhanced scalar QCD-penguin amplitude, $a_6$, [5]

4.11 $$\begin{aligned} \alpha _4 (M_1 M_2)&= a_4(M_1 M_2) \pm r_\chi ^{M_2} a_6(M_1 M_2) \end{aligned}$$

where the “$+$” sign applies to $M_1 M_2 = PP,\, PV$ and the “$-$” sign to $M_1 M_2 = VP,\, VV$ final states. The two contributions interfere destructively in the case $M_1 M_2 = VP$ leading to smaller QCD-penguin amplitudes than for $M_1M_2 = PP$. Further, the tree-level contribution to $a_6(M_1 M_2)$ vanishes for $M_2 = V$, again reducing $\alpha _4$ in $M_1M_2 = PV,\, VV$ compared to $M_1M_2 = PP$ giving implicitly rise to larger ratios $\xi _3^A(M_1 M_2)$. Values as low as $\xi _3^A(K\rho ) = 0.50$, $\xi _3^A(K^*\pi ) = 0.82$, and $\xi _3^A(K^*\rho ) = 0.93$ can be reached within the 68 % CRs of Set I observables, whereas even smaller values are allowed from Set II; see Table 4. Concerning decays with $K^*$ in the final state, the large values of $\xi _3^A$ are required mainly by measurements of branching fractions, whereas CP asymmetries and polarization fractions $f_L$ would allow for smaller values of $\xi _3^A$; see Fig. 3b, c.

The largest pull values arise for CP asymmetries $C(B^- \rightarrow \bar{K}^{*0} \pi ^-)$ with $+1.0\sigma$ and $C(B^- \rightarrow \bar{K}^0 \rho ^-)$ with $+0.7\sigma$. As in the case of $C(B^- \rightarrow \bar{K}^0 \pi ^-)$, these CP asymmetries almost vanish in our approximation and it is difficult to increase the predictions beyond $1~\%$, unless one considers additional large subleading corrections [58].

The advantage of observable Set II strongly depends on cancelation of theory uncertainties, as for example the form factors in the ratios of branching fractions. Especially in cases where WA contributions are large compared to the leading amplitude, i.e., large $\xi _3^A$, the reduction of uncertainties is less effective and there is no unambiguous preference for the use of either Set I nor Set II. Furthermore, the outcome of fits of Set I and Set II might differ depending strongly on the experimental measurements. Apart from that we are not aware of a specific reason for the qualitative differences between fits of Set I and Set II for the $B \rightarrow K\pi ,\, K^* \pi$ systems compared to $B \rightarrow K\rho ,\, K^*\rho$ systems. As can be seen from Table 5, pull values from Set II are in general slightly larger than from Set I.

### Other decays and comments on $B\rightarrow K\omega ,\, K^*\phi$

We tested our assumption of universal WA also with data listed in Tables 1, 2, and 3 for other QCD-penguin-dominated decay modes mediated by $b\rightarrow (d, s)$ transitions. For these decays, the analysis is restricted to observable Set I, where in most cases the experimental accuracy is poorer than for previously studied $B\rightarrow K\pi ,\, K\rho ,\, K^*\pi ,\, K^*\rho$ systems. The ranges for the ratios $\xi _3^A(M_1 M_2)$ that are required by the data are listed in Table 4, which have been commented previously. For all systems, again preferred regions appear for $\phi _A^{M_a M_b} \sim \pi ,\, 2\pi$, and in some cases also $\phi _A^{M_a M_b} \sim \pi /2,\, (3\pi /2)$ is still allowed.

**Fig. 4:**
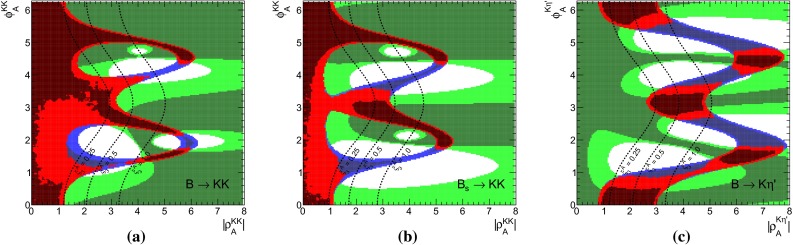
The 68 % (*dark*) and 95 % (*bright*) CRs of $\rho _A^{M_1 M_2}$ from a fit of observables in $B \rightarrow PP$: **a** $B\rightarrow KK$ (penguin dominated), **b** $B_s \rightarrow KK$ and **c** $B\rightarrow K \eta '$. Allowed regions are shown for $\mathcal{B}$ (*blue*), *C* (*green*), and their combination (*red*). The *dashed lines* correspond to constant $\xi ^A_3 = (0.25, 0.5, 1.0)$

The allowed regions for $B\rightarrow PP$ systems $B\rightarrow KK,\, K\eta '$ and $B_s \rightarrow KK$ are shown in Fig. 4 and for $B_s \rightarrow K^-\pi ^+$ in Fig. 1c. We do not show $B\rightarrow K \eta$ for which the data is even less constraining. Except for $B_s \rightarrow K^-\pi ^+$, the measurements of CP asymmetries are very poor and provide only little additional constraints to the ones of branching fractions. The preferred regions of WA contributions for $B_{d,s} \rightarrow KK$ look very alike supporting the assumption of universal WA for $B_d$ and $B_s$ decays into same final states, entertained in Sect. 4.5. In comparison, for $B_{d,s} \rightarrow K\pi$ (Fig. 1a, c) this might not seem the case, however, here one should compare the result of the fit to $B_d \rightarrow K^-\pi ^+$ only rather than the combination of all $B\rightarrow K\pi$ decays shown in Fig. 1a, b.

**Fig. 5:**
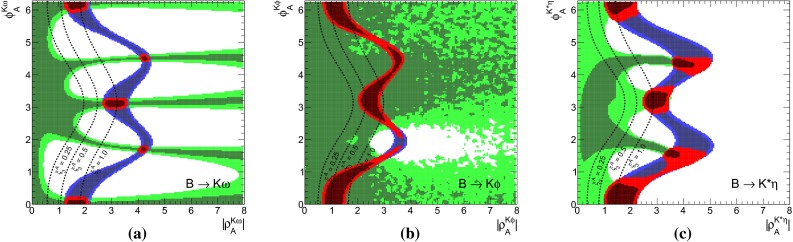
The 68 % (*dark*) and 95 % (*bright*) CRs of $\rho _A^{M_1 M_2}$ from a fit of observables in $B \rightarrow PV$: **a** $B\rightarrow K\omega$, **b** $B \rightarrow K\phi$ and **c** $B\rightarrow K^* \eta$. Allowed regions are shown for $\mathcal{B}$ (*blue*), *C* (*green*), and their combination (*red*). The dashed lines correspond to constant $\xi ^A_3 = (0.25, 0.5, 1.0)$

In Fig. 5 the allowed regions for $B\rightarrow VP$ systems $B\rightarrow K\omega ,\, K\phi ,\, K^*\eta$ are shown, whereas $B\rightarrow K^*\eta '$ has been omitted due to the poor constraints from the respective data. The measurements of branching fractions provide in all three cases already appreciable constraints. Concerning $B\rightarrow K\omega$, no tensions are observed. In case of $C(\bar{B}^0\rightarrow \bar{K}^0 \omega ^0)$, we included the HFAG average of the two incompatible measurements of Belle: $C = 0.36\pm 0.19 \pm 0.05$ [29] and BaBar: $C = -0.52^{+0.22}_{-0.20} \pm 0.03$ [63], which differ by $2.9\sigma$. The HFAG value $C = -0.04 \pm 0.14$ [19] indeed coincides with the theory prediction at the best-fit point ($C = -0.02 \pm 0.08$). One might hope that improved measurements at Belle II will settle this problem. As ${\varDelta } C(K\pi )$, this CP asymmetry is sensitive to the analogous color-suppressed tree amplitude $\alpha _2^u(K\omega )$ and might provide further tests of large HS contributions, which would be clearly visible. As in fact the largest uncertainty in the theory prediction is due to $\rho _H^{K\omega }$. It must be noted that although the best-fit point at $\rho _A^{K\omega } = 4.2 \exp (i\, 1.7)$ corresponds to a large $\xi _3^A(K\omega ) = 2.7$, other solutions at 68 % probability with $\xi _3^A(K\omega ) \lesssim 1$ provide equally vanishing pulls of observables.

**Fig. 6:**
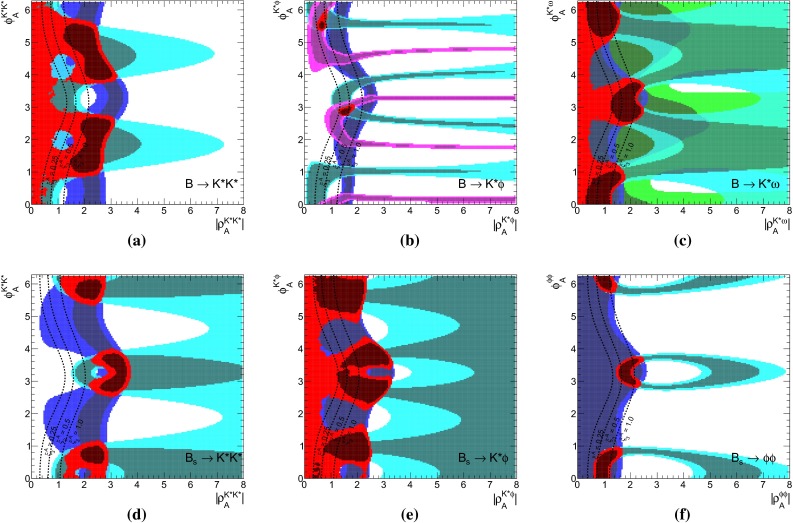
The 68 % (*dark*) and 95 % (*bright*) CRs of $\rho _A^{M_1 M_2}$ from a fit of observables in $B \rightarrow VV$: **a** $B\rightarrow K^*K^*$, **b** $B \rightarrow K^*\phi$, **c** $B\rightarrow K^* \omega$, and **d** $B_s\rightarrow K^*K^*$, **e** $B_s \rightarrow K^*\phi$, **f** $B_s\rightarrow \phi \phi$. Allowed regions are shown for $\mathcal{B}$ (*blue*), $C_h$ (*green*), $f_h$ (*cyan*), $\phi _h$ (*purple*) and their combination (*red*). The *dashed lines* correspond to constant $\xi ^A_3 = (0.25, 0.5, 1.0)$ from *left* to *right*

Finally, the allowed regions for the $B\rightarrow VV$ systems $B\rightarrow K^*K^*,\, K^*\phi ,\, K^*\omega$ and $B_s \rightarrow K^*K^*,\, K^*\phi ,\, \phi \phi$ are shown in Fig. 6. For $MM = K^*K^*$ final states the measurements of branching fractions require rather large WA contributions, contrary to the other considered *VV* final states. In all cases, the polarization fractions provide orthogonal constraints, which prefer $\phi _A^{M_a M_b} \sim \pi ,\, 2\pi$, except for $B \rightarrow K^*K^*$. For the moment measurements of CP asymmetries are only available for $B\rightarrow K^*\omega$ and the very recent LHCb measurements for $B\rightarrow K^*\phi$ [38]. They are compatible with zero and do not provide constraints yet since the theory predicts also rather small values.

Concerning $B\rightarrow K^*\phi$, we include in addition also available measurements of relative amplitude phases $\phi _{\perp ,\parallel }$ (purple). The combined allowed region from all observables does not overlap with regions from only branching fractions nor only amplitude phases at 68 % probability, giving rise to large pull values of the branching fraction $\mathcal{B}(B^0 \rightarrow K^{*0} \phi )$: $1.7\sigma$ from BaBar [36] and $2.6\sigma$ from Belle [37]; for $C_L(B^-\rightarrow K^{*-}\phi )$ of $-1.5\sigma$ from HFAG [19]; for $C_\perp (\bar{B}^0\rightarrow \bar{K}^{*0}\phi )$ of $1.2\sigma$ from Belle [37], but not for BaBar ($0.2\sigma$) and LHCb ($-0.6\sigma$); and for $\phi _\perp (\bar{B}^0\rightarrow \bar{K}^{*0}\phi )$ of $1.1\sigma$ from LHCb [38], but not for BaBar and Belle (both $0.0\sigma$). However, the *p* value of 0.95 of the fit is very high as we include many other measurements that are described consistently in the fit.

Due to a hierarchy of the helicity amplitudes in QCDF $A_L : A^- : A^+ = 1 : 1/m_b : 1/m_b^2$ [51] for the SM operator basis Eq. (3.2) the following relation should hold:

4.12 $$\begin{aligned} \phi _\perp= & {} \phi _\parallel . \end{aligned}$$

The experimental situation supports this within current errors. Since the hierarchy of helicity amplitudes does not hold in the presence of chirality-flipped operators beyond the SM, the measurement provides strong constraints on such scenarios. Further, QCDF predicts only small differences for neutral and charged decay modes such that one expects similar predictions for observables in both modes, even in the presence of NP contributions.

### WA-dominated $B \rightarrow K^+ K^-$ and $B_s \rightarrow \pi ^+ \pi ^-$

So far we discussed decays that are dominated by QCD-penguin topologies. They share the feature that leading WA contributions $\beta _3^c(M_1 M_2)$ are dominated by the building block $A_3^f$ (see Eq. (3.6)), which originates from gluon emission off the quark current in the final state. Furthermore, we grouped the decays that are related by $(u\leftrightarrow d)$-quark exchange, and assumed for each group one universal WA parameter $\rho _A$.

Now we are interested in decay modes that are governed solely by WA topologies. The only measured systems are so far $B\rightarrow K^+ K^-$ and $B_s\rightarrow \pi ^+ \pi ^-$. Their amplitudes are given by

4.13 $$\begin{aligned} \begin{aligned} \mathcal{A}(B \rightarrow K^+ K^-)&\simeq f_{B_d} f_K^2 \sum _p \lambda _p^{(d)} B^p_{K^+ K^-} ,\\ \mathcal{A}(B_s \rightarrow \pi ^+ \pi ^-)&\simeq f_{B_s} \, f_\pi ^2 \, \sum _p \lambda _p^{(s)} B^p_{\pi ^+\pi ^-} , \end{aligned} \end{aligned}$$

with

4.14 $$\begin{aligned} B^p_{M_1 M_2}&= \left( \delta _{pu} b_1 + 2 b_4^p + \frac{1}{2} b_{4, \mathrm EW}^p \right) . \end{aligned}$$

Since they are independent of quantities like form factors and the inverse moment of the *B*-meson DA, which cause usually large uncertainties, the precision of the determination of $\rho _A^{M_1 M_2}$ from the fit is mainly dictated by the experimental precision. The involved coefficients $b_i(M_1 M_2)$ depend exclusively on the building blocks $A_{1,2}^i(M_1 M_2)$ (see Eq. (3.6)) where the gluon is emitted off the quark current of the initial state, and they are thus in principle different from $A_3^f$, which dominates the penguin-dominated decays. Moreover, $A_1^i \approx A_2^i$ for $MM = PP$ final states when restricting to the asymptotic forms of the light-meson DAs [5].

**Fig. 7:**
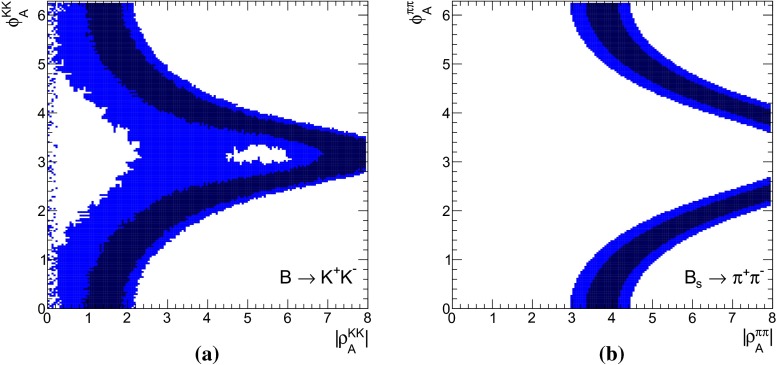
The 68 % (*dark*) and 95 % (*bright*) CRs for $\rho _A^{M_1 M_2}$ obtained from the branching fraction of WA dominated decays **a** $B \rightarrow K^+K^-$ and **b** $B_s \rightarrow \pi ^+\pi ^-$

The contours of $\rho _A^{K^+K^-}$ and $\rho _A^{\pi ^+\pi ^-}$ from the branching-fraction measurement are shown in Fig. 7a, b, respectively. Contrary to the penguin-dominated decays, the shape of the contour from the branching fractions is different, reaching large values $|\rho _A^{M_1 M_2}|$ for phases $\phi _A^{M_1 M_2} \sim \pi$, whereas for $\phi _A^{M_1 M_2} \sim 0$ the absolute value can be restricted: $|\rho _A^{K^+K^-}| \in [0.9, 1.9]$ and $|\rho _A^{\pi ^+\pi ^-}|\in [3.4, 4.1]$ at 68 % probability. While it is possible to have $|\rho _A^{K^+K^-}| \lesssim 2$ for small phases, as is the case for the previously considered penguin-dominated decays, the data requires $|\rho _A^{\pi ^+\pi ^-}| \gtrsim 3$ for any value of $\phi _A^{\pi ^+ \pi ^-}$, and, indeed, the contours of $\rho _A^{K^+K^-}$ and $\rho _A^{\pi ^+\pi ^-}$ do not overlap within the 95 % CR. Our results are in agreement with similar fits [64].

Apart from the mismatch of WA contributions for different initial and final states, there might be another interesting aspect, which can be studied in these decays. Namely, the amplitudes Eq. (4.13) are proportional to one overall CP-conserving strong phase due to the fact that the single amplitudes $b_i^p(M_1 M_2)$ (see Eq. (3.6)) depend on the same CP-conserving strong phase of $A_1^i$ due to the aforementioned relation $A_1^i \approx A_2^i$. Hence Eq. (4.14) becomes

4.15 $$\begin{aligned} B^p_{PP}&\approx \frac{C_F}{N_c^2} A_1^i \left( \delta _{pu} C_1 + 2 (C_4 + C_6) + \frac{C_{10} + C_8}{2} \right) . \end{aligned}$$

This is contrary to the requirement of at least one relative strong phase between the CP-conserving and CP-violating part of the amplitude in order to have a nonvanishing CP asymmetry. In consequence CP asymmetries vanish, up to neglected subleading corrections and no complementary information can be gained on the phases $\phi _A$ apart from the one of the branching fractions. An observation of direct CP violation would therefore point toward additional enhanced subleading corrections, as for example reported in [58] and allow one to discern them from the once due to the WA contribution considered here.

The measurement of other WA-dominated decay modes with *PV* and *VV* final states can help to further scrutinize WA contributions. For example for $M_1 M_2 = PV$, one has $A_1^i \approx - A_2^i$ [5] yielding

4.16 $$\begin{aligned} B^p_{PV}&\approx \frac{C_F}{N_c^2} A_1^i \left( \delta _{pu} C_1 + 2 (C_4 - C_6) + \frac{C_{10} - C_8}{2} \right) , \end{aligned}$$

whereas for $MM = VV$ final states $A_1^{i, h} \approx A_2^{i, h}$ ($h = L, +, -$) [23]. In the SM, the Wilson coefficients interfere destructively in $MM = PV$ and constructively in $MM = (PP,\, VV)$ decay modes. These decays are in principle sensitive to physics beyond the SM in $O_1$ and the color-octet operators $O_{4,6,8,10}$.

### Universal WA for $B_d$ and $B_s$ decays to same final states

So far, we have assumed one universal parameter for WA contributions of QCD-penguin-dominated decays that are related by $(u \leftrightarrow d$)-quark exchange, i.e., those groups of decays gathered in Tables 1, 2 and 3. For the purpose of this section, we will study effects which arise from the additional assumption of a universal WA parameter $\rho _A$ for decays into same final states mediated by the same quark currents at the weak interaction vertex. This implies in general relations between $|{\varDelta } S| = 1$ and $|{\varDelta } D| = 1$ decays.

In QCDF this assumption might be justified bearing in mind that WA contributions in QCD-penguin-dominated decay amplitudes are numerically dominated by topologies in which the gluon is emitted from the quark current that hadronizes into the final states, namely $A_3^f$ in Eq. (3.6). In this case the momentum transfer from the initial *B* meson is solely present at the weak interaction vertex, rendering the final-state hadronization independent of the flavor of the initial-state spectator quark. Therefore, one can expect that the difference between WA amplitudes in $B_d \rightarrow M_a M_b$ and $B_s \rightarrow M_a M_b$ decays might be of the order $\sim (m_{B_s} - m_{B_d})/m_{B_s} \approx m_s/m_b$. Similar arguments had been presented for the decays $B_d \rightarrow K^+ \pi ^-$ and $B_s \rightarrow K^+ \pi ^-$ in [65].

Currently experimental information is limited for $$B_s$$ decays to final states $$M_a M_b = K\pi ,\, KK,\, K^*\phi ,$$$$K^*K^*$$, whereas for $$M_a M_b = \phi \phi $$ the corresponding measurements for the $$B_d$$ is lacking. We do not consider $$B_s \rightarrow \pi ^+ \pi ^-$$, which is WA dominated and was discussed in Sect. 4.4, and further the corresponding $$B_d \rightarrow \pi ^+ \pi ^-$$ decay is tree-dominated. For $$B_{d,s} \rightarrow KK$$, the 68 % CRs overlap nicely as can be seen from the comparison of Fig. 4a, b. In the case of $$B_{d,s} \rightarrow K^* K^*$$, branching-fraction measurements are compatible, but regions from polarization-fraction measurements that are favored for $$B_d$$ decays are excluded for $$B_s$$ decays as shown in Fig. 6a, d. In consequence, 68 % CRs in $$B_{d,s} \rightarrow K^* K^*$$ overlap only marginally.

This leaves us mainly with the final-state system $K\pi$ to explore in more detail the consequences of the assumption of universal WA in decays with same final states, since for $K^*\phi$ the experimental information for the $B_s$ decay is not yet accurate enough to derive conclusive insights on this assumption. Especially we would like to test whether the CP asymmetry $C(B_s \rightarrow K^+\pi ^-)$, which had been measured recently by CDF [66] and LHCb [67], can be predicted correctly from WA contributions determined in $B \rightarrow K\pi$ decays.

**Fig. 8:**
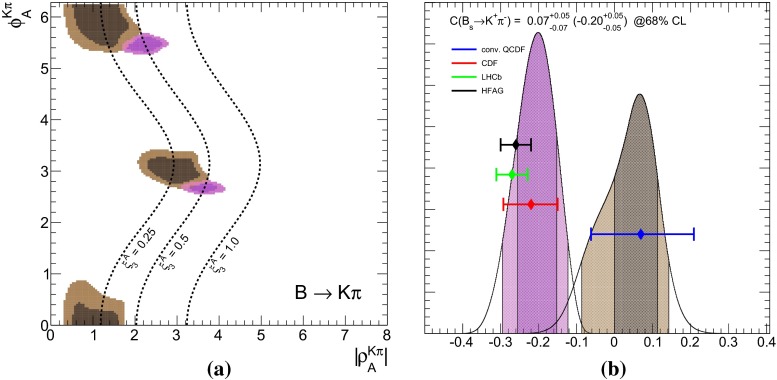
The 68 % (*dark*) and 95 % (*bright*) CRs for $\rho _A^{K\pi }$ (*left*), obtained from a fit with the reduced observable set for $B^- \rightarrow K^-\pi ^0$ (*brown*) and $B_d \rightarrow K^+\pi ^-$ (*purple*) (see text) assuming the SM. The dashed lines correspond to constant $\xi _3^A(K\pi ) = (0.25,\, 0.5,\, 1.0)$. The *right panel* shows the predictions for the direct CP asymmetry $C(B_s \rightarrow K^+ \pi ^-)$ for the two fit regions of $\rho _A^{K\pi }$ in the *left panel* using the same color coding. Experimental results are shown with $1\sigma$ errors and the prediction from QCDF with conventional uncertainty estimates is labeled “QCDF”

As discussed before in Sect. 4.1, the fit for $B\rightarrow K\pi$ does not allow for a simultaneous explanation of the two CP asymmetries $C(B^- \rightarrow K^-\pi ^0)$ and $C(\bar{B}^0 \rightarrow K^-\pi ^+)$. For this purpose we determine $\rho _A^{K\pi }$ separately from the combination of the branching fraction and CP asymmetry for each of the two contradicting decays. In addition we used $R_c^K(K\pi )$ to suppress solutions from the large WA scenario. The best-fit regions of $\rho _A^{K\pi }$ are shown in Fig. 8a where the contour from $\bar{B}^0 \rightarrow K^-\pi ^+$ coincides nicely with the one in Fig. 1b, where all constraints had been combined, due to the higher statistical weight of $C(\bar{B}^0 \rightarrow K^-\pi ^+)$. We note that $|\rho _A^{K\pi }| > 1$ does originate from the precise measurement of $C(\bar{B}^0 \rightarrow K^-\pi ^+)$, contrary to $C(B^- \rightarrow K^-\pi ^0)$, which allows also smaller values of $|\rho _A^{K\pi }|$ as can be seen in Fig. 8a.

Based on our assumption, we predict from both fits the CP asymmetry $C(B_s \rightarrow K^+\pi ^-)$; see Appendix B.4 for details. As shown in Fig. 8b, the measurements agree with the prediction from the $(K^-\pi ^+)$-fit whereas it fails at more than $4\sigma$ for the $(K^-\pi ^0)$-fit. In this case data supports the assumption that WA might be universal for decays with the same final states. It will be interesting to test these assumption further against improved measurements in the future. On the other hand this result shows that giving up the universality of the WA parameter for final states related by $(u\leftrightarrow d)$ exchange, but still insisting on a universal parameter for same final states would also resolve the “${\varDelta } {\mathcal{A}_\mathrm{CP}}$ puzzle”.

## New physics scenarios

In the framework of the SM, our analysis in the previous Sect. 4 has shown that the data of all investigated systems can be described with one universal WA parameter per system of decays that are related by ($u\leftrightarrow d$) quark exchange, apart from stronger tensions in $B\rightarrow K\pi$ and in $B\rightarrow K^*\phi$. This section is devoted to the attempt to constrain new-physics parameters in fits of the data simultaneously with the determination of one universal WA parameter per system using data from $B\rightarrow K\pi ,\, K\rho ,\, K^*\pi ,\, K^*\rho$, and $K^*\phi$, i.e., in total five WA parameters $\rho _A^{M_a M_b}$. In the presence of additional degrees of freedom of the NP parameters, one can expect that tensions present in the SM fit will be relaxed and the size of power corrections ($\xi _3^A$) can be decreased further.

We choose a model-independent approach, assuming NP contributions to Wilson coefficients of operators present in the SM operator basis Eq. (3.1) and their chirality-flipped counterparts obtained by $(1-\gamma _5)\leftrightarrow (1 + \gamma _5)$ interchange. The $B\rightarrow M_1 M_2$ matrix elements of the chirality-flipped operators can be obtained from the non-flipped ones via parity transformations [68]

5.1 $$\begin{aligned} \langle M_1 M_2 | O_i'| B \rangle&= - \eta _{M_1 M_2} \langle M_1 M_2 | O_i'| B \rangle \end{aligned}$$

with $\eta _{M_1 M_2} = + 1$ for $M_1 M_2 = PP,\, VV$ final states and $\eta _{M_1 M_2} = -1$ for $M_1 M_2 = PV,\, VP$ final states. In this case $b_i'(M_1 M_2) = b_i(M_1 M_2)[C_i \rightarrow C_i']$; see Eq. (3.6), and analogous relations hold for $a_i'(M_1 M_2)$. In the case of positive/negative polarized final states, form factors and decay amplitudes have to be replaced by their helicity-flipped counterpart e.g., $F_\pm \leftrightarrow F_\mp$ and $A_\pm (M_1 M_2) \leftrightarrow A_\mp (M_1 M_2)$.

In Sect. 5.1 we explore new-physics contributions to the Wilson coefficients of color-singlet QED-penguin Wilson coefficients $C_{7,9}$ and their chirality-flipped counterparts $C_{7,9}'$. They are well-known solutions of the “${\varDelta } {\mathcal{A}_\mathrm{CP}}$ puzzle” in $B\rightarrow K\pi$ [69, 70] and here we further investigate the compatibility of such NP contributions with data of the four other aforementioned decay systems. As a second model-independent scenario we consider NP contributions in the Wilson coefficients of the tree-level $b\rightarrow s\,\bar{u}u$ operators in Sect. 5.2. In the SM, they are doubly Cabibbo-suppressed $\sim \lambda _u^{(s)}/\lambda _c^{(s)}$ in all CP-averaged observables in $b\rightarrow s$ transitions, but give leading contributions to CP asymmetries. The investigation of further scenarios that involve also complementary constraints from exclusive $b\rightarrow s\,(\gamma ,\, \bar{\ell }\ell )$ decays are given in [71].

### NP in QED penguins

The QED-penguin operators $O_{7,\ldots , 10}$, see Eq. (3.1), and their chirality-flipped counterparts $O'_{7,\ldots , 10}$ are isospin-violating. Compared to the SM, NP contributions can relax the encountered tensions in ${\varDelta } C(K\pi )$ and $R_n^B(K\pi )$ and here we combine $B\rightarrow K\pi$ data with additional measurements from the aforementioned decay systems. We will focus on the color-singlet operators $i = 7,7',9,9'$ since the matching contributions to Wilson coefficients of the color-octet operators $i = 8,8',10,10'$ are suppressed by the strong coupling $\alpha _s$. Moreover, in the SM the chirality structure yields very small $C_7$ and large $C_9$, which must not be the case for NP scenarios. Depending on the final state, the two linear combinations $\overline{C}_i \equiv (C_i + C'_{i})$ and ${\varDelta } C_i \equiv (C_i- C'_{i})$ can be tested in $MM = PV$ and $MM = PP,\, V_L V_L$, respectively.

We introduce NP contributions to the Wilson coefficients at the matching scale $\mu _0 = M_W$ that we set to the mass of the *W*-boson and for practical purposes we rescale them with the SM value $C_9^\mathrm{SM}(\mu _0) = -1.01 \alpha _e$

5.2 $$\begin{aligned} C_i(\mu _0)&= C_i^\mathrm{SM}(\mu _0) + |C_9^\mathrm{SM}(\mu _0)|\, \mathcal{C}_i \end{aligned}$$

for $i = 7,7',9,9'$. We consider several sub-scenarios

- Single operator dominance Sc-*i*: $\mathcal{C}_i \ne 0$ and $\mathcal{C}_{j \ne i} = 0$ for $i = 7,7',9,9'$
- Parity (anti-)symmetric scenario Sc-$77'$: $\mathcal{C}_{7,7'} \ne 0$ and $\mathcal{C}_{9,9'} = 0$ Sc-$99'$: $\mathcal{C}_{9,9'} \ne 0$ and $\mathcal{C}_{7,7'} = 0$
- (Axial-)vector coupling scenario Sc-79: $\mathcal{C}_{7,9}\,\,\, \ne 0$ and $\mathcal{C}_{7',9'} = 0$ Sc-$7'9'$: $\mathcal{C}_{7',9'} \ne 0$ and $\mathcal{C}_{7,9}\,\,\, = 0$
- Generic scenario Sc-$77'99'$: $\mathcal{C}_i \ne 0$

with complex-valued $\mathcal{C}_i$. Although we introduce a NP parameterization at the matching scale, RG evolution will not lead to mixing of QED penguin operators into QCD and tree-level operators $i = 1,\ldots ,6$ at the order considered here. Thus NP contributions will not modify the leading amplitude $\hat{\alpha }_4^c$, but only $\alpha _{3(4),\mathrm{EW}}^p$ and the WA amplitudes $\beta _{3(4),\mathrm{EW}}^p$. Consequently, branching fractions will become modified only slightly, whereas CP asymmetries can deviate substantially from their SM predictions for nonzero CP-violating phases.

As long as NP contributions do not become very large compared to $\hat{\alpha }_4^c$ one might still employ the expansion in small mode-dependent ratios

5.3 $$\begin{aligned} r_i&= r_{i,\mathrm{SM}} + \sum _j r_{i,j}\,\overline{\mathcal{C}}_j, \end{aligned}$$

see Eq. (4.2), in which the NP contributions $r_i$ depend linearly on the complex NP parameters $\mathcal{C}_j \equiv |\mathcal{C}_j|e^{i \delta _j}$. In particular [15]

5.4 $$\begin{aligned} C(B^- \rightarrow \bar{K}^0 \pi ^-)&\simeq \sum _{j = 7,9}\! \text{ Im } \left( \frac{2}{3} r_{\mathrm{EW},j}^\mathrm{C} - \frac{4}{3} r_{\mathrm{EW},j}^\mathrm{A} \right) \text{ Im }\, \overline{\mathcal{C}}_j\, , \nonumber \\ {\varDelta } C - {\varDelta } C^\mathrm{SM}&\simeq \sum _{j = 7,9}\! \text{ Im } \left( -2r_{\mathrm{EW},j}^{} -2r_{\mathrm{EW},j}^\mathrm{A} \right) \text{ Im }\, \overline{\mathcal{C}}_j . \end{aligned}$$

Numerically one has approximately

5.5 $$\begin{aligned} \begin{array}{l|cc} \text{ Im } (r_{i,j}) \times 10^2 &{} j = 7 &{} j = 9 \\ \hline i = \text{ EW } &{} +2.0^{+0.7}_{-0.8} &{} -2.0^{+0.8}_{-0.7} \\ i = \text{ EW,C } &{} -1.7^{+0.6}_{-0.5} &{} +0.3^{+1.2}_{-1.2} \\ i = \text{ EW,A }\; (\rho _A^{\text{ fit }}) &{} +6.5^{+0.5}_{-0.5} &{} -0.06 \pm 0.04 \\ i = \text{ EW,A }\; (\rho _A^{\text{ scan }}) &{} +0.8^{+5.4}_{-7.4} &{} +0.1^{+0.9}_{-0.9} \end{array} \end{aligned}$$

in which we used the best-fit point of $\rho _A^{K\pi }$ that was obtained from the SM fit with Set II in the case of $i \in (\text{ EW };\, \text{ EW,C })$ and no variation of $\phi _A^{K\pi }$ is included in the determination of theory uncertainties. The case of $r_{\mathrm {EW}, j}^\mathrm{A}$ is more involved due to the explicit dependence on the WA parameter and we provide two points: (1) for $\rho _A^{K\pi }$ obtained from the SM fit as above, denoted as $\rho _A^{\text{ fit }}$ in Eq. (5.5) and (2) for $\phi _A^{K\pi } = 0$, denoted as $\rho _A^{\text{ scan }}$ in Eq. (5.5), as usually chosen in conventional QCDF as central value including the variation of $\phi _A^{K\pi }$ into the error estimation. Several observations can be made:

1. Given that $\text{ Im }\, \overline{\mathcal{C}}_{7,9} \sim \mathcal{O}(1)$, the numerical coefficients imply that the total amount of CP violation from $r_{i,j}$ of $i \in (\text{ EW };\, \text{ EW,C })$ does not exceed $3.5~\%$, whereas $r_\mathrm{{EW},9}^\mathrm{C}$ is numerically negligible.
2. An accidental cancelation can be observed in $(r_\mathrm{{EW}, 7}^{} + r_\mathrm{{EW},7}^\mathrm{C})$ as well as in $(r_\mathrm{{EW}, 7} + r_\mathrm{{EW}, 9})$ if $\text{ Im }\, \overline{\mathcal{C}}_{7} \approx \text{ Im }\, \overline{\mathcal{C}}_{9}$.
3. The amount of CP violation from $\text{ Im }\, \overline{\mathcal{C}}_9$ to $r_\mathrm{EW}^\mathrm{A}$ can be neglected in both cases (1) and (2), whereas the contribution of $\text{ Im }\, \overline{\mathcal{C}}_7$ can indeed become large.
4. Since the measurement of $C(B^- \rightarrow \bar{K}^0 \pi ^-) = (1.5 \pm 1.9)~\%$ is rather accurate, it forbids too large CP-violating contributions from $\text{ Im }\, \overline{\mathcal{C}}_7$ if $\rho _A$ is fitted.

**Fig. 9:**
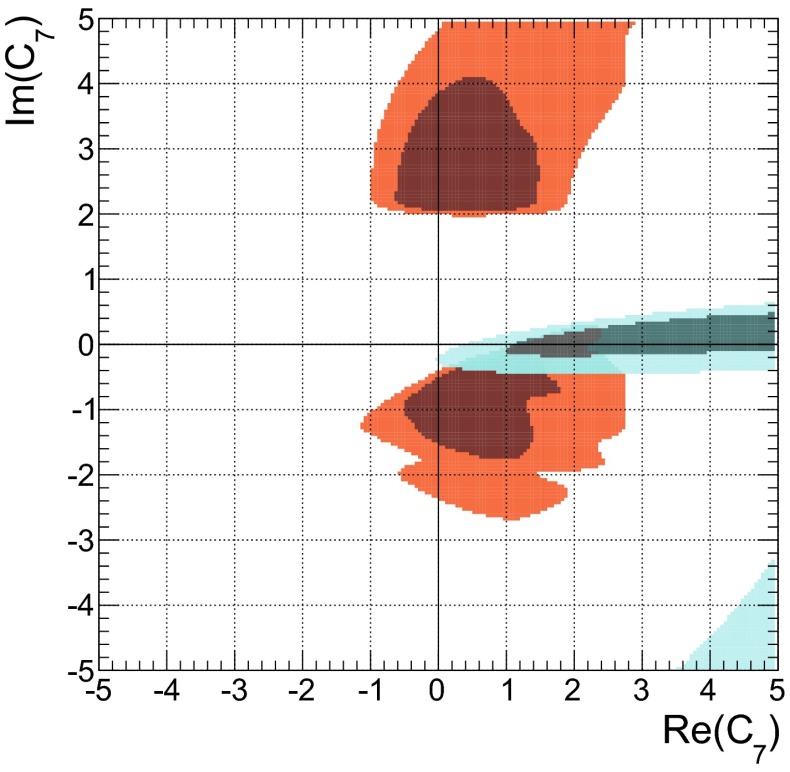
The 68 % (*dark*) and 95 % (*bright*) CRs for $\mathcal{C}_7$ in scenario Sc-7, obtained from a fit of observable Set II of the $B \rightarrow K\pi$ system when treating $\rho _A^{K\pi }$ either as a fit parameter (*cyan*) or as a nuisance parameter (*red*)

We start the discussion of our results with the confrontation of our procedure of fitting simultaneously NP and WA parameters, with the conventional QCDF approach, where only NP parameters are fitted and WA parameters are treated as nuisance parameters. As an example, Fig. 9 provides the allowed regions of $\text{ Re }\,\mathcal{C}_7$ versus $\text{ Im }\,\mathcal{C}_7$ in the scenario Sc-7 from the observable Set II of the $B\rightarrow K\pi$ system. We emphasize again that both fits underly very different assumptions, in fact treating $\rho _A^{K\pi }$ as a nuisance parameter implies that it can be different for each decay as well as each observable, whereas fitting it imposes one universal parameter for all observables in the $B\rightarrow K\pi$ system. It can be seen that both approaches yield rather different results that overlap only for a very small part of the considered parameter space. The contour from conventional QCDF (red) allows $\text{ Im }\,\mathcal{C}_7$ to be rather large and even its sign is not dictated by the data. Contrary to that the corresponding contour that we obtain from a simultaneous fit of NP and WA parameters (cyan) becomes strongly constrained and fixes $\mathcal{C}_7$ to be almost purely real. The different outcomes due to the two treatments of $\rho _A$ originate from $r_\mathrm{{EW},7}^\mathrm{A}$, which is in both cases the leading NP contribution to ${\varDelta } C$ and $C(B^- \rightarrow \bar{K}^0 \pi ^-)$ in Eq. (5.4). However, for case (ii), $r_\mathrm{{EW},7}^\mathrm{A}$ is assigned with an approximately vanishing central value and huge symmetric uncertainties, whereas for (i) the central value of $r_\mathrm{{EW},7}^\mathrm{A}$ is large and uncertainties are small. The former implies that both CP asymmetries in Eq. (5.4) can be explained simultaneously due to large uncertainties, which depend linearly on $\text{ Im }\,\overline{\mathcal{C}}_7$ and enter the determination of the individual observables uncorrelated. The latter case, however, implies that a significant modification of one of the two CP asymmetries inevitably induces a similar large contribution to the other. Since $C(B^- \rightarrow \bar{K}^0 \pi ^-)$ is measured rather accurately and consistent with its value at the best-fit point of the SM fit, large contributions to $\text{ Im }\,\overline{\mathcal{C}}_7$ are consequently forbidden (see 4). This shows that the bounds on a NP parameter space strongly depend on the treatment of $\rho _A$.

**Fig. 10:**
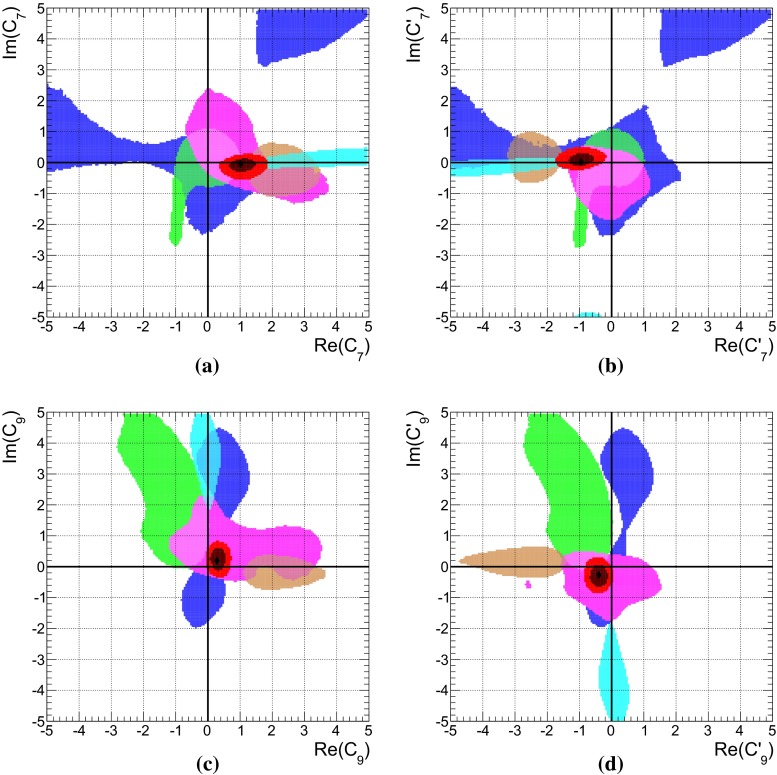
$68~\%$ CR for the complex Wilson coefficients $\mathcal {C}^{(\prime )}_{7,9}$ in the scenarios Sc-$7,7',9,9'$. Constraints are obtained from the decay systems $B \rightarrow K\pi$ (*cyan*), $B \rightarrow K\rho$ (*blue*), $B \rightarrow K^*\pi$ (*green*), $B \rightarrow K^*\rho$ (*purple*), and $B \rightarrow K^*\phi$ (*brown*). The combined contour (*red*) is shown for a probability of 68 and $95~\%$. The *filled diamond* corresponds to the best-fit point of the combined fit

Bounds on the complex-valued Wilson coefficients $\mathcal{C}^{(\prime )}_i$ from fits in scenarios of single operator dominance are shown in Fig. 10 for each of the decay systems $B \rightarrow K\pi ,\, K\rho ,\, K^*\pi ,\, K^*\rho ,\, K^*\phi$ at 68 % and their combination at 68 and 95 % probability. Due to the different dependence of the spin of the final states on chirality-flipped operators; see Eq. (5.1) and comments below, the contours for $B \rightarrow PP,\, V_L V_L$ systems are mirrored at the origin, whereas for $B \rightarrow PV$ systems they remain invariant, when considering scenarios that are related by $\mathcal{C}_i \leftrightarrow \mathcal{C}'_i$.

As can be expected from the pull values of the SM fit, shown in Table 5, the allowed regions from $B \rightarrow K\rho ,\, K^*\rho$ contain the SM, whereas some small pulls in $B \rightarrow K^*\pi$ can be reduced with non-SM values of $\mathcal{C}_{9,9'}$. Concerning $B \rightarrow K\pi$, the data prefers NP contributions that are almost purely real for Sc-$7,7'$ and imaginary for Sc-$9,9'$, excluding the SM with a probability of more than 95 %. As already explained above, experimental data of $C(B^- \rightarrow \bar{K}^0 \pi ^-)$ forbids large contributions to $\text{ Im }\,\overline{\mathcal{C}}_7$, implying also small ${\varDelta } C$ in the approximation of small $r_{i,j}$ as used in Eq. (5.4). Nevertheless, in our approach $r_{\mathrm {EW}, 7}^\mathrm{A}$ can become rather large, see Eq. (5.5), such that second order interference terms $\propto r_\mathrm{T}\, r_{\mathrm {EW}, 7}^\mathrm{A}\, \text{ Re }\, \overline{C}_7$, which do not exactly cancel in ${\varDelta } C$, can provide better agreement with the data. The improvement of the tension is quantified in Table 6 at the best-fit point of the combination of all five decay systems. For example, scenarios Sc-$7,7'$ allow one to reduce the pull of ${\varDelta } C$ of $-2.8 \sigma$ in the SM below $-1 \sigma$, and similarly for $R_n^B(K\pi )$. In scenarios Sc-$9,9'$ the solution to the “${\varDelta } {\mathcal{A}_\mathrm{CP}}$ puzzle” proceeds via $r_{\mathrm{EW},9}$, see Eq. (5.5), requiring large values of $\text{ Im }\, {\varDelta } \mathcal{C}_9$, which are strongly disfavored by measurements of direct CP asymmetries in $B \rightarrow K^* \phi$. In consequence of this strong tension, Sc-$9,9'$ cannot really improve existing pulls of the SM, except for $R_n^B(K\pi )$, which results in a very small improvement of ${\varDelta }\chi ^2(\mathrm{SM})$, shown in Table 6. Contrary, Sc-$7,7'$ exhibits a large improvement of ${\varDelta }\chi ^2(\mathrm{SM})$ since here the allowed region of the Wilson coefficient from $B \rightarrow K^* \phi$ is compatible with the one from $B \rightarrow K\pi$.

**Table 6:** Compilation of best-fit points and pull values with $|\delta | \ge 1.6$ for the model-independent fits of scenarios with NP in QED-penguin operators. $C(\bar{K}^{*0} \phi )$ and $\mathcal {B}(\bar{K}^{*0} \phi )$ are for experimental values [37]

	$\;\;\,\text{ Re }(\mathcal{C}_i^{(\prime )}),\, \text{ Im } (\mathcal{C}_i^{(\prime )})$	${\varDelta } C(K\pi )$	$R_n^B(K\pi )$	$C_L(K^{*-} \phi )$	$\mathcal {B}(\bar{K}^{*0} \phi )$	$f_L(K^{*-} \rho ^0)$	$R_n^B(K^{*}\pi )$	$R_n^B(K^{*}\rho )$	${\varDelta } \chi ^2(\mathrm SM)$
SM		$\mathbf {-2.8 \sigma }$	$\mathbf {-1.9 \sigma }$	$-1.5 \sigma$	$\mathbf {1.7/2.6 \sigma }$	$0.9 \sigma$	$0.6 \sigma$	$0.6 \sigma$	
Sc-7	$1.01,\, -0.04$	$-0.7 \sigma$	$-0.8 \sigma$	$\mathbf {-1.6 \sigma }$	$0.3/1.2 \sigma$	$1.0 \sigma$	$0.0 \sigma$	$1.1 \sigma$	18.7
Sc-$7'$	$-0.95,\,0.02$	$-0.8 \sigma$	$-0.8 \sigma$	$-\mathbf {1.6} \sigma$	$1.2/\mathbf {2.1} \sigma$	$1.2 \sigma$	$0.0\sigma$	$0.9 \sigma$	12.8
Sc-9	$0.28,\, 0.19$	$\mathbf {-2.7 \sigma }$	$0.0 \sigma$	$-1.3 \sigma$	$\mathbf {1.7/2.6 \sigma }$	$1.0\sigma$	$1.1 \sigma$	$0.7 \sigma$	1.5
Sc-$9'$	$-0.40,\, -0.27$	$\mathbf {-2.6 \sigma }$	$0.0 \sigma$	$-1.2 \sigma$	$\mathbf {1.6/2.5 \sigma }$	$1.1 \sigma$	$0.0\sigma$	$0.6 \sigma$	3.6
Sc-$77'$	$1.94,\,0.12$	$0.0 \sigma$	$0.0 \sigma$	$-1.5 \sigma$	$0.0/0.0 \sigma$	$0.8\sigma$	$0.3 \sigma$	$\mathbf {1.6 \sigma }$	23.9
	$$-1.65,\,0.03$$								
Sc-$99'$	$-0.05,\,2.15$	$-\mathbf {2.2} \sigma$	$0.0\sigma$	$-0.6 \sigma$	$\mathbf {1.6/2.5 \sigma }$	$0.9\sigma$	$\mathbf {1.6 \sigma }$	$0.7 \sigma$	9.7
	$$-0.42,\,1.64$$								
Sc-79	$1.02,\,-0.02$	$-0.9 \sigma$	$-0.6 \sigma$	$\mathbf {-1.6} \sigma$	$0.3/1.2 \sigma$	$0.9 \sigma$	$0.0 \sigma$	$1.2 \sigma$	19.0
	$$0.06,\, 0.13$$								
Sc-$7'9'$	$-1.75,\,-0.02$	$-0.4 \sigma$	$0.3 \sigma$	$\mathbf {-1.6} \sigma$	$0.0/0.3 \sigma$	$\mathbf {2.3} \sigma$	$1.0 \sigma$	$\mathbf {1.6} \sigma$	18.0
	$$-0.93,\,0.28$$								
Sc-$77'99'$	$1.61,\, 0.24$	$-0.1 \sigma$	$0.0 \sigma$	$-1.2 \sigma$	$0.0/0.0 \sigma$	$0.6 \sigma$	$0.7\sigma$	$1.5 \sigma$	31.2
	$$-0.87,\,0.11$$								
	$$0.31,\,1.65$$								
	$$-0.60,\,1.59$$								

Large pull values are in bold

**Table 7:** Predictions for the mixing-induced CP asymmetry of diverse $B_d$ decays and for the purely isospin-breaking branching ratios $\mathcal {B}(\bar{B}_s \rightarrow \phi \pi ,\, \phi \rho ) \times 10^6$ within the single dominant operator scenarios and the SM

	${\varDelta } S(K\pi )$	${\varDelta } S(K\rho )$	${\varDelta } S(K^*\pi )$	${\varDelta } S_L(K^*\rho )$	${\varDelta } S(K\eta ')$	${\varDelta } S(K\omega )$	${\varDelta } S(K\phi )$	${\varDelta } S_L(K^*\phi )$	$\mathcal {B}(\phi \pi )$	$\mathcal {B}(\phi \rho )$	$R_n^{B{_s}}(KK)$
HFAG	$$-0.11_{-0.17}^{+0.17} $$	$$-0.14_{-0.21}^{+0.18} $$	–	–	$$-0.05_{-0.06}^{+0.06} $$	$$ 0.03_{-0.21}^{+0.21} $$	$$0.06_{-0.13}^{+0.11} $$	–	–	–	–
SM	$$ [ 0.05,\, 0.13]$$	$$ [-0.19,\, -0.04]$$	$$ [ 0.06,\, 0.17]$$	$$ [-0.15,\, 0.09]$$	$$ [-0.01,\, 0.04]$$	$$ [ 0.09,\, 0.17]$$	$$ [0.01,\, 0.05]$$	$$ [ 0.01,\, 0.04]$$	$$ 0.24_{-0.04}^{+0.07}$$	$$0.68_{-0.10}^{+0.19}$$	$$ 0.99_{-0.08}^{+0.01}$$
Sc-7	$$ 0.13_{-0.13}^{+0.02}$$	$$-0.18_{-0.10}^{+0.11}$$	$$0.07_{-0.07}^{+0.09}$$	$$0.08_{-0.13}^{+0.07}$$	$$0.03_{-0.09}^{+0.04}$$	$$ 0.15_{-0.31}^{+0.04}$$	$$0.04_{-0.08}^{+0.05}$$	$$0.03_{-0.08}^{+0.06}$$	$$0.91_{-0.22}^{+0.28}$$	$$ 0.35_{-0.07}^{+0.14}$$	$$0.80_{-0.06}^{+0.04}$$
Sc-$$7'$$	$$ 0.13_{-0.13}^{+0.02}$$	$$-0.08_{-0.10}^{+0.07}$$	$$0.10_{-0.07}^{+0.09}$$	$$0.10_{-0.12}^{+0.09}$$	$$0.02_{-0.08}^{+0.06}$$	$$ 0.12_{-0.08}^{+0.06}$$	$$-0.01_{-0.07}^{+0.06}$$	$$0.05_{-0.07}^{+0.06}$$	$$0.06_{-0.04}^{+0.05}$$	$$ 0.26_{-0.08}^{+0.12}$$	$$0.87_{-0.06}^{+0.06}$$
Sc-9	$$ 0.06_{-0.08}^{+0.06}$$	$$ -0.04_{-0.10}^{+0.09}$$	$$0.05_{-0.09}^{+0.07}$$	$$-0.01_{-0.18}^{+0.10}$$	$$0.00_{-0.07}^{+0.06}$$	$$ 0.09_{-0.07}^{+0.09}$$	$$-0.04_{-0.07}^{+0.07}$$	$$-0.08_{-0.06}^{+0.11}$$	$$0.11_{-0.04}^{+0.06}$$	$$ 0.40_{-0.10}^{+0.13}$$	$$0.94_{-0.06}^{+0.03}$$
Sc-$$9'$$	$$ 0.05_{-0.08}^{+0.06}$$	$$-0.20_{-0.11}^{+0.10}$$	$$0.17_{-0.07}^{+0.05}$$	$$-0.05_{-0.15}^{+0.13}$$	$$-0.02_{-0.05}^{+0.08}$$	$$ 0.16_{-0.11}^{+0.03}$$	$$0.06_{-0.10}^{+0.03}$$	$$-0.03_{-0.12}^{+0.09}$$	$$0.49_{-0.12}^{+0.14}$$	$$ 0.32_{-0.10}^{+0.15}$$	$$0.93_{-0.04}^{+0.05}$$

**Fig. 11:**
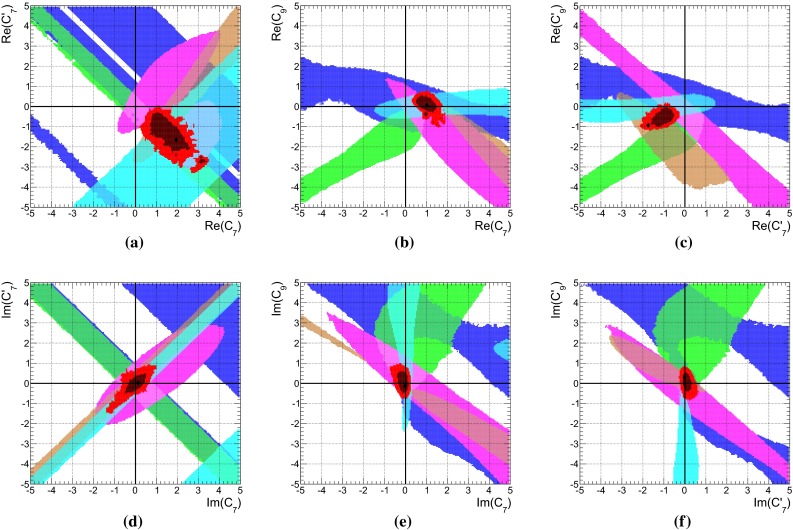
$68~\%$ CR for the complex Wilson Coefficients $\mathcal {C}^{(\prime )}_{7,9}$ in the scenarios Sc-$77'$ (*left*), Sc-79 (*middle*), and Sc-$7'9'$ (*right*). Constraints are obtained from the decay systems $B \rightarrow K\pi$ (*cyan*), $B \rightarrow K\rho$ (*blue*), $B \rightarrow K^*\pi$ (*green*), $B \rightarrow K^*\rho$ (*purple*), and $B \rightarrow K^*\phi$ (*brown*). The combined contour (*red*) is shown for 68 and $95~\%$ CRs. The *filled diamond* corresponds to the best-fit point of the combined fit

The analysis of scenarios that are dominated by single operators has shown that NP in QED-penguin operators is suitable to sufficiently address all tensions present in the SM, though not all in one particular scenario. The benefits of each single scenario combines in the generalized scenarios, as is evident from the improvement of ${\varDelta } \chi (\mathrm{SM})$ in Table 6. In fact, the most general considered Sc-$77'99'$ has greatly reduced pull values compared to the SM and largest ${\varDelta } \chi (\mathrm{SM})$. Concerning models that allow for NP in two Wilson coefficients, only Sc-$99'$ cannot resolve tensions in $B \rightarrow K\pi ,\, K^*\pi ,\, K^*\phi$, showing that NP is required in $\mathcal{C}_7$, respectively $\mathcal{C}'_7$. In Fig. 11, we show the contours for $\text{ Re }\, \mathcal{C}_i$ versus $\text{ Re }\, \mathcal{C}_j$ and $\text{ Im }\, \mathcal{C}_i$ versus $\text{ Im }\, \mathcal{C}_j$ of the fits of Sc-$77'$, Sc-79, and Sc-$7'9'$. The features of Fig. 10 are present again, namely large imaginary parts for the Wilson coefficients are excluded, whereas for $\mathcal{C}_7^{(\prime )}$ non-SM values for the real parts are allowed, disfavoring the SM by more than 95 % probability in all three scenarios. On the other hand large imaginary parts for $\mathcal{C}_9^{(\prime )}$ can only arise in Sc-$99'$ and Sc-$77'99'$, since only $\text{ Im }\, \overline{\mathcal{C}}_9$ is bound to be close to zero by the combination of $B \rightarrow K^*\rho ,\, K^* \phi$.

Measurements of the mixing-induced CP asymmetries ${\varDelta } S_f$ only exist for two out of the five considered decay systems: $\bar{B}^0 \rightarrow \bar{K}^0 \pi ^0$ and $\bar{B}^0 \rightarrow \bar{K}^0 \rho ^0$. Since these are rather imprecisely measured, we omit ${\varDelta } S_f$ as constraint from the fit and instead give predictions for each scenario of single operator dominance together with the SM prediction in Table 7. In the case of the SM, we observed that the mixing-induced CP asymmetries are insensitive to the residual $\rho _A$ parameter space that is allowed from constraints of branching fractions and direct CP asymmetries. As a consequence, the SM predictions are dictated by error estimation of the nuisance parameters and therefore quoted as interval. We have seen from the fits that CP-violating NP contributions to $\mathcal{C}^{(\prime )}_7$ are strongly disfavored and to $\mathcal{C}^{(\prime )}_9$ tightly constrained. Although $\text{ Im }\, \mathcal{C}^{\prime }_9$ could still become large if $\mathcal{C}_9$ and $\mathcal{C}^{\prime }_9$ are modified, such scenarios do not significantly increase the quality of the fit. Hence, mixing-induced CP asymmetries are not strongly affected in the case of single operator dominance and in most cases the central values of NP predictions coincide with the SM interval. Ratios of branching fractions, respectively branching fractions are more sensitive to, for example, large real-valued $\mathcal{C}^{(\prime )}_7$. In particular, the purely isospin-breaking branching fractions $B_s \rightarrow \phi \pi ,\, \phi \rho$ as well as $R_n^{B{_s}}(KK)$, which predictions are also accumulated in Table 6, are sensitive to NP in QED-penguin operators. Indeed, all four considered scenarios, except for the branching fraction of $B_s \rightarrow \phi \pi$ in Sc-7 and Sc-$9'$, predict a further suppression of $\mathcal{B}(B_s \rightarrow \phi \pi ,\,\phi \rho )$, which would unfortunately require even more experimental effort to observe these very rare decays. On the contrary, the prediction of $R_n^{B{_s}}(KK)$ remains unchanged within Sc-$9^{(\prime )}$, whereas it largely deviates within Sc-$7^{(\prime )}$ compared to the SM.

Apart from the NP parameters discussed so far, we simultaneously fitted one universal WA parameter per decay system. The comparison of the best-fit points of these parameters with the SM fit is summarized in Table 8 for each of the considered scenarios. These best-fit points lie in the solutions that were singled out by the SM fit, owing to the fact that NP in QED-penguin operators does not modify the numerically leading decay amplitude $\hat{\alpha }_4^c$. We further provide ranges for the ratios $\xi _3^A$ at 68 % probability that quantify the relative size of subleading WA amplitudes, which have been determined according to the procedure given in Sect. 3.2. The presence of NP always allows for smaller values of $\xi _3^A$ than in the SM fit. In the most general scenario Sc-$77'99'$ the size of power corrections can be lower than 15 % for all considered decay systems. Especially for $B \rightarrow K \rho ,\, K^*\rho$ also simpler NP scenarios already lead to a significant reduction. On the other hand the presence of NP might allow also for very large values of $\xi _3^A$ in most systems, except for $B \rightarrow K\pi \,(K^*\phi )$, where $\xi _3^A \lesssim 1.5\,(1.2)$.

**Table 8:** Compilation of best-fit points for $\rho _A$ and $\xi _3^A$ at $68~\%$ probability. The results are given for the considered decay systems and scenarios Sc-*i*. As explained in Sect. 3.2, the interval of $\xi _3^{A} (\mathrm{NP})$ should be compared to $\xi _3^{A}(\mathrm{SM})$ at the best-fit point of $\rho _A$, listed in the first row

	$K\pi$		$K^*\pi$		$K\rho$		$K^*\rho$		$K^*\phi$	
	$|\rho _A|,\, \phi _A$	$\xi _3^A$	$|\rho _A|,\, \phi _A$	$\xi _3^A$	$|\rho _A|,\, \phi _A$	$\xi _3^A$	$|\rho _A|,\, \phi _A$	$\xi _3^A$	$|\rho _A|,\, \phi _A$	$\xi _3^A$
SM	3.34, 2.71	0.39	1.61, 5.84	0.89	2.69, 2.68	0.78	1.56, 5.66	1.33	1.50, 2.82	0.38
Sc-7	2.14, 5.45	$[0.38,\, 0.60]$	1.80, 5.90	$[0.86,\,1.39]$	1.88, 5.58	$[0.39,\, 1.64]$	1.41, 5.66	$[0.70,\,1.81]$	1.53, 2.85	$[0.29,\, 0.65]$
Sc-$7'$	3.61, 2.68	$[0.34,\, 0.64]$	3.73, 1.84	$[0.72,\,2.72]$	2.14, 5.36	$[0.54 ,\, 1.46]$	1.29, 5.64	$[0.57,\,1.75]$	0.71, 5.64	$[0.38,\, 0.59]$
Sc-9	1.86, 5.49	$[0.35,\, 0.60]$	1.63, 5.87	$[0.78,\,1.49]$	1.52, 5.44	$[0.41,\, 1.38]$	1.54, 5.63	$[0.62,\,1.97]$	1.53, 2.83	$[0.35,\, 0.52]$
Sc-$9'$	1.85, 5.49	$[0.35,\, 0.62]$	2.99, 2.91	$[0.76,\,1.55]$	2.71, 2.68	$[0.41,\, 1.49]$	1.53, 5.62	$[0.62,\,1.91]$	1.55, 2.84	$[0.36,\, 0.53]$
Sc-$77'$	2.45, 5.69	$[0.34,\, 1.18]$	3.03, 2.91	$[0.71,\, 2.98]$	1.51, 5.44	$[0.00,\, 1.61]$	1.71, 6.00	$[0.51,\, 2.37]$	0.95, 6.00	$[0.30,\, 0.88]$
Sc-$99'$	2.39, 5.40	$[0.33,\, 0.71]$	3.34, 2.99	$[0.68,\, 3.24]$	1.44, 0.04	$[0.01,\, 2.78]$	2.31, 2.74	$[0.40,\, 2.28]$	1.54, 2.84	$[0.26,\, 0.71]$
Sc-79	3.59, 2.68	$[0.23,\,0.70]$	1.80, 5.90	$[0.78,\,1.70]$	1.95, 5.60	$[0.24,\, 2.40]$	2.19, 2.79	$[0.31,\,2.06]$	1.53, 2.86	$[0.17,\, 0.68]$
Sc-$7'9'$	2.17, 5.53	$[0.30,\, 0.66]$	2.89, 2.77	$[0.56,\, 2.74]$	2.19, 5.27	$[0.65,\, 1.84]$	2.43, 2.87	$[0.43,\, 1.92]$	0.99, 6.01	$[0.31,\, 0.83]$
Sc-$77'99'$	2.24, 5.56	$[0.08,\, 1.49]$	1.65, 6.07	$[0.11,\, 3.58]$	1.45, 6.28	$[0.00,\, 2.64]$	1.81, 5.89	$[0.12,\, 2.83]$	0.90, 5.93	$[0.01,\, 1.02]$

### NP in tree transitions $b\rightarrow s\, \bar{u}u$

In the case of the SM, isospin-breaking contributions to hadronic *B* decays occur either through QED-penguin operators, which were investigated in the previous section, or through tree-level operators with an up-quark current. The latter operators occur in the SM in a color-singlet, $O_1^u$, and -octet, $O_2^u$, configuration and are the only source of CP violation in the SM for flavor-violating $b\rightarrow s$ transitions of *B* mesons. Hence, these operators seem to be suitable to address the tensions of the SM in both ${\varDelta } C(K \pi )$ as well as $R_n^B(K\pi )$ if they can be enhanced. We also encountered some discrepancy in the branching fraction of $B \rightarrow K^{*0} \phi$, but these decays do not directly depend on either of the two tree-level operators, leaving their explanation, at least in the context of the following discussion, due to statistical fluctuation or underestimated theory uncertainties. Due to the strong CKM hierarchy in $b \rightarrow s$ transitions, $b \rightarrow s\, \bar{u} u$ operators give only numerically important contributions to CP asymmetries, contrary to $b \rightarrow d\, \bar{u} u$ operators, which are constrained by well-measured branching fractions and CP asymmetries in tree-dominated decays $B \rightarrow \pi \pi ,\, \rho \rho ,\, \rho \pi$ [21].

We introduce the following NP contribution to the Hamiltonian of Eq. (3.1):

5.6 $$\begin{aligned} C^u_{1,2}(\mu _0) = C_{1,2}^{u,\mathrm{SM}}(\mu _0) + \mathcal{C}^u_{1,2}, \end{aligned}$$

where we choose $\mu _0 = M_W$ as before. Although $\mathcal{C}^u_{1,2}$ mix into Wilson coefficients of all other SM operators, this contribution is doubly Cabibbo-suppressed compared to $\mathcal{C}^c_{1,2}$ and numerically negligible in all amplitudes, except for $r_\mathrm{T}^{},\, r_\mathrm{T}^\mathrm{C}$. As discussed in Eq. (4.5), in the SM the latter two are the dominant contributions in CP asymmetries for decay systems considered below.

In connection with the SM, we already discussed in Sect. 4.1 the possibility of a large hard-scattering solution to the ${\mathcal{A}_\mathrm{CP}}(K\pi )$ problem; see also [60]. Here we show that the assumption of NP in $b \rightarrow s\,\bar{u}u$ operators provide qualitatively different solutions to large hard scattering. For this purpose we recall the dependence of CP asymmetries and ratios of branching fractions Eq. (2.15) on the tree amplitudes:

5.7 $$\begin{aligned} C&\propto 2\, \text{ Im }\, (r^{\mathrm{(C)}}_{\mathrm{T}})\, \sin \gamma + 2\, \text{ Im }\, (r^{\mathrm{(C)}}_{\mathrm{T},j})\, \text{ Im }\, (\mathcal{C}_j e^{-i\gamma }), \nonumber \\ S&\propto 2\, \text{ Re }\, (r^{\mathrm{(C)}}_{\mathrm{T}})\, \sin \gamma + 2\, \text{ Re }\, (r^{\mathrm{(C)}}_{\mathrm{T},j})\, \text{ Im }\, (\mathcal{C}_j e^{-i\gamma }), \nonumber \\ R&\propto 2\, \text{ Re }\, (r^{\mathrm{(C)}}_{\mathrm{T}})\, \cos \gamma + 2\, \text{ Re }\, (r^{\mathrm{(C)}}_{\mathrm{T},j})\, \text{ Re }\, (\mathcal{C}_j e^{-i\gamma }) + \cdots , \end{aligned}$$

when utilizing the expansion in small $r_i$ and the dots stand for contributions of further $r_i$ that are not affected from NP in the considered scenarios. Hard scattering enters only the $r_i$, especially $r_\mathrm{T}^\mathrm{C}$. Hence, direct and mixing-induced CP asymmetries become correlated through their common dependence on $\text{ Im }\, (\mathcal{C}_j e^{-i\gamma })$, whereas they depend differently on hard scattering. Analogous, qualitative differences exist among CP asymmetries and the ratios *R*. In consequence, when mixing-induced CP asymmetries become more precisely measured, it will be possible to distinguish both scenarios.

We investigate the effects of the complex-valued Wilson coefficients $\mathcal{C}_j = |\mathcal{C}_j| e^{i\delta _j}$ separately and in combination in the three scenarios:

- Single operator dominance Sc-*i*: $\mathcal{C}^u_i \ne 0$ and $\mathcal{C}^u_{j \ne i} = 0$ for $i = 1,2$
- Combined scenario Sc-12: $\mathcal{C}^u_{1,2} \ne 0$

**Fig. 12:**
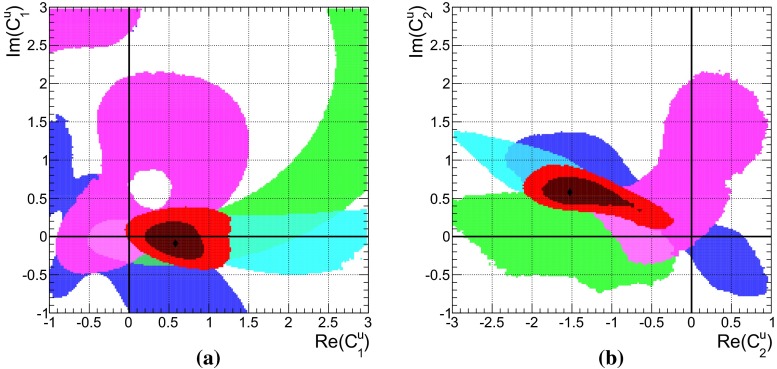
$68~\%$ CR for the complex Wilson coefficients $\mathcal {C}^{u}_{1,2}$ in the scenarios Sc-1 (*left*) and Sc-2 (*right*). Constraints are obtained from the decay systems $B \rightarrow K\pi$ (*cyan*), $B \rightarrow K\rho$ (*blue*), $B \rightarrow K^*\pi$ (*green*), and $B \rightarrow K^*\rho$ (*purple*). The combined contour (*red*) is shown for 68 and $95~\%$ CRs. The *filled diamond* corresponds to the best-fit point of the combined fit

Figure 12 shows the individual contours for $\mathcal{C}^u_{1}$ (left) and $\mathcal{C}^u_{2}$ (right) that were obtained from a fit of each decay systems $B \rightarrow K\pi ,\, K\rho ,\, K^*\pi ,\, K^*\rho$ within the scenarios of a single operator dominance. In the case of new-physics contribution to the color-singlet operator, the fit prefers a real-valued $\mathcal{C}^u_{1}$ with a significant contribution of the order of its SM value. Due to the parameterization of the effective weak Hamiltonian in Eq. (3.1) and of the NP contribution in Eq. (5.6), such a solution implies that the CP violating phase of a particular NP model has to be aligned with the one of the SM. Hence, the Wilson coefficient is enhanced from $C_1^u(M_W) = 0.98$ in the SM to $C_1^u(M_W) = |0.98 + (0.58 - i\, 0.09)| \approx 1.56$ at the best-fit point, tabulated in Table 9, whereas its weak phase $\gamma$ receives only marginal corrections from $\delta _1 \approx -8.8^\circ$. Since all contours from the individual decay systems nicely overlap with each other, we expect to resolve the discrepancy that are present for the SM in $B \rightarrow K \pi$ without introducing new tensions in the data of other decay systems. This is confirmed from the pull values listed in Table 9. It can also be seen from the table that the tensions in ${\varDelta } C(K\pi )$ and $R_n^B(K\pi )$ can be well explained within Sc-2 and Sc-12 when tolerating an increasing tension in $R_n^B(K^*\pi )$ of $1.6\sigma$, respectively $1.1\sigma$.

**Table 9:** Compilation of best-fit points and pull values, with $|\delta | \ge 1.6$, for the model-independent fits of $b\rightarrow s\,\bar{u}u$ operators

	$\text{ Re }(\mathcal{C}_i^u), \text{ Im } (\mathcal{C}_i^u)$	${\varDelta } C(K\pi )$	$R_n^B(K\pi )$	$R_n^B(K^*\pi )$	${\varDelta } \chi ^2(SM)$
SM		$\mathbf {-2.8} \sigma$	$\mathbf {-1.9} \sigma$	$0.6\sigma$	
Sc-1	$0.58,\, -0.09$	$-0.9 \sigma$	$0.0 \sigma$	$0.2\sigma$	9.2
Sc-2	$-1.53,\, 0.58$	$0.0 \sigma$	$-0.3 \sigma$	$\mathbf {1.6} \sigma$	12.4
Sc-12	$1.47,\,0.03$	$0.0 \sigma$	$-0.5 \sigma$	$1.1 \sigma$	15.6
	$$-2.25,\, 0.38$$

The corresponding contours of $\mathcal{C}_2^u$ are displayed in Fig. 12b. The combined contour reduces to a common area of the allowed regions for the decay systems $B \rightarrow K\pi ,\, K\rho ,\, K^*\rho$, whereas the green contour from $B \rightarrow K^*\pi$ is slightly separated from the combination. The SM value of the color-octet Wilson coefficient, $C_2^{u, \mathrm {SM}}(M_W) = 0.05$, is strongly suppressed compared to its color-singlet counterpart, but the preferred values that were obtained from our fits shift $C_2^u(M_W) = |0.05 + (-1.53 + i0.58)| \approx 1.58$ — competitive to $C_1^u(M_W)$. In contrast to Sc-1, the weak phase of $C_2^u$ is not aligned with the SM, but rather receives a significant phase shift of $\delta _2 \approx 159^\circ$.

The pattern that were obtained from the single operator dominance scenarios is also observed for the combined scenario: $C_{1,2}^u$ becomes further enhanced by $|0.98 + (1.47 + i0.03)| \approx 2.45$, respectively $|0.05 + (-2.25 + i0.38)| \approx 2.23$ and $\delta _1 \sim 1^\circ$, whereas $\delta _2$ further tend to $170^\circ$.

As in the previous analysis of the QED-penguin operators, we quote in Table 10 predictions for several mixing-induced CP asymmetries as well as for the isospin-sensitive branching fractions of $B_s \rightarrow \phi \pi ,\, \phi \rho$ and for $R_n^{B_s}(KK)$. The impact from an enhanced $\mathcal{C}_1^u$ on these observables is small and rather challenging to isolate from the SM background, which is not the case for NP in $\mathcal{C}_2^u$. Especially the predictions of the mixing-induced CP asymmetries of the decays $B \rightarrow K\pi ,\, K\rho ,\, K^*\pi ,\, K^*\rho$ and $B \rightarrow K\omega$ are visibly different compared to the SM, making these observables an ideal probe of NP in the color-octet operator. The same is true for the branching fractions of $B_s \rightarrow \phi \pi ,\,\phi \rho$, which we found to be enhanced by a factor of 5–6 for Sc-2 and by more than a factor of 10 in the case of Sc-12. Although these predictions largely deviate from the one of the SM, existing measurements do not contradict NP in $\mathcal{C}_2^u$ due to lacking precision.

**Table 10:** Predictions for the mixing-induced CP asymmetry of diverse $B_d$ decays and for the purely isospin-breaking branching ratios $\mathcal {B}(\bar{B}_s \rightarrow \phi \pi ,\, \phi \rho )$ $\times 10^6$ within the Sc-1, 2 scenarios and the SM

	${\varDelta } S(K\pi )$	${\varDelta } S(K\rho )$	${\varDelta } S(K^*\pi )$	${\varDelta } S_L(K^*\rho )$	${\varDelta } S(K\eta ')$	${\varDelta } S(K\omega )$	${\varDelta } S(K\phi )$	${\varDelta } S_L(K^*\phi )$	$\mathcal {B}(\phi \pi )$	$\mathcal {B}(\phi \rho )$	$R_n^{B_s}(KK)$
HFAG	$$-0.11_{-0.17}^{+0.17} $$	$$-0.14_{-0.21}^{+0.18} $$	–	–	$$-0.05_{-0.06}^{+0.06} $$	$$ 0.03_{-0.21}^{+0.21} $$	$$0.06_{-0.13}^{+0.11} $$	–	–	–	–
SM	$$ [ 0.05,\, 0.13]$$	$$ [-0.19,\, -0.04]$$	$$ [ 0.06,\, 0.17]$$	$$ [-0.15,\, 0.09]$$	$$ [-0.01,\, 0.04]$$	$$ [ 0.09,\, 0.17]$$	$$ [ 0.01,\, 0.05]$$	$$ [ 0.01,\, 0.04]$$	$$ 0.24_{-0.04}^{+0.07}$$	$$0.68_{-0.10}^{+0.19}$$	$$ 0.99_{-0.08}^{+0.01}$$
Sc-1	$$ 0.13^{+0.07}_{-0.07}$$	$$ -0.10^{+0.06}_{-0.22}$$	$$0.12^{+0.11}_{-0.06}$$	$$ -0.06^{+0.16}_{-0.32}$$	$$0.01^{+0.07}_{-0.06}$$	$$ 0.20^{+0.06}_{-0.09}$$	$$0.09^{+0.02}_{-0.11}$$	$$ 0.09^{+0.02}_{-0.11}$$	$$0.27^{+0.06}_{-0.07}$$	$$ 0.73^{+0.17}_{-0.17}$$	$$0.92_{-0.09}^{+0.08}$$
Sc-2	$$ -0.30_{-0.12}^{+0.11}$$	$$ 0.26_{-0.09}^{+0.01}$$	$$-0.66_{-0.19}^{+0.20}$$	$$ -1.05_{-0.28}^{+0.30}$$	$$0.06_{-0.05}^{+0.05}$$	$$ -0.55_{-0.22}^{+0.25}$$	$$0.09_{-0.11}^{+0.02}$$	$$ 0.09_{-0.11}^{+0.01}$$	$$1.36_{-0.54}^{+0.74}$$	$$ 3.94_{-1.40}^{+1.77}$$	$$0.91_{-0.03}^{+0.04}$$
Sc-12	$$ -0.42_{-0.20}^{+0.15}$$	$$ 0.18_{-0.25}^{+0.09}$$	$$-0.93_{-0.29}^{+0.29}$$	$$ -1.59_{-0.11}^{+0.43}$$	$$0.07_{-0.05}^{+0.06}$$	$$ -0.51_{-0.43}^{+0.21}$$	$$0.06_{-0.06}^{+0.05}$$	$$ 0.04_{-0.04}^{+0.06}$$	$$2.74_{-1.21}^{+1.59}$$	$$ 8.78_{-3.67}^{+3.74}$$	$$0.85_{-0.09}^{+0.11}$$

As before, the NP contributions to the Wilson coefficients have been fitted simultaneously with WA parameters $\rho _A$ for each decay system in all considered scenarios. Since NP in $b \rightarrow s\,\bar{u}u$ operators do not contribute directly to the leading decay amplitude $\hat{\alpha }_4^c$ but rather indirectly through the common dependence on the likelihood function, we expect moderate changes of WA compared to the results of the SM fit. The best-fit points of the individual $\rho _A$ as well as the 68 % probability intervals of $\xi _3^A$ are summarized in Table 11 for each of the three scenarios. We observe that almost all best-fit points of $\rho _A$ lie within the contour regions of the SM fit. The only exceptions are $\rho _A^{K\rho }$ in Sc-12 and $\rho _A^{K\pi }$ for all considered scenarios. For the latter, the most likely values of $|\rho _A^{K\pi }|$ in the case of Sc-1 and Sc-2 are significantly reduced compared to the SM, whereas $\phi _A^{K\pi }$ tends toward smaller strong phases in the combined scenario. Due to the additional degrees of freedom, it is possible that the relative amount of power-suppressed corrections can be reduced. In general $\xi _3^A$ is most strongly affected in the combined scenario, for which we find lower bounds on $\xi _3^A(K\pi ) \gtrsim 0.13$, $\xi _3^A(K\rho ) \gtrsim 0.22$, and $\xi _3^A(K^*\rho ) \gtrsim 0.27$. The potential suppression of $\xi _3^A$ for $B \rightarrow K^*\pi$ is less effective and a relative amount of power-suppressed contribution of at least 0.54 is required in any case. It is worth to notice that the large WA scenario is still disfavored for $B \rightarrow K \pi$, which is in general not true for all other decay modes.

**Table 11:** Compilation of best-fit points for $\rho _A$ and $\xi _3^A$ at $68~\%$ probability. The results are given for the considered decay systems and scenarios Sc-*i*. As explained in Sect. 3.2, the interval of $\xi _3^{A} (\mathrm{NP})$ should be compared to $\xi _3^{A}(\mathrm{SM})$ at the best-fit point of $\rho _A$, listed in the first row

	$K\pi$		$K^*\pi$		$K\rho$		$K^*\rho$	
	$\rho _A$	$\xi _3^A$	$\rho _A$	$\xi _3^A$	$\rho _A$	$\xi _3^A$	$\rho _A$	$\xi _3^A$
SM	3.34, 2.71	0.39	1.61, 5.84	0.89	2.69, 2.68	0.78	1.56, 5.66	1.33
Sc-1	$1.54,\, 5.58$	$[0.29,\, 0.50]$	$1.65,\, 5.87$	$[0.73,\, 1.33]$	$3.03,\, 2.81$	$[0.53,\, 1.32]$	$1.39,\,5.90$	$[0.83,\, 1.72]$
Sc-2	$1.54,\, 5.58$	$[0.26,\,0.77]$	$1.60,\, 5.92$	$[0.75,\,1.33]$	$1.11,\, 0.19$	$[0.33,\,1.00]$	$2.63,\, 3.76$	$[0.92,\,2.32]$
Sc-12	$2.05,\, 5.80$	$[0.13,\,0.88]$	$1.52,\, 5.90$	$[0.54,\,1.47]$	$3.76,\, 4.56$	$[0.22,\,3.17]$	$1.96,\, 3.38$	$[0.27,\,2.61]$

## Conclusion

In this work we have carried out a phenomenological study of QCD- and QED-penguin-dominated charmless 2-body *B*-meson decays in the framework of QCD factorization (QCDF). In particular we investigated whether data supports the assumption of one universal parameter, $\rho _A$, in weak-annihilation (WA) contributions for decay channels related by $(u\leftrightarrow d)$ quark exchange in $B_{u,d,s}$ meson decays to *PP*, *VP*, and *VV* final states, while the remaining theory uncertainties are incorporated in an uncorrelated manner.

We analyze the decay systems of $$B_{u,d}$$ decays into $$PP = K\pi ,\, K\eta ^{(')},\, KK$$ or $$PV = K\rho ,\, K\phi ,\, K\omega ,\, K^*\pi ,$$$$K^*\eta ^{(')}$$ or $$VV = K^*\rho ,\, K^*\phi ,\, K^*\omega ,\, K^*K^*$$, and further $$B_s$$ decays into $$PP = \pi \pi ,\, KK,\, K\pi $$ or $$VV = \phi \phi ,\, K^*\phi ,$$$$K^* K^*$$ final states and employ the available data (see Tables 1, 2, 3) on branching fractions, direct CP asymmetries and for *VV* final states also polarization fractions and relative phases between polarization amplitudes.

Within the standard model (SM), the data can be described using one universal WA parameter for each decay system. The only exception is the $$B\rightarrow K\pi $$ system when using Set II of observables, as specified in Sect. 2.2, which includes $${\varDelta } {\mathcal{A}_\mathrm{CP}}$$ and $$R_n^B$$, as a manifestation of the “$${\varDelta } {\mathcal{A}_\mathrm{CP}}$$ puzzle” in our framework. The only other noticeable pull value of $$2.6\sigma $$$$(1.7\sigma )$$ arises for the measurement of $$\mathcal{B}(\bar{B}^0\rightarrow \bar{K}^{*0}\phi )$$ from Belle (BaBar). For each system, there are at least two allowed regions at 68 % CR with the best-fit solution residing in one of them (see Table 4). These two regions correspond to phases close to $$\pi $$ and $$2\pi $$, outside of regions of large destructive interference of WA amplitudes with leading amplitudes. Moreover, the ratio of the magnitudes of WA amplitudes to leading amplitudes, $$\xi _3^A$$ (see Table 4), is similar in size in both regions and within the 68 % CR it is possible to have $$\xi _3^A < 1$$ (except for $$B_s \rightarrow K^* K^*$$) and for the majority even $$\xi _3^A < 0.5$$. The current data is thus described by the employed approximations in the $$1/m_b$$ expansion without the need of anomalously large WA contributions.

We emphasize that in our analysis the “${\varDelta } {\mathcal{A}_\mathrm{CP}}$ puzzle” is only present if we assume a universal WA parameter that can be fitted from data. If we lift this assumption the anomaly would only reappear if we restrict our analysis to rather small WA parameters $\rho _A$. Without such a restriction, however, the non-linear dependence of $\xi _3^A$ on $\rho _A$ still permits reasonably small $\xi _3^A$, which are not larger as currently accepted in the literature.

We studied also ratios of branching fractions and differences of CP asymmetries (Set II) for the decay systems $B\rightarrow K\pi ,\, K^*\pi ,\, K\rho ,\, K^*\rho$. They are less sensitive to form factor and CKM uncertainties or are especially sensitive to numerically suppressed contributions from tree topologies. The according results listed in Table 5 show that currently both sets yield good fits to the data, except for $B\rightarrow K\pi$, where Set II has a *p*-value of only 4 %. The data of ratios of branching fractions and differences of CP asymmetries have been obtained by ourselves from measurements of observables in Set I. This neglects correlations and potential cancelations of systematic uncertainties accessible only in the experimental analyses. In this regard, future analysis would benefit from the direct experimental determination of these composed observables.

In view of the large pull value of $2.8\sigma$ for ${\varDelta } {\mathcal{A}_\mathrm{CP}}$ in $B\rightarrow K\pi$, we performed also a simultaneous fit of the WA and hard-scattering (HS) phenomenological parameters in the SM. The HS contribution necessary to lower the pull value of ${\varDelta } {\mathcal{A}_\mathrm{CP}}$ to $1.0\sigma$ is not larger than typically considered in conventional error estimates in the literature — $\xi _2^H = 1.0$. A better description of the data can be achieved with even larger HS contributions. A preciser measurement of $C(B_d \rightarrow K^0 \pi ^0)$ and $S(B_d \rightarrow K^0 \pi ^0)$ in the future could be helpful to test a “large HS”-scenario. Further, larger HS contributions allow for smaller WA contributions.

We investigate the feasibility to constrain new-physics (NP) scenarios in view of the aforementioned tensions in the SM. Within our framework this requires the fit of WA phenomenological parameters simultaneously with NP parameters from data. In contrast to the conventional handling of WA contributions within QCDF, we find that the assumption of one universal parameter per decay system yields stronger constraints on new-physics parameters for the considered scenarios. We have studied model-independent scenarios of NP in QED-penguin operators as possible solutions to the “${\varDelta } {\mathcal{A}_\mathrm{CP}}$ puzzle” in $B\rightarrow K \pi$ and tensions in $B\rightarrow K^*\phi$, taking into account also data from the systems $B\rightarrow K\rho ,\, K^*\pi ,\, K^*\rho$. As a second possible solution to the “${\varDelta } {\mathcal{A}_\mathrm{CP}}$ puzzle” we investigated NP in $b\rightarrow s\,\bar{u}u$ current-current operators including again data from $B\rightarrow K\rho ,\, K^*\pi ,\, K^*\rho$. For each scenario we provide the best-fit regions of the NP contributions to the according Wilson coefficients, reduction of $\chi ^2$ compared to the SM fit, the pull values of observables, and predictions of mixing-induced CP asymmetries, as well as branching fractions of $B_s \rightarrow \phi \pi ,\, \phi \rho$.

In both classes of NP scenarios there is no direct contribution to the numerically leading amplitude of QCD-penguin operators, since we consider only new isospin-violating contributions. In consequence, the allowed regions of WA parameters do not differ qualitatively from those of the SM fit. Yet, the combined fit of NP and WA allows for smaller $\xi _3^A$ in all scenarios compared to the SM.

It is conceivable that one day factorization theorems will be established even for WA contributions involving then new nonperturbative quantities. Our studies suggest that it will be possible to extract these new quantities also from data in the lack of first-principle nonperturbative methods of their calculation. It will be important to have access to more accurate measurements of the involved observables which should become available from Belle II and LHCb within the next decade.

## Appendix A: Numerical input

Here we collect the numerical input used in our analysis in Table 12. We list two sets of CKM parameters. The first, denoted by “SM”, is obtained in a global CKM fit in the framework of the SM [73] and is used throughout our SM analyses; see Sect. 4. The second, denoted by “NP”, is obtained from a global fit that includes only tree-level mediated observables [73] and which we use throughout the analysis of scenarios beyond the SM in Sect. 5. Further, for $B_s$ decays we use the value of $y_s$ Eq. (2.10) as an additional source of error in Eq. (2.4). Throughout we vary the renormalization scale $\mu _b \in [m_b/2,\, 2 m_b]$ for the central value of $\mu _b = 4.2$ GeV. The uncertainty from endpoint divergences in subleading hard-scattering contributions is determined by varying $\phi _H \in [0,\, 2\pi ]$ for fixed $|\rho _H| = 1$, as frequently done in the literature.

**Table 12:** Numerical input used for our analysis. Form factors are given at zero momentum transfer $q^2 = 0$. Other scale dependent quantities are quoted at the scale $\mu = 2\, \mathrm GeV$. $^\dagger$For the $B \rightarrow K$ form factor we used $\alpha _4^K(2.2 \, \mathrm GeV) = -0.0089$  [78] as additional input

Electroweak input					
$G_F [10^{-5}\, \mathrm GeV^{-2}]$	${\varLambda }_{\overline{\mathrm{MS}}}^{(5)}[\, \mathrm GeV]$	$M_Z [\, \mathrm GeV]$	$\alpha _s^{(5)}(M_Z)$	$\alpha _e^{(5)}(m_b)$	
1.16638	0.213	91.1876	0.1184	1 / 132	[74, 75]

Quark masses [GeV]					
$m_t^\mathrm{pole}$	$m_b(m_b)$	$m_c(m_b)$	$m_s$	$m_q/m_s$	
$(173.2 \pm 0.9)$	4.2	$(1.3 \pm 0.2)$	$(0.095 \pm 0.005)$	0.0370	[5, 74, 76]

CKM elements					
	$\lambda$	$|V_{cb}|$	$\bar{\rho }$	$\bar{\eta }$	
SM	$0.22535 \pm 0.00065$	$0.04172 \pm 0.00056$	$0.127 \pm 0.023$	$0.353 \pm 0.014$	[73]
NP	$0.2253 \pm 0.0006$	$0.04061 \pm 0.00097$	$0.147 \pm 0.045$	$0.368 \pm 0.048$	

B-meson input					
	$B_u$	$B_d$	$B_s$		
$f_B[\, \mathrm MeV]$	$190.5 \pm 4.2$		$227.7 \pm 4.5$		
$\lambda _B[\, \mathrm MeV]$	$200^{+250}_{-0}$				[77]
$\tau _B$[ps$^{-1}$]	1.641	1.519	1.516		[74]
$M_B[\, \mathrm MeV]$	5279.25	5279.58	5366.77		

Hadronic input—pseudoscalar mesons					
	*K*	$\pi$	$\eta$	$\eta ^{\prime }$	
$f_P[\, \mathrm MeV]$	160	131	$(1.07 \pm 0.02) f_{\pi }$	$(1.34\pm 0.06) f_{\pi }$	[4, 5]
$F_0^{B \rightarrow P}$	$0.33 \pm 0.04^\dagger$	$0.26 \pm 0.02$	$0.23 \pm 0.05$	$0.19 \pm 0.12$	[4, 78, 79]
$F^{B_s \rightarrow P}$	$0.30^{+0.04}_{-0.03}$	–	–	–	[80]
$\alpha _1(P)$	$0.05 \pm 0.02$	0.00	0.00	0.00	[4, 81]
$\alpha _2(P)$	$0.17 \pm 0.10$	$0.17 \pm 0.10$	$0.00 \pm 0.3$	$0.00 \pm 0.3$	

Hadronic input – vector mesons					
	$K^*$	$\rho$	$\phi$	$\omega$	
$f_V[\, \mathrm MeV]$	$218 \pm 4$	$209 \pm 1$	$221 \pm 3$	$187 \pm 3$	[5]
$f_V^\perp [\, \mathrm MeV]$	$175 \pm 10$	$156 \pm 9$	$175 \pm 9$	$142 \pm 9$	[82]
$A_0^{B \rightarrow V}$	$0.34 \pm 0.03$	$0.30 \pm 0.03$	–	$0.28\pm 0.03$	[83]
$F_-^{B \rightarrow V}$	$0.62 \pm 0.05$	$0.58 \pm 0.04$	–	$0.55\pm 0.04$	
$F_+^{B \rightarrow V}$	$0.00 \pm 0.06$	$0.00 \pm 0.06$	–	$0.00\pm 0.06$	[23]
$A_0^{B_s \rightarrow V}$	$0.39 \pm 0.03$	–	$0.47 \pm 0.04$	–	[83]
$F_-^{B_s \rightarrow V}$	$0.59 \pm 0.04$	–	$0.72 \pm 0.04$	–	
$F_+^{B_s \rightarrow V}$	$0.00 \pm 0.06$	–	$0.00 \pm 0.06$	–	[23]
$\alpha _1(V)$	$0.02 \pm 0.02$	0.00	0.00	0.00	[84]
$\alpha ^\perp _1(V)$	$0.03 \pm 0.03$	0.00	0.00	0.00	
$\alpha _2(V)$	$0.08 \pm 0.06$	$0.10 \pm 0.05$	$0.13 \pm 0.06$	$0.10 \pm 0.05$	
$\alpha ^\perp _2(V)$	$0.08 \pm 0.06$	$0.11 \pm 0.05$	$0.11\pm 0.05$	$0.11 \pm 0.05$	

## Appendix B: Statistical procedure

This appendix summarizes the statistical methods that are used in order to obtain probability regions for the parameters of interest, pull values of theory predictions and corresponding measurements of observables, and *p* values as a measure of the goodness of fit. Further, we describe the determination of probability distributions of predictions for observables that were not included in the fit.

### Appendix B.1: Probability regions

For the purpose of parameter inference we use Bayes’ theorem to determine the posterior probability distribution, $P(\varvec{\theta }|M,D)$, of the parameters of interest, $\varvec{\theta }= (\theta _1, \theta _2, \ldots )$, given a model *M* and data *D*. Parameters of interest in our analysis are (1) the phenomenological parameters of weak annihilation $\rho _A^{M_1M_2}$ and (2) parameters of new physics scenarios. Bayes’ theorem relates the posterior probability to the likelihood $\mathcal{L} (\varvec{\theta }) = P(D|M, \varvec{\theta })$, which is the probability of the data given the model *M* with parameter values $\varvec{\theta }$ and the prior distributions, $P(M, \varvec{\theta })$, which are the probability of model *M* with parameter values $\varvec{\theta }$

B1 $$\begin{aligned} P(\varvec{\theta }|M, D)&= \frac{P(D|M, \varvec{\theta }) \, P(M, \varvec{\theta })}{Z}. \end{aligned}$$

Here, the model-dependent normalization factor

B2 $$\begin{aligned} Z&\equiv \int \!\! P(D|M, \varvec{\theta }) \, P(M, \varvec{\theta }) \, \mathrm{d}\varvec{\theta }\end{aligned}$$

is known as “evidence” or “marginal likelihood” that plays an important role in model comparison within the Bayesian approach. Throughout, the priors of the $\varvec{\theta }$ are chosen as uniform within a certain interval.

It is common to introduce the likelihood function $\mathcal{L} (\varvec{\theta })$ as the product of the probabilities $p(O_i = O_i^\mathrm{th}(\varvec{\theta }))$ that each observable $O_i$ in the data set takes the particular value $O_i^\mathrm{th}(\varvec{\theta })$ predicted at the value of $\varvec{\theta }$,

B3 $$\begin{aligned} \mathcal{L} (\varvec{\theta })&= \prod _{i\, \in \, \mathrm{data}} p \Big (O_i = O_i^\mathrm{th}(\varvec{\theta })\Big ) \nonumber \\&\sim \exp \left[ -\frac{1}{2}\sum _{i \, \in \, \mathrm{data}} \Big (\chi _i(\varvec{\theta })\Big )^2 \right] . \end{aligned}$$

The probabilities *p* are given by the measured probability density functions pdf$[O_i]$ of each observable $O_i$ and the second part $\sim$ of (B3) indicates the special case of gaussian distributed pdf’s permitting to define a $\chi _i(\varvec{\theta })$.

The expression of $\mathcal{L} (\varvec{\theta })$ does not yet include the uncertainties due to nuisance parameters, $\varvec{\nu }= (\nu _1, \nu _2, \ldots )$, which enter theoretical predictions of $B\rightarrow M_1 M_2$ decays. In this case, the nuisance parameters give rise to an interval for the theory prediction $\big [O_i^\mathrm{th} - {\varDelta }^-_i,\, O_i^\mathrm{th} + {\varDelta }^+_i\big ]$ with possibly asymmetric uncertainties ${\varDelta }^\pm _i$ around the central value $O_i^\mathrm{th}$, which is obtained for central values of all nuisance parameters. Here, the theoretical uncertainty ${\varDelta }^\pm _i$ is determined by adding in quadrature the uncertainties due to each nuisance parameter $\nu _a$,

B4 $$\begin{aligned} {\varDelta }^\pm _i&= \sqrt{ \, \sum _a \left( {\varDelta }^\pm _{i,a}\right) ^2} , \end{aligned}$$

which arises from the minimal, central and maximal values $\nu _a^\mathrm{min}$, $\nu _a^\mathrm{cen}$ and $\nu _a^\mathrm{max}$, respectively,

B5 $$\begin{aligned} {\varDelta }^{+(-)}_{i,a}&= \big |O_i^\mathrm{th}(\nu _a^\mathrm{max(min)}) - O_i^\mathrm{th}(\nu _a^\mathrm{cen})\big | , \end{aligned}$$

while keeping all others at their central values. Clearly, this is an approximation that neglects more complicated interdependences of observables on several parameters and also possible correlations among different nuisance parameters. The nuisance parameters are listed in Table 12.

In the presence of nuisance parameters, we will adopt the simple procedure to use the maximal value of the pdf inside the interval of the theory prediction, hence replacing in (B3)

B6 $$\begin{aligned}&\text{ pdf } \Big (O_i = O_i^\mathrm{th}(\varvec{\theta })\Big ) \rightarrow \nonumber \\&\quad \max \Big ( \text{ pdf } (O_i) \, \big | \, O_i \in [O_i^\mathrm{th} - {\varDelta }^-_i,\, O_i^\mathrm{th} + {\varDelta }^+_i] \Big ) , \end{aligned}$$

where the dependence of $O_i^\mathrm{th}$ and ${\varDelta }^\pm _i$ on $\varvec{\theta }$ and $\varvec{\nu }$ is not explicitly shown. This procedure is implemented easily for gaussian distributed pdf’s by the modification of the definition of

B7 $$\begin{aligned} \chi _i(\varvec{\theta }, \varvec{\nu })&\!=\! \left\{ \begin{array}{ccc} \displaystyle \frac{\big |O_i^\mathrm{th}(\varvec{\theta },\varvec{\nu }) - O_i^\mathrm{exp}\big | - {\varDelta }^+_i(\varvec{\theta },\varvec{\nu })}{\sigma ^-_i} &{} \text{ if } &{} O_i^\mathrm{exp} \ge O_i^\mathrm{th} + {\varDelta }^+_i \\ \displaystyle \frac{\big |O_i^\mathrm{th}(\varvec{\theta },\varvec{\nu }) - O_i^\mathrm{exp}\big | - {\varDelta }^-_i(\varvec{\theta },\varvec{\nu })}{\sigma ^+_i} &{} \text{ if } &{} O_i^\mathrm{exp} \le O_i^\mathrm{th} - {\varDelta }^-_i \\ 0 &{} \,\,\,\,\text{ else } &{} \end{array} \right. \end{aligned}$$

where $O_i^\mathrm{exp}$ and $\sigma ^\pm _i$ denote the central value and the left and right standard deviation of the pdf$[O_i]$, respectively. The central value of the theoretical prediction $O_i^\mathrm{th}$ is obtained at the particular value of the parameters of interest $\varvec{\theta }$, and the $\varvec{\nu }$ are set to their central values.

Obviously, the modification (B7) is tailored to gaussian pdf’s, which is our interpretation of experimental world averages given by the Particle Data Group (PDG) [74] or HFAG [19]. However, the ratios of gaussian distributed observables—like the ones defined in Eq. (2.15): $R = \mathcal{B}_1/\mathcal{B}_2$—follow a gaussian ratio distribution. In the absence of experimental results of these ratios, one has to resort to the combination of the two gaussian distributions of numerator and denominator. In all relevant cases, the $\mathcal{B}_i$ are gaussian distributed with symmetric errors (from HFAG) and assuming that their errors are uncorrelated, the analytical expression of *p*(*R*) is well known [85]. Since it is monotonously increasing till its maximum at $R^\mathrm{exp} \equiv \mathcal{B}_1^\mathrm{exp}/\mathcal{B}_2^\mathrm{exp}$ and then monotonously falling, the maximal value of the probability in the theory interval can be easily found by evaluating *p*(*R*) at

B8 $$\begin{aligned} R&= \left\{ \begin{array}{lll} \displaystyle R_i^\mathrm{th} + {\varDelta }^+_i &{}\quad \text{ if } &{} R^\mathrm{exp} \ge R_i^\mathrm{th} + {\varDelta }^+_i \\ R_i^\mathrm{th} - {\varDelta }^-_i &{}\quad \text{ if } &{} R^\mathrm{exp} \le R_i^\mathrm{th} - {\varDelta }^-_i . \\ \displaystyle R^\mathrm{exp} &{}\,\,\,\, \text{ else } &{} \end{array} \right. \end{aligned}$$

The probability value is converted to $\chi = -2 \log p(R)$. Let us finally note that the difference between the gaussian ratio distribution and a gaussian distribution with central value *R* and $\sigma (R)$ determined from simple uncertainty propagation calculus, is numerically negligible unless large deviations of experimental and theoretical values probe the tails of the distributions, which are “heavier” for the gaussian ratio distribution.

Concerning the evaluation of the posterior probability, it is determined numerically with the help of the Markov Chain Monte Carlo (MCMC) implementation of the Bayesian Analysis Tool (BAT) [72]. One- and two-dimensional posterior distributions are obtained in turn by marginalization over the remaining parameters of interest. The best-fit points are identified with the help of Minuit that is initialized with the point of the highest posterior found during the MCMC run.

### Appendix B.2: Pull value

The deviation of a single measurement of an observable $O_{i}$ from its prediction $O_i^\mathrm{th} \pm {\varDelta }^\pm _i$ for a particular value of the parameters of interest $\varvec{\theta }_{\varvec{*}}$ will be given in terms of the pull value, accounting for theoretical uncertainties. Here, we define the pull value, $\delta$, as the integral over those regions of the pdf [$O_{i}$] that have higher probability than the maximal probability value $p^\mathrm{max}$ appearing in the interval spanned by the theory prediction $[O_i^\mathrm{th} - {\varDelta }^-_i,\, O_i^\mathrm{th} + {\varDelta }^+_i]$ evaluated at $\varvec{\theta }_{\varvec{*}}$ and varying the nuisance parameters, i.e.,

B9 $$\begin{aligned} \delta&= \int _{-\infty }^{+\infty } \mathrm{d} O_i\, p(O_i)\, \theta \big [p(O_i) - p^\mathrm{max}\big ] \end{aligned}$$

where $\theta (x)$ denotes the step function. Consequently, the pull value is zero if the maximum of the pdf is inside this theory interval. In the case of a normally distributed pdf (with $\sigma ^{+}_i = \sigma ^{-}_i$), a nonzero pull implies a symmetric integration interval around the central value $O_i^\mathrm{exp}$ of the distribution and the integrated fraction of probability can be converted into the distance between the lower or upper boundary of the theory uncertainty interval to $O_i^\mathrm{exp}$ in terms of its standard deviation $\sigma _i$ depending on whether the theory prediction is above or below $O_i^\mathrm{exp}$. In the case of non-gaussian pdf’s, the pull value gives a measure of the probability fraction that corresponds to those values of $O_i$ that have higher experimental probability than the ones contained in the interval of the theory prediction.^5^ The pull value is simply calculated by drawing values for $O_i$ that are distributed according to the pdf [$O_i$] and taking the ratio of the cases in which $p(O_i) > p^\mathrm{max}$ and the total number of draws.

### Appendix B.3: *p* Value

As a measure of the goodness of fit, we will use *p* values in order to compare within the same theoretical model at some point $\varvec{\theta }_{\varvec{*}}$—usually the best-fit point(s)—the quality of the fit for different sets of data Set I and Set II. For this purpose we will assume the model with the specific choice $\varvec{\theta }_{\varvec{*}}$, allowing us to produce frequencies of possible outcomes within the model. We will use two ways to calculate *p* values.

The common definition is used as a first possibility, assuming the validity of normal and all independent pdf’s. It consists in the evaluation of the cumulative of the $\chi ^2$-distribution—the latter denoted by $f(x, N_\mathrm{dof})$, with $N_\mathrm{dof}$ number of degrees of freedom—starting from the value $\chi ^2_* = -2 \log \mathcal{L}(\varvec{\theta }_{\varvec{*}})$

B10 $$\begin{aligned} p = \int ^{\infty }_{\chi ^2_*} \mathrm{d}x \, f(x, N_\mathrm{dof}) , \end{aligned}$$

and corresponds to the probability of observing a test statistic at least as extreme in a $\chi ^2$ distribution with $N_\mathrm{dof}$. Values of $p < 5~\%$ are usually referred to as “statistical significant” deviation from the null hypothesis, i.e., the validity of the model with parameters $\varvec{\theta }_{\varvec{*}}$. As usual, the number of degrees of freedom is given as $N_\mathrm{dof} = (N_\mathrm{meas} - \text{ dim } (\varvec{\theta }))$, with $N_\mathrm{meas}$ denoting the number of measurements.

As a second possibility we calculate the *p* value defining a test statistics based on the likelihood [86]. The according frequency distribution is determined from $10^6$ pseudo experiments in the lack of raw data and experimental efficiency corrections that require dedicated detector simulations. For this purpose, the pdf of each observable $O_i$ is shifted such that the position of its maximum at $O_i = O_i^\mathrm{exp}$ coincides with the prediction $O_i^\mathrm{th} (\varvec{\theta }_{\varvec{*}})$ at the point $\varvec{\theta }_{\varvec{*}}$ of interest. In this way, the uncertainties of the measurement with central value $O_i^\mathrm{exp}$ are adopted for $O_i^\mathrm{th} (\varvec{\theta }_{\varvec{*}})$, neglecting possibly different experimental efficiency corrections. In each pseudo experiment, possible experimental outcomes are drawn for all measurements in the data set from the shifted pdf’s and the likelihood value is compared to that of the observed data set, determining this way the fraction of pseudo experiments with smaller likelihood values. The *p* value is identified with this fraction, however, for the number of degrees of freedom that corresponds to the number of measurements $N_\mathrm{meas}$ in the data set. Subsequently, we correct the *p* value by converting it to a $\chi ^2$ value with the help of the inverse cumulative distribution with $N_\mathrm{meas}$ degrees of freedom and recalculate it for the actual $N_\mathrm{dof}$ [87] using (B10).

### Appendix B.4: Probability distributions of observables

If certain observables are not yet measured or despite an existing measurement are not included in the data set *D* of the fit, one might obtain a prediction of its probability distribution given the data *D* and model *M* [86]. We calculate the considered observables at each point of the Markov Chain for the current value of $\varvec{\theta }$ and determine the interval of the theory uncertainty $[O_i^\mathrm{th} - {\varDelta }^-_i,\, O_i^\mathrm{th} + {\varDelta }^+_i]$ due to nuisance parameters as described in Appendix B.1. The obtained intervals are used to fill a histogram that is normalized eventually to obtain a probability distribution.

## Footnotes

1 Here $MM = PV$ stands for both, $MM = PV$ and $MM = VP$.

2 In principle one might introduce a separate $X_H$ for each meson $M_1$ and $M_2$ as well as for each operator insertion.

3 The classification of decays into $M_1M_2 = PV$ and *VP* refers to the amplitude $\alpha _4^c(M_1 M_2)$, which indeed exclusively occurs in that combination in all decay amplitudes of both decay systems. Nevertheless, some other $\alpha _i$ with $i\ne 4$ also contain contributions in which the pseudoscalar and vector mesons are interchanged.

4 A good description of the experimental data of Set I observables has also been found in an amplitude fit [62].
